# Serum SELENBP1 and VCL Are Effective Biomarkers for Clinical and Forensic Diagnosis of Coronary Artery Spasm

**DOI:** 10.3390/ijms232113266

**Published:** 2022-10-31

**Authors:** Xinyi Lin, Zijie Lin, Xin Zhao, Zheng Liu, Chenchao Xu, Bokang Yu, Pan Gao, Zhimin Wang, Junbo Ge, Yiwen Shen, Liliang Li

**Affiliations:** 1 Department of Forensic Medicine, School of Basic Medical Sciences, Fudan University, Shanghai 200032, China; 2 Department of Cardiology, Shanghai Institute of Cardiovascular Diseases, Zhongshan Hospital, Fudan University, Shanghai 200032, China

**Keywords:** coronary artery spasm, sudden cardiac death, SELENBP1, VCL, biomarker

## Abstract

Coronary artery spasm (CAS) plays an important role in the pathogenesis of many ischemic heart entities; however, there are no established diagnostic biomarkers for CAS in clinical and forensic settings. This present study aimed to identify such serum biomarkers by establishing a rabbit CAS provocation model and integrating quantitative serum proteomics, parallel reaction monitoring/mass spectrometry-based targeted proteomics, and partial least-squares discriminant analysis (PLS-DA). Our results suggested that SELENBP1 and VCL were potential candidate biomarkers for CAS. In independent clinical samples, SELENBP1 and VCL were validated to be significantly lower in serum but not blood cells from CAS patients, with the reasons for this possibly due to the decreased secretion from cardiomyocytes. The areas under the curve of the receiver operating characteristics (ROC) analysis were 0.9384 for SELENBP1 and 0.9180 for VCL when diagnosing CAS. The CAS risk decreased by 32.3% and 53.6% for every 10 unit increases in the serum SELENBP1 and VCL, respectively. In forensic samples, serum SELENBP1 alone diagnosed CAS-induced deaths at a sensitivity of 100.0% and specificity of 72.73%, and its combination with VCL yielded a diagnostic specificity of 100.0%, which was superior to the traditional biomarkers of cTnI and CK-MB. Therefore, serum SELENBP1 and VCL could be effective biomarkers for both the clinical and forensic diagnosis of CAS.

## 1. Introduction

Coronary artery spasm (CAS) refers to an intense vasoconstriction of the coronary arteries that causes total or subtotal vessel occlusion. Since the original report by Prinzmetal et al. in 1959 [1], CAS has now been considered to play an important role in the pathogenesis of ischemic heart diseases, including stable angina, acute coronary syndrome (ACS), and sudden cardiac death (SCD) [2]. The frequency of CAS varies across countries, with East Asian populations (particularly Japanese) generally higher than the Western populations [3]. Based on the studies of Asian populations, the prevalence of CAS is around 50% in patients with angina and 57% in patients with ACS [4,5], and approximately 3.6% of CAS patients with normal or near normal coronary arteries died from cardiac causes [6].

In the clinic, CAS may present in a variety of ways and is often asymptomatic. It is for this reason that the diagnosis of CAS is often delayed, with an estimated delay of 3 months from presentation to diagnosis [7]. Moreover, symptomatic ischemia induced by a CAS attack is often indistinguishable from ischemia originating from other causes based on clinical complaints (i.e., chest pain and arrhythmias). It is thus important to conduct electrocardiogram (ECG) monitoring in patients with suspected CAS. However, even with ambulatory ECG monitoring, the attack may not appear during the monitoring periods, especially when the attack is not frequent [8,9]. Moreover, ECG does not provide direct or specific evidence of CAS [10]. It is for these reasons that provocation tests with the aid of coronary angiography have been recommended as the gold standard for a definitive diagnosis [11,12]. Notably, despite the usual safety of provocation tests [13], these are still not commonly performed due to a number of potential complications, such as various arrhythmias, hypertension, hypotension, and nausea [13]. Recently, non-invasive biochemical markers have been found to associate with the occurrence of CAS, including inflammatory factors, Lipoprotein a, Cystatin C, Serotonin, Endothelial-1, etc. [14]. Xanthine oxidoreductase activity, which plays a pivotal role in producing both uric acid and ROS, has also been reported to associate with CAS in clinical patients [15]. Endoreticulumn stress (ERS)-related secretory proteins such as valosin containing the protein (VCP), a vesicular integral membrane protein 36 (VIP36, also known as LMAN2), and Calpain-1 (CAPN-1) were considered as potential serological biomarkers for diagnosis of CAS-induced acute ischemia in our previous studies [16,17]. These studies led to transitions from a standard yet risky diagnostic strategy to a non-invasive biochemical diagnostic detection.

In forensic practice, ischemic heart disease is a prevailing cause of SCDs, with atherosclerotic cardiovascular diseases (ASCVD) being the most recurrent substrate [18]. The triggering factors leading from ASCVD to the onset of SCD generally include transitory coronary spasms and prolonged occlusive coronary stenosis [19]. In cases of CAS-induced sudden deaths, several morphological changes within the coronary artery wall have been shown to reflect the occurrence of antemortem CAS. These changes included the smooth muscle contraction bands in the media of the coronary arteries [20], a typical lengthwise shortening of smooth muscle cells and the squeezing and folding of the nuclei [21], and the presence of internal elastic membrane and intimal folds [22]. However, identifying these morphological changes is subjective and has interobserver variability. The CAS-induced sudden death is still hard to verify through anatomic observations. Thus, the medico-legal diagnosis of CAS-induced SCD is still made per exclusionism in real casework [23]. 

Ideally, a good diagnostic strategy for CAS would be highly effective and minimally invasive. Since unequivocal biomarkers allow for the objective diagnosis of CAS, a serum proteome-wide screening of CAS-associated biomarkers would provide novel insights into the clinical and forensic diagnosis of CAS. We have previously shown that phosphorylated myosin light chain 2 (p-MLC2) may serve as a coronary marker of antemortem CAS [24] and aberrant endoplasmic reticulum stress-mediated CAS occurrence through regulating the MLCK/MLC2 pathway [25]. The present study aimed to further identify potential serum biomarkers for CAS diagnosis. Due to the difficulty of enrolling patients for provocation tests in the clinic, we established a rabbit CAS provocation model that allowed for the collection of serum samples while coronary arteries were undergoing spastic constriction. The serum of each rabbit before and after intracoronary drug provocation was collected and subjected to quantitative proteomics for screening, followed by a parallel reaction monitoring/mass spectrometry (PRM/MS)-based targeted proteome validation and partial least-squares discriminant analysis (PLS-DA). Clinical and forensic samples were then investigated for the validation of candidate biomarkers. Our multiple lines of evidence suggested that serum SELENBP1 and VCL could be easy-to-prepare and non-invasive biomarkers for both the clinical and forensic diagnosis of CAS.

## 2. Results

### 2.1. Proteins Identified with the CAS Provocation Model

**Figure 1:**
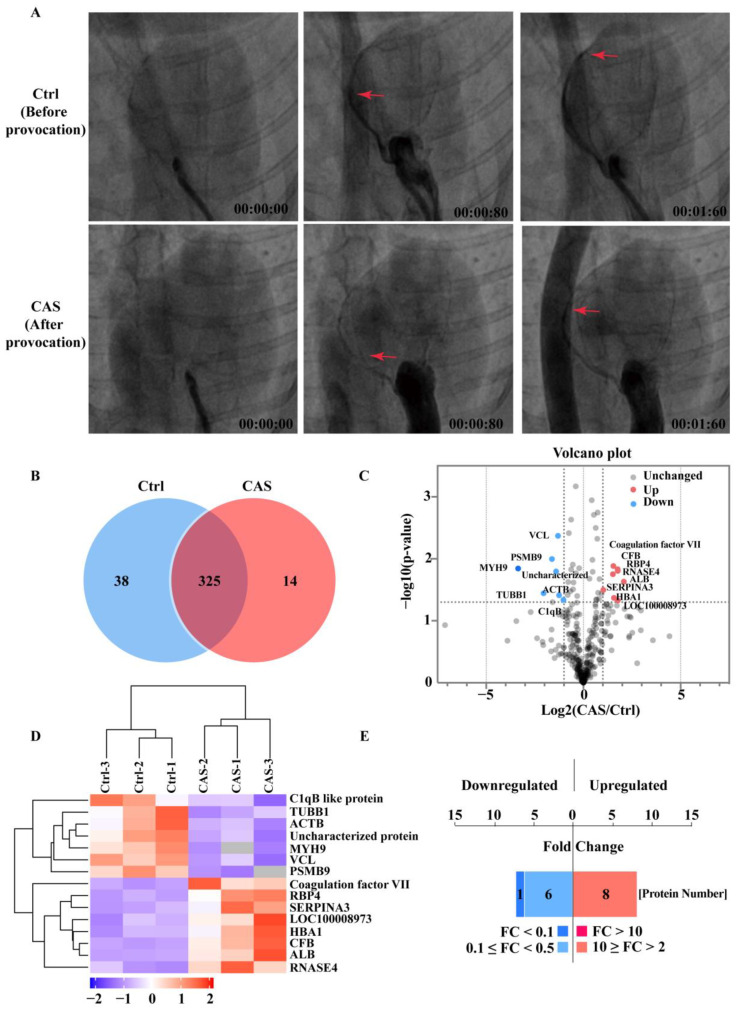
Serum proteome identified multiple dysregulated proteins in the rabbit coronary artery spasm (CAS) provocation model. (**A**) Representative coronary angiogram showing the blood flow before (Ctrl) and after (CAS) pituitrin intracoronary provocation. Red arrows indicate the blood flow at the indicated time points. Time = minute: second: millisecond. (**B**,**C**) Venn and volcano plots showing the distribution of identified serum proteins. Fifty-two proteins were specifically expressed, while 325 proteins were shared by both groups of the serum. (**D**) At a threshold of fold-change ≥2 and *p* value < 0.05, 15 proteins were identified as significantly differential proteins and are shown in a hierarchical clustering heat map. (**E**) A bar chart showing the fold-change distribution of the 15 significantly differential proteins.

The serum proteome identified multiple proteins that were dysregulated in the rabbit CAS provocation model. The representative coronary angiograms of the rabbit before (the control) and after (CAS) provocation in the sequence of time are displayed in Figure 1. It shows how, before the provocation agent Pituitrin injection, the rabbits’ coronary arteries displayed normal blood perfusion. However, after the Pituitrin injection, occlusive spasms with diffuse vasoconstriction in the curved proximal segment of the coronary were observed (Figure 1A). Based on the provocation model, the control (before provocation) and CAS (after provocation) serum were then prepared and subjected to label-free proteome analysis. A total of 377 serum proteins were identified, among which 325 (86.2%) proteins were shared by both the control and CAS groups (Figure 1B and Table S1). The remaining 52 (13.8%) proteins were specifically expressed, 14 of which were specific to the CAS group, and the remaining 38 proteins were specific to the control group (Figure 1B). In particular, the three ERS-related proteins (VCP, LMAN2, and CAPN-1), which we previously showed to associate with CAS-induced acute ischemia [16,17], were among the 52 specific proteins that supported the successful establishment of the CAS provocation model. To capture the greatest possible serum proteins, we used a rigorous criterion (with a fold change ≥2 and adjusted *p*-value < 0.05) for protein sorting. Volcano plots showed the global distribution of the 325 shared proteins (Figure 1C). A total of 15 serum proteins were significantly different between the two groups. Among those 15 proteins, 8 proteins were significantly upregulated, and 7 proteins were significantly downregulated (Figure 1C). These significantly altered proteins included coagulation factor VII, the complement component, and serum constituents, such as albumin and globin (Figure 1C), all of which are associated with the process of coronary vasoconstriction [26] and, in turn, support the successful provocation of CAS in our rabbit model. The 15 significantly differential proteins are further depicted in a hierarchical clustering heat map (Figure 1D) and displayed in bar charts (Figure 1E).

**Figure 2:**
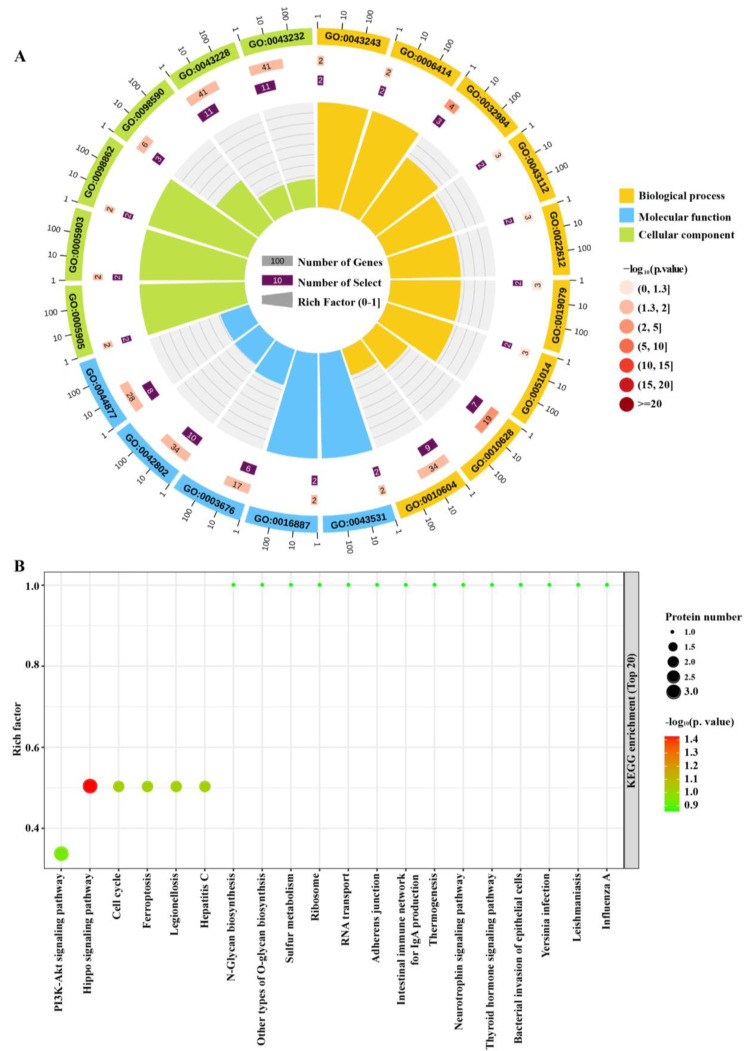
Bioinformatics analyses of the differentially expressed (DE) proteins. Serum proteome identified multiple dysregulated proteins in the rabbit CAS provocation model. (**A**) GO enrichment analysis of the 67 DE proteins. Proteins associated with biological processes (BP), molecular functions (MF), or cellular components (CC) are shown in orange, blue, or green, respectively. (**B**) KEGG analysis of the 67 DE proteins.

Based on the 67 differentially expressed (DE) proteins (52 specific proteins and 15 significantly differential proteins), the Gene Ontology (GO) and Kyoto Encyclopedia of Genes and Genomes (KEGG) enrichment analyses were then performed (Tables S2 and S3). In the Go enrichment analysis (Figure 2A), biological processes (BP), such as the protein-containing complex disassembly (GO ID: 0032984) and positive regulation of gene expression (GO ID: 0010628), were significantly enriched. The molecular function (MF), such as ADP binding (GO ID: 0043531) and ATPase activity (GO ID: 0016887) that critically dominate the energy supply of contraction rhythms were highly enriched. The plasma membrane region (GO ID: 0098590) was enriched in the cellular component (CC) analysis, providing supportive evidence of our previous reports that membrane organelles, such as endoplasmic reticulum (ER), were pivotal intracellular sites for the development of CAS [25]. Moreover, multiple processes involving protein binding/disassembly, such as the protein-containing complex disassembly in the BP analysis (GO ID: 0032984), identical protein binding (GO ID: 0042802), and protein-containing complex binding (GO ID: 0044877) in the MF analysis, and non-membrane-bounded organelle in the CC analysis (GO ID: 0043228) were significantly enriched (Figure 2A). In the KEGG analysis (Figure 2B), multiple common pathways such as PI3K-Akt signaling, the Hippo signaling pathway, cell cycles, and ferroptosis were top enriched. Multiple inflammation-associated pathways such as Hepatitis C, the bacterial invasion of epithelial cells, and Influenza A were also enriched (Figure 2B), providing further support for the association of perivascular inflammation with coronary spasms [27,28,29]. Consistent with the GO analysis, pathways associated with protein synthesis, such as Ribosome and RNA transport, were also enriched in the KEGG analysis (Figure 2B). These bioinformatic analyses verified the classical mechanisms of CAS pathogenesis, and also implied protein-binding and synthesis processes as novel pathways associated with CAS development.

### 2.2. PRM/MS-Based Targeted Proteome and PLS-DA Algorithm Validated SELENBP1 and VCL as Top Dysregulated Serum Proteins

**Figure 3:**
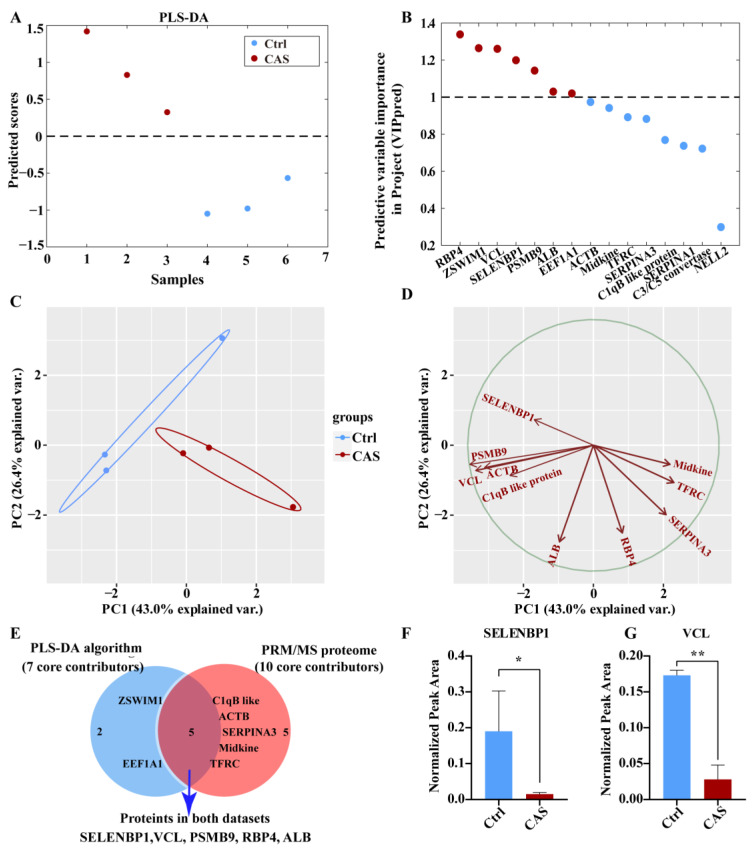
Parallel reaction monitoring/mass spectrometry (PRM/MS)-based targeted proteome and partial least-squares discriminant analysis (PLS-DA) validated SELENBP1 and VCL as the top dysregulated proteins. (**A**) Discrimination power of the quantified 15 candidate proteins from Ctrl to CAS analyzed by PLS-DA algorithm. (**B**) Seven candidate proteins (VIPpred > 1) were the core contributors to discrimination by predictive variable importance in projection (VIPpred) analysis (Red dots: VIPpred > 1; Blue dots: VIPpred < 1). (**C**,**D**) Principal component analysis (PCA) showed that the top 10 proteins could discriminate CAS from Ctrl serum. (**E**) Cross-analysis of the results from PRM/MS proteome and PLS-DA results. (**F**,**G**) The validated contents of SELENBP1 and VCL in CAS and Ctrl serum. *, *p* < 0.05; **, *p* < 0.01 as indicated.

To further narrow down the candidate proteins, we assessed the 67 DE proteins and found that 40 proteins could potentially be validated by the PRM/MS targeted proteome because they possessed ascertained names and unique peptides. After PRM/MS targeted detection, 15 of the 40 proteins were precisely quantified. Using PLS-DA algorithm, 15 of the quantified proteins could nicely distinguish CAS from the Ctrl (Figure 3A). Seven of them, including RBP4, ZSWIM1, VCL, SELENBP1, PSMB9, ALB, and EEF1A1, were identified as the core contributors to distinguishing normal vessels from the spastic coronary arteries (Figure 3B). We then compared the label-free proteome with the PRM/MS-based targeted proteome and found that 11 of the 15 precisely quantified proteins (73.3%) altered consistently between the dual omics. A principal component analysis (PCA) showed that the top 10 filtered proteins could effectively discriminate the CAS group from the non-CAS (control) group, with the two axes accounting for 69.4% of the total variability (Figure 3C). In particular, the principal component 1 (PC1), which alone accounted for 43.0% of the variability, was negatively correlated with six proteins (SELENBP1, PSMB9, VCL, C1qB-like protein, ACTB, and ALB) while positively correlated with the remaining four proteins (Midkine, TFRC, SERPINA3, and RBP4) (Figure 3D). Among the top 10 validated proteins, 4 proteins (SELENBP1, VCL, PSMB9, and ACTB) were the leading ones that showed maximal fold changes as per the PRM/MS validation analysis. After the integrative-analysis of the proteome-wide results and PLS-DA results, SELENBP1 and VCL were the core proteins that showed consistent differentiation capacities (Figure 3E). More specifically, SELENBP1 and VCL showed decreases by up to 8.98-fold and 4.33-fold, respectively, when compared with the control serum (Figure 3F,G). Thus, the proteins SELENBP1 and VCL were selected as the top dysregulated serum proteins. 

### 2.3. Genome-Wide Association Studies (GWAS) and Phenome-Wide Association Studies (PheWAS) Revealed Association of SELENBP1 and VCL Variations with Coronary Artery Diseases

**Figure 4:**
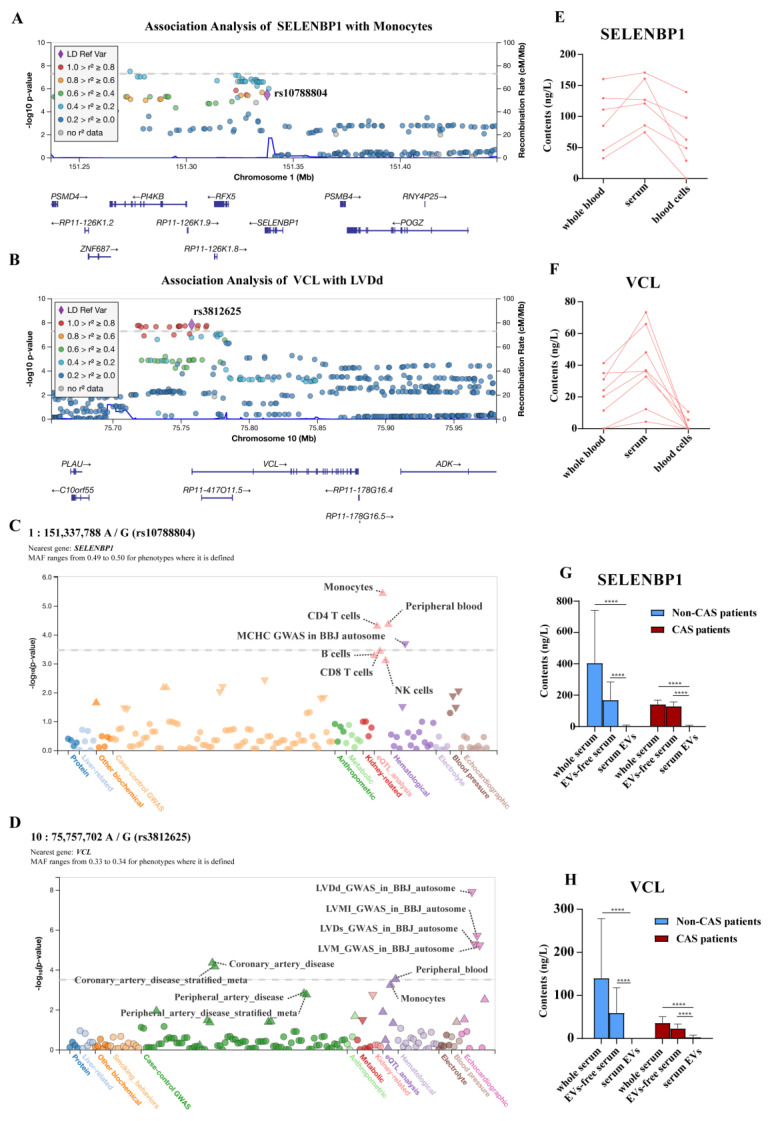
SELENBP1 and VCL were associated with coronary artery diseases and were abundantly distributed in extracellular vesicles (EVs)-free serum. (**A**) Genome-wide association study (GWAS) of *SELENBP1* sequence variants with monocyte aggregates. The common single nucleotide polymorphism (SNP) rs10788804, that was significantly associated with monocyte aggregates in humans, is indicated by a purple dot. (**B**) GWAS of *VCL* sequence variants with cardiac contractility. The common SNP rs3812625 that significantly associated with the left ventricle dimension at the diastole (LVDd) in humans is indicated by a purple dot. (**C**,**D**) Phenome-wide association studies (PheWAS) of the *SELENBP1* SNP rs10788804 and *VCL* SNP rs3812625 with coronary artery diseases. (**E**,**F**) Enzyme-linked immunosorbent assay (ELISA) detection of SELENBP1 and VCL in whole blood, serum, and blood cells. *n* = 6 for each group. (**G**,**H**) ELISA detection of SELENBP1 and VCL in whole serum, EVs-free serum, and serum EVs samples. *n* = 21 for non-CAS group and *n* = 25 for CAS group. ****, *p* < 0.0001 as indicated.

To gauge the potential roles of SELENBP1 and VCL in the development of CAS, we queried a summary of GWAS data from 563,085 European ancestry individuals [30]. This study identified an association between a common single nucleotide polymorphism (SNP) of *SELENBP1* (rs10788804) and monocyte aggregates (Figure 4A). In the view that coronary adventitial and perivascular adipose tissue inflammation has been shown to associate with vasospastic angina [27], this finding suggests that *SELENBP1* is a gene related to inflammatory cell infiltration-associated CAS development. We also queried another GWAS data of 162,255 Japanese individuals [31] and identified an association between the *VCL* polymorphism (rs3812625) and left ventricular dimension in the diastole (LVDd), suggesting that *VCL* is a gene related to cardiac function (Figure 4B). The basic information for the selected SNPs of *SELENBP1* and *VCL* is shown in Table S4. Moreover, in the PheWAS analysis, we found that the polymorphism of *SELENBP1* (rs10788804) was significantly associated with monocytes and several hematological phenotypes, such as peripheral blood, CD4+ T cells, CD8+ cells, B cells, and NK cells (Figure 4C). The common SNP rs3812625 of *VCL* was also significantly associated with coronary and peripheral artery diseases in the PheWAS analysis (Figure 4D). These studies further support the notion that sequence variations in *SELENBP1* and *VCL* have intimate associations with the development of coronary artery diseases.

### 2.4. SELENBP1 and VCL Were Abundantly Enriched in Extracellular Vesicles (EVs)-Free Serum Samples

To depict the blood tropism of the candidate biomarkers, we further carried out enzyme-linked immunosorbent assay (ELISA) to determine the levels of SELENBP1 and VCL in the whole blood and its constituents. The ELISA results showed that all six samples displayed higher levels of SELENBP1 and VCL in the serum than in the whole blood or blood cells (Figure 4E,F). To determine whether serum SELENBP1 and VCL were distributed in a free state or whether they were encapsulated by EVs, we collected serum samples from both the non-CAS patients and the CAS patients and extracted EVs from the serum. ELISA showed that SELENBP1 or VCL levels in the EVs-free serums were indistinguishable from those in the whole serum samples but significantly higher than their contents in the serum EVs samples (Figure 4G,H). These data suggested that SELENBP1 and VCL were abundantly enriched in the EVs-free serum samples. 

### 2.5. Lower Serum SELENBP1 and VCL Levels Were Due to Their Decreased Secretion from Cardiomyocytes under Contractile Conditions

To uncover why SELENBP1 and VCL were decreased in the blood samples from the CAS patients, four major cardiotropic cell types, including human cardiomyocytes (AC-16), fibroblasts (HFL1), endothelial cells (HUVEC), and vascular smooth muscle cells (HASMC), were treated with 0.5 mM acetylcholine (ACh) which is a commonly used reagent to trigger vasoconstriction in vitro or in the clinic [24]. After cell number normalization, SELENBP1 and VCL were found to secrete mostly from fibroblasts; however, their contents in both the cell lysates and supernatants (SNs) were not decreased after the stimulation of ACh (Figure S1). The cellular content of VCL in the AC-16 myocytes was also increased in response to ACh stimulation; however, the contents of both SELENBP1 and VCL in the SNs were notably decreased in the AC-16 myocytes but not in the remaining cell types (Figure S1). These observations suggest that decreased serum SELENBP1 and VCL contents in CAS conditions might be due to the dampened secretion from cardiomyocytes.

### 2.6. Diagnostic Potential of Serum SELENBP1 and VCL in Clinical CAS Patients 

We collected a total of 74 serum samples from patients who were diagnosed with CAS (*n* = 25) or non-CAS (*n* = 49). The baseline characteristics of the two groups of patients are shown in Table 1. It was found that nine variables, including sex, fast blood glucose, hs-CRP, cTNT, CK-MB, pro-BNP, albumin, SELENBP1, and VCL, were significantly different between the groups. The levels of these factors tended to be lower in the CAS group compared with the non-CAS individuals. 

Univariate and multivariate logistic regression analyses were then performed to assess the factors predicting the incidence of CAS (Table 2). In the univariate analysis, age (*p* = 0.081), blood glucose (*p* = 0.034), HbA1c (*p* = 0.093), cTnT (*p* = 0.084), CK-MB (*p* = 0.045), pro-BNP (*p* = 0.017), albumin (*p* = 0.045), and the newly identified SELENBP1 (*p* < 0.001) and VCL (*p* < 0.001) were associated with the incidence of CAS. The conventional CAS risk factors of hs-CRP (*p* = 0.026) and sex (*p* = 0.037), but not smoking (*p* = 0.963) or urine acid (*p* = 0.723), were also associated with the incidence of CAS. We thus incorporated 11 factors for multivariate analysis as their *p*-values were less than 0.10 in the univariate analysis. The results showed that only SELENBP1 and VCL were statistically significantly associated with CAS (Table 2). Binary logistic regression analysis further showed that the CAS risk decreased by 32.3% for each 10 ng/L increase in the serum SELENBP1 level (*p* = 0.004, Table 3), while it decreased by 53.6% for each 10 ng/mL increase in the serum VCL level (*p* = 0.006, Table 3).

**Figure 5:**
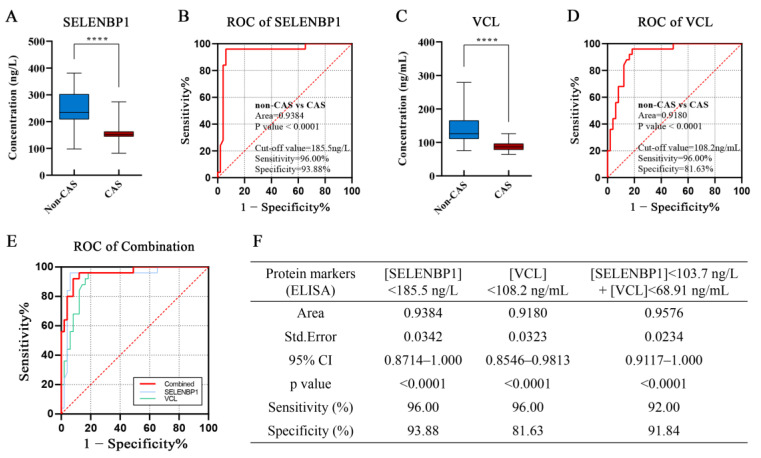
Serum SELENBP1 and VCL were effective biomarkers for clinical diagnosis of CAS. (**A**) Serum levels of SELENBP1 in non-CAS and CAS patients. (**B**) Receiver operating characteristics (ROC) analysis of serum SELENBP1 for diagnosis of CAS. (**C**) Serum levels of VCL in non-CAS and CAS patients. (**D**) ROC analysis of serum VCL for diagnosis of CAS. (**E**) ROC analysis of the combination of serum SELENBP1 and VCL for diagnosis of CAS. (**F**) A summary of the diagnostic efficacy in clinic for SELENBP1 and VCL alone or in combination. ****, *p* < 0.0001 as indicated. CI—confidence interval; Std. Error—standard error.

In particular, serum SELENBP1 levels were significantly lower in the CAS patients compared with the non-CAS individuals (*p* < 0.0001, Figure 5A). In terms of the diagnostic efficacy reported by the receiver operating characteristics (ROC) analysis, serum SELENBP1 yielded an area under the curve (AUC) of 0.9384 (*p* < 0.0001). At a cut-off value of 185.5 ng/L, SELENBP1 achieved a diagnostic sensitivity of 96.00% and a specificity of 93.88% (Figure 5B). Serum VCL levels were also significantly lower in the CAS patients in contrast to the non-CAS population (*p* < 0.0001, Figure 5C), and serum VCL yielded an AUC of 0.9180 (*p* < 0.0001). At a cut-off value of 108.2 ng/mL, VCL achieved a diagnostic sensitivity of 96.00% and a specificity of 81.63% (Figure 5D). When combining SELENBP1 and VCL, it displayed a higher AUC with similar diagnostic efficacy as serum SELENBP1 or VCL alone (Figure 5E,F). Moreover, when subdividing the non-CAS individuals into ASCVD patients and non-ASCVD populations, serum SELENBP1 and VCL levels did not differ within the two subgroups but were evidently higher than those in the CAS group (Figure S2), further supporting the diagnostic specificity of serum SELENBP1 and VCL levels for CAS. These studies collectively suggest that SELENBP1 and VCL are independent biomarkers for the clinical diagnosis of CAS. 

### 2.7. Diagnostic Potential of Serum SELENBP1 and VCL for CAS-Induced SCDs

**Figure 6:**
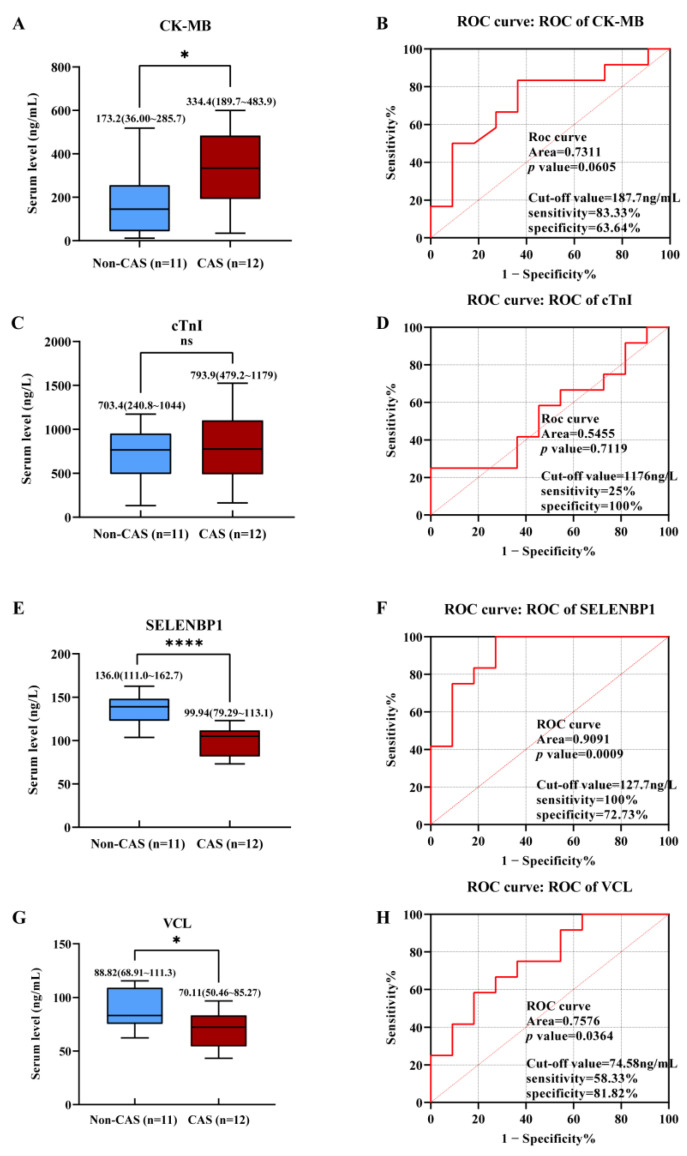
Serum SELENBP1 or VCL alone could be biomarkers for forensic diagnosis of CAS-induced sudden death. (**A**) Serum CK-MB levels in non-cardiac death (non-CAS) and CAS-induced sudden deaths. (**B**) ROC analysis of serum CK-MB for the diagnosis of CAS-induced sudden death. (**C**) Serum cTnI levels in non-cardiac death (non-CAS) and CAS-induced sudden deaths. (**D**) ROC analysis of serum cTnI for the diagnosis of CAS-induced sudden death. (**E**) Serum SELENBP1 levels in non-cardiac death (non-CAS) and CAS-induced sudden deaths. (**F**) ROC analysis of serum SELENBP1 for the diagnosis of CAS-induced sudden death. (**G**) Serum VCL levels in non-cardiac death (non-CAS) and CAS-induced sudden deaths. (**H**) ROC analysis of serum VCL for the diagnosis of CAS-induced sudden death. ns—no significant. *, *p* < 0.05; ****, *p* < 0.0001 as indicated.

We further assessed whether serum SELENBP1 and VCL could be applied to diagnose CAS-induced sudden deaths. We collected serum samples from 11 non-cardiac death cases (named the non-CAS group) and 12 CAS-induced death cases (named the CAS group). The baseline characteristics of the two groups are listed in Table 4. Initially, we found that postmortem serum CK-MB levels were significantly higher in the CAS group compared to the non-CAS group (*p* < 0.05, Figure 6A). Postmortem serum CK-MB levels diagnosed CAS-induced sudden death with an AUC of 0.7311 (*p* = 0.0605). At a cut-off value of 187.7 ng/mL, CK-MB yielded a diagnostic sensitivity of 83.33% and a specificity of 63.64% (Figure 6B). Unlike CK-MB, postmortem serum cTnI levels were indistinguishable between the CAS and non-CAS groups (Figure 6C). Serum cTnI yielded an AUC of 0.5455 (*p* = 0.7119). At a cut-off value of 1176 ng/L, cTnI yielded a diagnostic sensitivity of 25% and specificity of 100% (Figure 6D). Serum SELENBP1 levels were significantly lower in the CAS group compared to the non-CAS group (*p* < 0.0001) (Figure 6E). The serum SELENBP1 yielded an AUC of 0.9091 (*p* = 0.0009) and diagnosed CAS-induced sudden death at a cut-off value of 127.7 ng/L, a threshold that was remarkably lower than the cut-off value in clinical diagnoses (185.5 ng/L). In addition, serum SELENBP1 diagnosed antemortem CAS with a sensitivity of 100% and a specificity of 72.73% (Figure 6F). Similar to SELENBP1, serum VCL levels in CAS-induced sudden deaths were also significantly lower than those in non-cardiac deaths (Figure 6G). ROC analysis showed that the AUC of VCL was 0.7576 (*p* = 0.0364). When diagnosing CAS-induced sudden deaths, the VCL yielded a cut-off value of 74.58 ng/mL with a sensitivity of 58.33% and a specificity of 81.82% (Figure 6H). 

Of note, the mean values of serum SELENBP1 and VCL for the forensic death cases were both lower than those in living patients, an observation that reinforced the negative association between serum SELENBP1 or VCL levels and CAS severity. In fact, binary logistic regression analysis showed that the risk of CAS-induced deaths decreased by 71.7% and 49.4% for every 10-unit increase in serum SELENBP1 and VCL, respectively (Table 5). 

**Figure 7:**
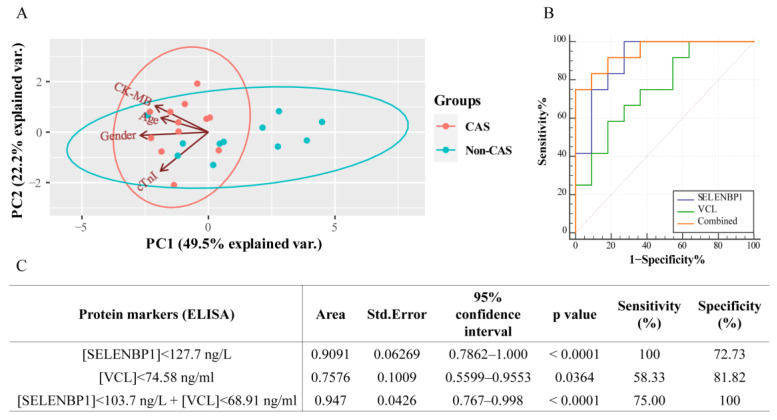
Combined SELENBP1 and VCL conferred improved diagnostic efficacy for forensic practice. (**A**) PCA showed that the combination of decedents’ age, gender, CK-MB, and cTnI levels did not allow discrimination between CAS-induced sudden death and non-cardiac death. (**B**,**C**) A comparison of the diagnostic efficacy in forensic cases for SELENBP1 and VCL alone and in combination.

Moreover, the PCA study found that information, including the decedents’ age, gender, serum CK-MB levels, and cTnI levels, could not discriminate CAS-induced deaths from non-cardiac control deaths (Figure 7A). ROC analysis showed that a combination of serum SELENBP1 and VCL yielded better diagnostic efficacy than a single protein (Figure 7B). Specifically, a threshold of 127.7 ng/L for SELENBP1 alone discriminated CAS from non-CAS with a sensitivity of 100% and a specificity of 72.73%. For VCL alone, the threshold was 74.58 ng/mL, with a sensitivity of 58.33% and a specificity of 81.82%. When combined, SELENBP1, with a threshold of 103.7 ng/L, and VCL, with a threshold of 68.91 ng/mL, yielded a sensitivity of 75.0% and a striking specificity of 100% (Figure 7C).

## 3. Discussion

CAS is a common cause of various cardiac entities, including stable angina, ACS, and SCD, particularly in East Asia [2,32]. Medicinal treatments and clinical manifestations are different between CAS and other trigger-induced angina. Thus, a definitive diagnosis of CAS is important for clinical management. Clinically, the gold-standard approach to diagnosing CAS is documentation by angiography, where stenosis caused by atherosclerotic plaque can be differentiated from stenosis caused by CAS, and the administration of intracoronary nitroglycerin, with luminal stenosis resolution, can support the diagnosis of CAS [33]. However, spontaneous coronary vasospasm at the time of angiography is only occasionally observed [2]. It is for this reason that provocation tests are commonly used to diagnose coronary vasospasm. Unfortunately, invasive provocative spasm testing is associated with complications and should thus be performed in a specialized center after careful evaluation of the risks and benefits [33]. The diagnosis of CAS-induced SCDs in forensic practice is even more challenging because only morphological changes to the spastic coronary arteries can be observed in a postmortem examination [20,21,22]. 

In view of the difficulty of obtaining serum samples while patients are undergoing coronary spastic activity [2], we alternatively established a rabbit CAS provocation model, which allowed for the self-control observation of the coronary artery vasoconstriction as it occurred in real-time. The serum samples collected before and immediately after the development of CAS were thus highly and clinically relevant. With this rigorously designed model, the collected sera were then analyzed by proteome-wide analyses, namely, the initial label-free proteome and secondary PRM/MS-based targeting proteome. After protein sorting and validation, our study provided multiple lines of evidence that serum SELENBP1 and VCL were definitive biomarkers for both the clinical and forensic diagnosis of CAS. In the proteome-wide analysis, multiple proteins, including coagulation factor VII, the complement component, and serum constituents, such as albumin and globin, were associated with the molecular mechanisms of coronary vasoconstriction [26]. ERS-related secretory proteins (i.e., VCP, LMAN2, and CAPN-1) that have been shown to associate with acute myocardial ischemia were also identified [16,17]. ADP binding (GO ID: 0043531) and ATPase activity (GO ID: 0016887), which commonly dominate the energy supply of contraction rhythms, were highly enriched in the GO analysis. Multiple inflammation-related pathways were enriched by bioinformatics analyses. These data reinforced the reliability of our established rabbit model. Of particular interest, to strengthen the significance of inflammation signaling and protein-binding processes, SELENBP1 and VCL, two proteins associated with protein-binding or inflammation, were validated to associate with CAS development, with their decreased serum levels being possibly attributed to damped secretion from cardiomyocytes. SELENBP1, a selenium-binding protein, directly binds with glutathione peroxidase and, thereby, potentially affects intracellular selenium metabolism and redox regulation [34]. SELENBP1 has been functionally involved in tumor prognosis and malignancy, and evidence has indicated SELENBP1 to be a tumor suppressor [35]. SELENBP1 involvement in cardiovascular diseases has not received attention until recently, where circulating levels of SELENBP1 have been found to associate with a risk for major adverse cardiac events and death [34]. VCL plays a fundamental role in integrin-mediated cell adhesion and intracellular tension [36]. Our GWAS and PheWAS analyses revealed that a sequence variation in *SELENBP1* was significantly associated with blood monocyte counts. Peripheral monocyte count is an independent marker for predicting CAS [37], and coronary adventitial and perivascular adipose tissue inflammation has been shown to associate with vasospastic angina [27]. Therefore, our data strengthened the notion that SELENBP1 levels are associated with inflammatory states and may extend its application into the diagnosis of CAS other than tumor biology. GWAS and PheWAS analyses also revealed that an SNP variation in *VCL* is significantly associated with left-ventricle dysfunctions. As a cell–cell and cell-matrix adhesion, VCL loss-of-function variations have been extensively evidenced to associate with inherited cardiomyopathy [38,39]. Hence, these combinatorial data suggested that SELENBP1 and VCL had functional associations with CAS. 

Moreover, we further provided evidence that serum SELENBP1 and VCL were effective biomarkers for diagnosing CAS in both clinical and forensic scenarios. In the clinical samples, it was found that fast blood glucose, HbA1c, CK-MB, cTnT, hs-CRP, and pro-BNP were significantly different between the groups, with their levels being lower in the CAS patients. This is different from the forensic results, which showed the CK-MB levels to be significantly increased in CAS-induced sudden deaths. This controversy might be due to non-identical control samples between clinical and forensic detection. High levels of CK-MB, cTnT, hs-CRP, and pro-BNP might be explained by the fact that the non-CAS group included patients with cardiovascular diseases which may still cause severe myocardial injuries and inflammation, and a substantial proportion of ASCVD patients had emerging acute myocardial infarction, whereas it was less frequent in non-cardiac death cases. Cigarette smoking, which has a significant association with CAS [40], was not absolutely high in the CAS group. This is because we could not judge the smoking frequency for the clinically collected patient, whilst only active and heavy smokers constituted the majority of CAS patients [40]. The contents of SELENBP1 and VCL specifically decreased in the CAS patients, and the association of SELENBP1 and VCL levels with CAS development was further established when their serum levels in fatal cases were significantly lower than those in living patients. Therefore, serum SELENBP1 and VCL levels might confer prognostic values for CAS in the clinic. High serum levels indicate a good prognosis, while low levels indicate the progression of CAS and might even suggest the probability of sudden death if left uncontrolled. Indeed, when discriminating CAS from non-CAS individuals, serum SELENBP1 yielded a diagnostic sensitivity of 96.00% and specificity of 93.88%, while VCL yielded a diagnostic sensitivity of 96.00% and specificity of 81.63%. Binary logistic regression analysis showed that the CAS risk decreased by 32.3% for every 10 ng/L increase of serum SELENBP1 (*p* = 0.004) and by 53.6% for every 10 ng/mL increase of serum VCL (*p* = 0.006) in the clinic. In forensic practice, while CK-MB, cTnI, and the decedents’ age and gender did not assure discrimination between CAS-induced sudden death and non-cardiac deaths, serum SELENBP1 alone or in combination with VCL conferred a good diagnostic sensitivity and specificity. This might be explained by the fact that the traditional myocardial injury markers, CK-MB and cTnI, were passively and non-specifically released into extracellular spaces in response to cardiac ischemic injuries and other factors such as global asphyxiation, hyperthermia, cerebrovascular disease, carbon monoxide intoxication, electrocution, fatal methamphetamine abuse, and psychotropic drug intoxication [41,42,43]. Our results suggest that serum SELENBP1 is appropriate for the primary screening of potential CAS-related cases in forensic practice due to its perfect sensitivity, and the combination of serum SELENBP1 and VCL could distinguish specifically CAS-induced deaths from others. The cumulative evidence suggests that SELENBP1 and VCL are effective biomarkers for CAS, particularly for the diagnosis of CAS-induced sudden deaths, since there have been no specific markers identified and used in forensic practice till now. 

In addition to having a good specificity to CAS conditions, both SELENBP1 and VCL were superior biomarkers because of their abundance in the serum, in contrast to the blood cells or serum EVs. This finding suggests that the preparation of whole serum samples would be sufficient to meet detection requirements without the need for the additional isolation of blood cells or serum EVs. EVs have been implicated in the clinical diagnosis of various diseases [43], particularly in cancer diagnoses [44]. However, the application of EV-derived biomarkers in clinical use is challenging because of limitations, such as EV isolation and data analysis [45]. While whole serum samples are commonly prepared in routine clinical work, and the collection of the serum assures minimal invasiveness for patients, the high abundance of SELENBP1 and VCL in whole serum samples made it easy to prepare samples and identify them as superior biomarkers that could be easily translated for use. 

There are several limitations to this study. First, clinical validation studies were based on relatively small sample sizes. A larger cohort-based study would strengthen the conclusion. Second, we used forensic samples with an early postmortem interval (approximately 2 h). The stability of SELENBP1 and VCL in the postmortem serum samples undergoing longer postmortem intervals need to be further investigated to extend their forensic application. Third, mechanisms underlying the decrease of serum SELENBP1 and VCL in CAS conditions remain largely unclear, while our preliminary results showed it may be attributed to their decreased secretion from cardiomyocytes under contractile conditions. It merits further in-depth investigation of their secretory mechanisms. Finally, the PRM/MS targeted validation was performed only on selected proteins due to technical limitations. Other serum proteins that were not validated might also be potential diagnostic biomarkers and thus merit further investigation.

In conclusion, the present study identified and validated serum SELENBP1 and VCL as potential biomarkers for the clinical and forensic diagnosis of CAS based on multiple lines of evidence gathered from the proteome-wide analysis, PLS-DA algorithm, GWAS and PheWAS analysis, and authentic sample analysis. SELENBP1 and VCL were abundant in the whole serum samples and were specifically decreased in CAS conditions, making these serum proteins specific biomarkers for CAS diagnosis. Higher levels of SELENBP1 or VCL might suggest a good prognosis in both clinical and forensic scenarios.

## 4. Materials and Methods

### 4.1. Experimental Design

To collect serum samples while the coronary arteries were undergoing spastic constriction, we used a rabbit CAS provocation model with the aid of coronary angiogram monitoring. To minimize inter-individual variability, we collected the serum of each rabbit before and after CAS provocation. This self-control approach reliably allowed the observation of CAS progression and the capture of DE serum proteins in a real-time manner. The prepared serum samples were then subject to a label-free quantitative proteome for the screening of DE proteins, followed by protein-targeted validation using the PRM/MS technique and PLS-DA algorithm. GWAS and PheWAS were also performed to determine the functional association of the serum candidate biomarkers with coronary artery diseases. The candidate proteins were then applied to authentic clinical and forensic samples. The diagnostic efficacy of these biomarkers was assessed by ROC curves.

### 4.2. Animal Experiments

To set up the in vivo rabbit CAS provocation model, Pituitrin was used to trigger coronary spastic activity in animal models. Pituitrin is extracted from the pituitary gland that contains vasopressin and oxytocin. Pituitrin is able to affect circulation and cause cardiac ischemia by inducing vascular contraction [19] and, therefore, has been widely used to create a CAS model [20,21]. We have previously established a CAS provocation model by the intraperitoneal (i.p.) injection of Pituitrin (5.1 U/kg) into mice with normal coronary arteries [25]. To minimize the unexpected constriction of the vessels beyond the coronary artery, we modified the drug-delivery method by injecting the drug at the root of the rabbit’s aorta, which created a local coronary artery response and was clinically relevant. Methods for the angiography and induction of vasospasms were in accordance with a previous description [46]. Briefly, three healthy New Zealand white rabbits (weighing 2.5–3.0 kg) were purchased from the Laboratory Animal Science Center of Fudan University (Shanghai, China) and housed in a temperature-controlled room at 20–22 °C under a 12 h light/dark cycle with free access to food and water. All rabbits were allowed to acclimate for 1 week before any treatment. For the provocation tests, the rabbits were anesthetized with sodium pentobarbital (30 mg/kg i.v.) while intubated and ventilated on a small-animal respirator (Harvard Apparatus) with oxygen and 2% isoflurane. Body temperature was maintained with a water-jacketed heating pad, and arterial blood gases were monitored. Blood pressure and heart rate were continuously monitored with a noninvasive cuff (Criticon Dinamap Research Monitor, Criticon). Through the access of carotid artery 4F guiding catheters, the operators conducted blood collection and nonselective coronary arteriography in the root of the aorta. An average of 5 mL of 65% iohexol contrast was manually injected into each arteriography. For the purpose of self-control, the procedures were performed before and after the Pituitrin (1.3 U/kg) injection through the carotid artery-advanced catheter. All experimental procedures were approved by the Institutional Animal Care and Use Committees at the School of Basic Medical Sciences, Fudan University (approval No.: 20180302-049). All efforts were made to minimize animal suffering.

### 4.3. Clinical Sample Collection

All the blood samples were collected from patients admitted to the Zhongshan Hospital at Fudan University between August 2020 and February 2021. This study complied with the principles of the Declaration of Helsinki and was approved by the Ethics Committee of the Zhongshan Hospital, Fudan University (No.: B2020-078R). We collected clinical serum samples from 2 groups of subjects categorized as the CAS patients (*n* = 25) and the non-CAS patients (*n* = 49). Since the gold standard for the definite diagnosis of CAS-intracoronary provocation tests with the aid of coronary angiograph [26] was not commonly recommended in Western countries and in China for safety concerns [47,48], we modified the inclusion criteria for the CAS group with a comprehensive assessment of clinical symptoms, ECG changes, circadian patterns, and their responses to calcium channel blockers (CCBs). Patients with normal coronary arteries but manifesting recurrent resting chest pains and ECG ST-segment elevation, these patients were considered to be affected by spasms if the clinical scenario was consistent with the characteristics of CAS [49]. Patients with stenosed coronary arteries were also included in the CAS group if they showed significant relief of angiographic stenoses after the intracoronary administration of nitroglycerin [50] and presented with at least one of the following conditions: (1) an obvious resting chest pain, often from midnight to early morning; (2) high incidence of elevated ST segment during attacks; (3) symptom relief in response to CCB medication; (4) a typical history of active smoking. Patients under CCBs and other vasodilator treatments were included only after the discontinuation of medications for at least 5 days. The non-CAS group included patients with non-cardiac diseases (i.e., epilepsy, gastrointestinal bleeding, and chronic kidney diseases) or CAS-unrelated ASCVD and health checkup individuals. For each case, 1 mL of serum was collected from the time that the first medical contact was used. 

### 4.4. Forensic Sample Collection

To extend the potential application of candidate proteins into forensic practice, a total of 12 cases (mean age: 52.17 ± 4.06 years) who died from SCDs between December 2018 and October 2020 were investigated. All cases had a clear medical history of recurrent resting angina pectoris for six months prior to death. Before death, they presented with short periods of chest distress during midnight or the early morning and died shortly after a cardiac attack (mean: <2.58 ± 2.33 h). An autopsy was initiated approximately 2 h after death, where these cases were revealed to have only <50% coronary artery occlusion. Systemic autopsies excluded other possible causes of death. Vessels with less than 50% occlusion have been shown to be more commonly associated with the presence of contraction bands in the smooth muscle of the spastic coronary arteries [20]. Thus, these cases were designated as CAS-induced sudden deaths for the purpose of this study. Another 11 fatal cases that were age-matched (mean age: 42.73 ± 13.33 years) were also used for the control. These control cases died from traumatic death (*n* = 3 for sharp force injury and *n* = 3 for public transportation injury) or acute drug intoxication (alcohol or antipsychotics) with an average death time of <1.50 ± 0.50 h. We collected 1 mL of whole blood from each of the cases immediately after the notification of death. The protocol to use the human blood samples for research purposes was approved by the Institutional Review Board at the School of Basic Medical Sciences, Fudan University (approval No.: 2019-023). 

### 4.5. Serum Sample Preparation

Fresh blood samples were allowed to sit for free coagulation for 30 min at room temperature and were then centrifuged at 12,000× *g* rpm for 10 min at 4 °C to remove cells and cellular debris. After centrifugation, the resultant SNs (serum) were filtered through a 0.22 µm pore mesh, aliquoted in 1 mL tubes (500 µL per tube) and stored at −80 °C for subsequent assays. The blood cells were transferred to a clean tube until use.

### 4.6. Protein Extraction and Enzymolysis

High-abundance proteins were removed from the serum pools according to the protocol from the manufacturer. SDT (4% (*w*/*v*) SDS, and a 100 mM Tris-HCl pH 7.6, 0.1 M dithiothreitol) buffer was used for sample lysis and protein extraction. The serum proteins were then quantified using a BCA protein assay kit (Bio-Rad, Hercules, CA, USA). An appropriate amount of protein from each sample was digested by the filter-aided sample preparation (FASP) method and desalted using C18 cartridges (Empore™ SPE Cartridges C18, bed I.D. 7 mm, volume 3 mL, Sigma, St Louis, MO, USA). After being lyophilized, the digested proteins were concentrated by vacuum centrifugation and reconstituted in 40 µL of 0.1% (*v*/*v*) formic acid solution.

### 4.7. Liquid Chromatography-Tandem Mass Spectrometry (LC-MS/MS) Analysis

Label-free serum proteome characterization was performed at the Shanghai Applied Protein Technology Co. Ltd. (Shanghai, China) with the procedure reported previously [51]. Briefly, the peptides were loaded onto a reverse phase trap column (Thermo Scientific Acclaim PepMap100, 100 μm × 2 cm, nanoViper C18), connected to the C18-reversed-phase analytical column (Thermo Scientific Easy Column, 10 cm long, 75 μm inner diameter, 3 μm resin) in buffer A (0.1% Formic acid) and were separated with a linear gradient of buffer B (84% acetonitrile and 0.1% Formic acid) at a flow rate of 300 nL/min controlled by IntelliFlow technology. After chromatographic separation, the samples were analyzed on a Q Exactive mass spectrometer (Thermo Fisher Scientific, Waltham, MA, USA) that was coupled to Easy nLC (Thermo Scientific) for 60/120/240 min. After a full scan, the top 10 MS/MS events were acquired. The detection was operated in positive ion mode, with the survey scan of 300–1800 *m*/*z*. The primary mass spectrum was acquired at a resolution of 70,000 at 200 *m*/*z* with the automatic gain control (AGC) target of 3e6, a maximum inject time of 10 ms, and a dynamic exclusion duration of 40.0s. The resolution for the HCD spectra was set to 17,500 at *m*/*z* 200, and the isolation width was 2 *m*/*z*. The normalized 0collision energy was set as 30 eV and the underfill ratio was defined as 0.1%.

### 4.8. Protein Identification and Quantitative Analysis

The mass spectrometry raw data for each sample were combined and searched using the MaxQuant 1.5.3.17 software for identification and quantitation analysis, as previously described [52]. Proteins identified more than twice in the three biological replicates were defined as quantifiable proteins. The label-free quantification (LFQ) intensity of each protein was then statistically compared using the Student’s *t*-tests between the CAS and ctrl groups using Microsoft Excel 2016 software (Microsoft, Seatle, WA, USA). In our study, proteins with a *p*-value of less than 0.05 and more than a two-fold difference were classified as significantly differential proteins. The proteins that were undetectable in a specific group but quantifiable in their counterparts were considered to be specifically expressed proteins. Both the significantly differential proteins and specific proteins were pooled as DE proteins that were clustered for bioinformatical analysis. 

The label-free serum proteome has been deposited to the ProteomeXchange Consortium (http://proteomecentral.proteomexchange.org) via the iProX partner repository with the dataset identifier PXD036383.

### 4.9. Bioinformatics Analysis

For the bioinformatics analysis, GO and KEGG analyses were performed. In the GO enrichment analysis, the protein sequences of all DE proteins were locally searched using the NCBI BLAST+ client software (ncbi-blast-2.2.28+-win32.exe) (https://blast.ncbi.nlm.nih.gov/Blast.cgi/, accessed approximately on 3 September 2020) and InterProScan (version 5.52-86.0) (https://www.ebi.ac.uk/interpro/about/interproscan/, accessed approximately on 3 September 2020)) to find homolog sequences. Then, GO terms were mapped, and sequences were annotated using the software program Blast2GO (Version 2.8.0) (https://www.blast2go.com/, accessed approximately on 3 September 2020). The GO annotation results were then plotted using the OmicsShare tool (https://www.omicshare.com, accessed approximately on 23 May 2021). For the KEGG annotation, following the annotation steps, the studied proteins were blasted against the online KEGG database (http://geneontology.org/, accessed approximately on 3 September 2020) to retrieve their KEGG orthology identifications and were subsequently mapped to pathways in KEGG. The *p*-values of pathway enrichment were calculated as previously described [51]. 

### 4.10. LC-PRM/MS Analysis

To validate the protein abundance obtained by label-free mass spectrometry, we further performed LC-PRM/MS analysis for the selected proteins from the same samples at the Shanghai Applied Protein Technology Co., Ltd. (Shanghai, China). The raw data were analyzed with the bioinformatic tool Skyline (version 3.5.0) (MacCoss Lab, University of Washington, Seattle, WA, USA), where the signal intensities of the peptide sequences for the significantly altered proteins were quantified relative to each sample, and normalized to the standard reference.

### 4.11. GWAS and PheWAS Analyses

Summary GWAS data, incorporating 563,085 European ancestry individuals [30], were used to investigate the association between *SELENBP1* polymorphisms and monocyte aggregates. Another GWAS data, including 162,255 Japanese individuals [31], were used in the investigation of the association between the *VCL* polymorphisms and left ventricular contractility. The Bonferroni-corrected *p*-value threshold of 5.0 × 10^−8^ was used to assess the significance of the association analysis. Then, we assessed the sequence variants of *SELENBP1* and *VCL* with a potential pathogenic mechanism to CAS through the PheWAS analysis in JENGER (http://jenger.riken.jp, accessed on 28 March, 2021). The regional plots of GWAS and PheWAS were generated using PheWeb (version 1.1.18) in the JENGER.

### 4.12. PLS-DA Algorithm

The libPLS toolbox (version 1.95) operating in MATLAB version R2014a (The MathWorks, Natick, MA, USA) was used for PLS-DA. The protein of predictive variable importance in a projection larger than 1 (VIPpred > 1) was considered to be meaningful for sample discrimination. The source codes are available at www.libpls.net (accessed on 31 July 2021). All procedures were in accordance with the protocols described previously [53]. 

### 4.13. Serum EVs Isolation

The serum EVs were isolated using a commercial kit from Vazyme Biotechnology (Catalog No.: R602, Nanjing, China). Serum samples were thawed in a 25 °C water bath until they were completely thawed and were then centrifuged at 2000× *g* for 30 min at room temperature to remove the remaining cells and debris. An aliquot of 500 µL supernatant for each sample was then gently transferred into a new tube and was mixed with 100 µL of VEX EVs Isolation Reagent (Vazyme) for 30 min at 4 °C. After, the mixture was pelleted by ultracentrifugation at 10,000× *g* for 5 min. The precipitates (Evs) were then collected and washed twice with PBS and re-suspended in 100 µL of PBS for use. All isolation protocols were performed in strict accordance with the manufacturer’s instructions.

### 4.14. ELISA

To detect the serum protein contents, commercial ELISA kits were used. Samples were preliminarily diluted with saline by 2, 5, and 10-fold serial dilution, and it turned out that a 5-fold dilution yielded good results within the range of standards. Hence, all serum samples were thawed and diluted in a 1:5 ratio in this study. Briefly, an aliquot of 50 μL of the diluted sample, or the standard at different concentrations, was added to a 96-well microplate and incubated with a 100 μL HRP-conjugated secondary antibody at 37 °C for 1 h. Then, the unbound antibody was washed five times, and the wells were incubated with 100 μL/well enzyme substrates and kept in the dark at 37 °C for immunoreactivity. The signals were stopped by the addition of 50 μL stop solution. Signal intensity was analyzed by detecting absorbance at a wavelength of 450 nm using a microplate reader (Biotek, Winooski, VT, USA). The concentration of analyte in each sample was determined after comparison with its calibration curve. The anti-human SELENBP1 antibody (Catalog No.: MM-51436H1) and anti-human VCL antibody (Catalog No.: MM-51438H1) for ELISA purposes were purchased from Jiangsu Meimian Biotechnology (Yancheng, Jiangsu, China). The anti-human cTn-I antibody (Catalog No.: BP-E11199) and anti-human CK-MB antibody (Catalog No.: BP-E11107) for ELISA applications were purchased from Shanghai Boyun Biotechnology (Shanghai, China). As per the manufacturer’s instructions, cross-reactions with other non-specific analytes, and the influence of a spectrum of other biological substances and drugs, were negligible due to the use of specific monoclonal antibodies in this system.

### 4.15. Cells and Cell Culture

Human AC-16 cells were house bred and cultured in Dulbecco’s modified Eagle’s medium (DMEM) (Gibco, Los Angeles, CA, USA) with 10% fetal bovine serum (FBS) (Gibco). Human fetal lung fibroblast 1 (HFL1) cells were purchased from the Chinese Academy of Sciences Cell Bank (Shanghai, China) and were maintained in the Ham’s F-12K medium (Thermo Fisher Scientific) supplemented with 10% FBS. Human umbilical vein endothelial cells (HUVECs) and human aortic smooth muscle cells (HASMCs) were kindly provided by Prof. Dan Meng from the Department of Physiology and Pathophysiology, School of Basic Medical Sciences, Fudan University. For the HUVEC culture, endothelial cell growth supplements were added to an Endothelial Cell Medium (ECM) (ScienCell, Carlsbad, CA, USA) containing 5% FBS. HASMCs were cultured in a smooth muscle cell medium (ScienCell) with the supplement of 5% FBS and a smooth muscle cell growth supplement. All cells were incubated at 37 °C in a humidified atmosphere of 5% CO_2_ and 95% air and were grown to a confluence of approximately 70–80% before treatments. Cells were treated with 0.5 mM ACh for 3 min after the pre-2h replacement of the fresh serum-free medium.

### 4.16. Western Blot Analysis

As previously described [54], cell culture SNs were precipitated by the addition of an equal volume of methanol and a quarter of the volume for the chloroform, followed by a vortex and centrifugation for 10 min at 20,000× *g*. The upper phase was discarded, and 500 µL of methanol was added. The mixture was centrifuged for 10 min at 20,000× *g* after vortexing thoroughly, and the protein pellet was dried at room temperature. It was suspended in PBS, and an equal volume of 2× SDS (Beyotime Biotechnology, Nantong, China) was subsequently subjected to boil at 95 °C for 20 min. The cells in the culture dishes were lysed at 4 °C using a RIPA buffer (Beyotime) supplemented with PMSF (Beyotime) and then boiled with 2× SDS. Samples were loaded onto a 12% SDS-PAGE gel and transferred to the nitrocellulose membrane (Millipore, NY, USA) by the electrophoretic transfer system (BioRad, Hercules, CA, USA). After blocking in 5% BSA for 1 h, the membrane was incubated with a specific primary antibody with a 1:1000 dilution at 4 °C overnight. The bound antibody was visualized using HRP-conjugated secondary antibodies (Catalog No.: 7074 and 7076, Cell Signaling Technology, Danvers, MA, USA) and an ECL kit (Catalog No.: 34096, Thermo Fisher Scientific). The intensity was quantified using Image J v1.53 (National Institutes of Health, Bethesda, MD, USA) and normalized to intracellular GAPDH expression.

Detailed information on the primary antibodies used are as following: an Anti-Vinculin Rabbit monoclonal antibody (Catalog No.: 13901, Cell Signaling Technology); Anti-Selenium Binding Protein 1 antibody (Catalog No.: ab90135, abcam, Cambridge, UK); and Anti-GAPDH Mouse monoclonal antibody (Catalog No.: CW0100, CoWin Biosciences, Jiangsu, China).

### 4.17. Statistical Analysis

Data were expressed as the mean ± standard deviation for the continuous variables and as percentages for the categorical variables. Skewed values were presented as the median and interquartile range. We used Chi-square tests to compare the categorical variables. For continuous variables, comparisons between the two groups were analyzed using a Student’s *t*-test. Differences in the continuous variables among ≥3 groups were analyzed by one-way analysis of variance (ANOVA). If the data were not normally distributed, the Kruskal–Wallis test was employed. The unsupervised PCA between the basic information and protein expression was performed using R 4.0.5 tools (GUI 1.73 Catalina build). ROC analysis was conducted to quantify the diagnostic performance of serum proteins by assessing the sensitivity, specificity, and respective AUC with a 95% confidence interval (CI) using GraphPad Prism 8.2.0 statistical software (GraphPad, La Jolla, CA, USA) and MedCalc Version 20.009. For analyzing the association between the pre-diagnostic serum SELENBP1, the VCL levels, and future CAS risk, the odds ratios (ORs) and 95% CIs were computed using binary logistic regression models by R 4.0.5 tools (GUI 1.73 Catalina build). 

## 5. Patents

L. Li, X. Lin, Z. Liu, C. Xu, and B. Yu are inventors on a patent application (202110807976.X) submitted by the Fudan University that covers “Biomarkers identification for coronary artery spasm diagnosis and their applications”.

## Figures and Tables

**Table 1:** Baseline characteristics of collected clinical samples from the two groups of patients.

Categories	Total	CAS	Non-CAS	*p*-Value
	(*n* = 74)	(*n* = 25)	(*n* = 49)	
Men, *n* (%)	58 (78.4%)	16 (64.0%)	42 (85.7%)	0.032
Age (year)	65 (55–73)	63 (53–69)	68 (56–76)	0.076
Smoking, *n* (%)	18 (24.3%)	6 (24.0%)	12 (24.5%)	0.963
Drinking, *n* (%)	8 (10.8%)	1 (4.0%)	7 (14.3%)	0.253
Hypertension, *n* (%)	54 (73.0%)	17 (68.0%)	37 (75.5%)	0.401
DM, *n* (%)	27 (36.5%)	8 (32.0%)	19 (38.8%)	0.524
Fast blood glucose (mmol/L)	5.5 (4.8–7.0)	5.3 (4.7–5.6)	6.0 (4.9–8.5)	0.005
HbA1c (%)	5.8 (5.5–7.2)	5.7 (5.5–6.3)	5.9 (5.4–7.8)	0.072
Total cholesterol (mmol/L)	3.8 (3.0–4.5)	3.6 (3.0–4.2)	3.9 (3.0–4.7)	0.139
Total triglyceride (mmol/L)	1.2 (0.9–2.1)	1.1 (0.8–1.3)	1.7 (0.9–2.3)	0.116
LDLC (mmol/L)	2.0 (±0.7)	1.9 (±0.7)	2.0 (±0.8)	0.348
HDLC (mmol/L)	1.2 (±0.6)	1.2 (±0.5)	1.2 (±0.7)	0.665
hsCRP (mg/L)	2.45 (0.50–35.80)	0.400 (0.150–1.95)	7.20 (1.35–63.50)	<0.001
cTnT (ng/mL)	0.048 (0.009–0.209)	0.009 (0.007–0.015)	0.081 (0.033–0.427)	0.007
CK-MB (U/L)	16.0 (12.8–22.0)	14.0 (12.0–17.0)	18.0 (14.0–24.0)	0.007
CK-MM (U/L)	66.0 (41.8–116.3)	76.0 (50.0–113.5)	57.0 (27.5–149.8)	0.386
log10(proBNP) (pg/mL)	2.7 (2.1–3.2)	2.3 (1.8–2.7)	2.8 (2.3–3.3)	0.013
LVEF (%)	62 (52–66)	62 (56–67)	62 (50–66)	0.543
Hemoglobin (g/L)	128 (113–140)	127 (121–137)	129.0 (106–141)	0.504
Albumin (g/L)	40 (35–43)	41 (39–44)	38 (33–42)	0.025
Blood creatinine (µmol/L)	84 (70–113)	83 (58–104)	85 (72–136)	0.448
SELENBP1 (ng/L)	209.8 (161.0–272.2)	151.1 (143.9–163.6)	234.4 (208.5–302.9)	<0.001
VCL (ng/mL)	113.1 (90.3–136.0)	86.7 (77.8–96.6)	126.0 (110.5–165.7)	<0.001
β-blocker, *n* (%)	30 (40.5%)	10 (40.0%)	20 (40.8%)	0.946
CCB, *n* (%)	30 (40.5%)	9 (36.0%)	21 (42.9%)	0.570
eGFR (mL/min/1.73 m^2^)	80 (54–96)	80 (60–96)	79 (52–96)	0.357
urine acid, µmol/L	352.0 (291.5–411.0)	341.0 (291.5–406.0)	356.0 (291.0–417.0)	0.727

Data are expressed as mean ± SD, number (percentage), or median (interquartile range). CCB—calcium channel blocker; CK-MB—creatine kinase-MB; CK-MM—creatine kinase-MM; cTnT—cardiac troponins T; DM—diabetes mellitus; eGFR—estimated glomerular filtration rate; HbA1c—hemoglobin A1c; HDLC—high-density lipoprotein cholesterol; hsCRP—high-sensitivity C-reactive protein; LDLC—low-density lipoprotein cholesterol; LVEF—left ventricular ejection fraction; proBNP—pro-brain natriuretic peptide.

**Table 2:** Univariate and multivariate logistic regression analyses for predicting the incidence of CAS in clinical patients. The odds ratio represents the risk change per 1 unit increase in markers.

Variables	Univariate Analysis			Multivariate Analysis		
	Odds Ratio	95% CI for Odds Ratio	*p* Value	Odds Ratio	95% CI for Odds Ratio	*p* Value
Sex	3.375	1.076–10.588	0.037	1.744	0.118–25.806	0.686
Age	0.970	0.938–1.004	0.081	0.971	0.877–1.075	0.573
Smoking	0.974	0.316–3.000	0.963			
Drinking	0.250	0.029–2.156	0.207			
Hypertension	0.632	0.215–1.854	0.403			
DM	0.718	0.259–1.992	0.525			
β-blocker	0.967	0.362–2.581	0.946			
CCBs	0.750	0.278–2.026	0.570			
Blood glucose	0.727	0.542–0.975	0.034	1.006	0.571–1.774	0.983
HbA1c	0.628	0.365–1.081	0.093	0.736	0.395–1.370	0.333
Total cholesterol	0.646	0.362–1.155	0.141			
Total glyceride	0.688	0.418–1.134	0.142			
LDLC	0.699	0.334–1.465	0.343			
HDLC	1.216	0.510–2.902	0.659			
hs-CRP	0.965	0.935–0.996	0.026	0.974	0.910–1.042	0.436
cTnT	0.059	0.002–1.455	0.084	1.578	0.091–27.400	0.754
CK-MB	0.930	0.866–0.998	0.045	0.901	0.780–1.040	0.155
CK-MM	0.999	0.996–1.002	0.409			
log_10_(proBNP)	0.418	0.204–0.855	0.017	0.62	0.074–5.164	0.658
LVEF	1.015	0.968–1.065	0.538			
Hemoglobin	1.007	0.987–1.028	0.500			
Albumin	1.104	1.002–1.216	0.045	1.031	0.785–1.355	0.825
Blood creatinine	0.999	0.997–1.001	0.452			
SELENBP1	0.949	0.926–0.978	<0.001	0.962	0.930–0.994	0.022
VCL	0.906	0.863–0.950	<0.001	0.917	0.848–0.991	0.028
eGFR	1.008	0.991–1.025	0.353			
Urine acid	0.999	0.995–1.004	0.723			

Uni-variate analysis: significant if *p*-value < 0.10. Multivariate analysis: significant if *p*-value adjusted < 0.050. CI—confidence interval.

**Table 3:** Binary logistic regression analysis of serum SELENBP1 and VCL for CAS diagnosis in clinic. Odds ratio represents the risk change per 10 unit increase of markers.

Variables	Odds Ratio	95% CI for Odds Ratio	*p*-Value
SELENBP1	0.677	0.520–0.881	0.004
VCL	0.464	0.368–0.805	0.006

**Table 4:** Baseline characteristics of collected forensic samples from the two groups of decedents.

Categories	Non-Cardiac Death	CAS-Induced Sudden Death
Cases	11	12
Age, years	42.73 ± 13.36	52.42 ± 4.25
Gender (male: female)	6:5	12:0
Interval from attack to death, h	<1.50 ± 0.50	<2.58 ± 2.33
Cause of death	Traumatic death/drug intoxication	Sudden cardiac death
Potential death mechanism	Hemorrhagic shock/respiratory suppression	Heart failure/arrhythmia
CK-MB (ng/mL)	173.22 ± 139.75	334.41 ± 186.64 *
cTnI (ng/L)	703.36 ± 309.96	793.88 ± 361.17

* Denotes statistical significance.

**Table 5:** Binary logistic regression analysis of serum SELENBP1 and VCL for diagnosis of CAS-induced sudden death. Odds ratio represents the risk change per 10-unit increase in markers.

Variables	Odds Ratio	95% CI for Odds Ratio	*p*-Value
SELENBP1	0.283	0.104–0.775	0.014
VCL	0.506	0.265–0.968	0.040

## Data Availability

The label-free serum proteome raw data have been deposited to the ProteomeXchange Consortium (http://proteomecentral.proteomexchange.org) via the iProX partner repository with the dataset identifier PXD036383.

## Supplementary Materials

The following supporting information can be downloaded at: https://www.mdpi.com/article/10.3390/ijms232113266/s1.
