# Supplementary material for: Serum SELENBP1 and VCL Are Effective Biomarkers for Clinical and Forensic Diagnosis of Coronary Artery Spasm
Source: Int J Mol Sci. 2022 Oct 31;23(21):13266. doi: 10.3390/ijms232113266 (PMC9655542; doi:10.3390/ijms232113266)
Supplement: Supplementary file 1 [file ijms-23-13266-s001.zip › ijms-1959054-supplementary.pdf]

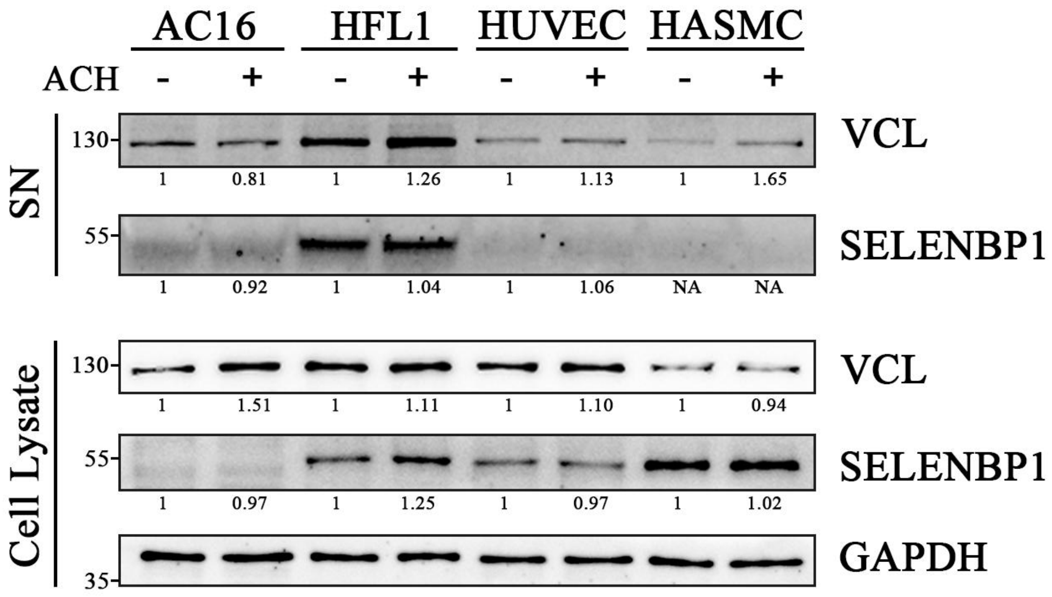

Figure S1. The contents of SELENBP1 and VCL in cell lysates and culture supernatants (SNs) of four major cardiotropic cell types, including human cardiomyocytes (AC-16), fibroblasts (HFL1), endothelial cells (HUVEC) and vascular smooth muscle cells (HASMC), were altered after the treatment of 0.5 mM acetylcholine (ACh). The levels were normalized to intracellular GAPDH expression and the values of four cell types without ACh stimulation were defined as 1. NA—not applicable.

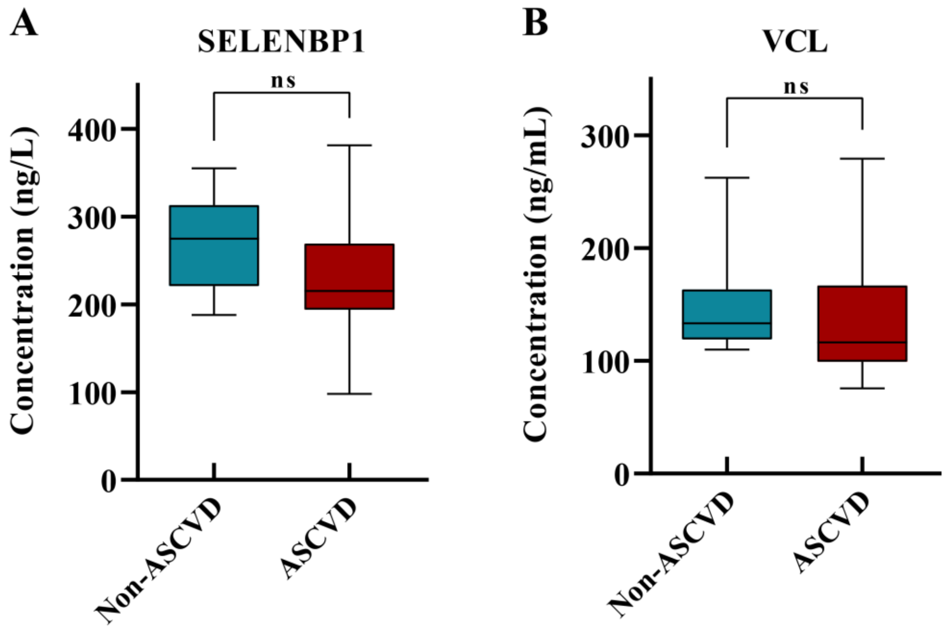

Figure S2. Serum levels of SELENBP1 and VCL in two subgroups of non-CAS group. (A) Serum levels of SELENBP1 in non-ASCVD (atherosclerotic cardiovascular diseases) and ASCVD patients. (B) Serum levels of VCL in non-ASCVD and ASCVD patients.

Table S1. Proteins identified by label-free proteome

Significantly differentially expressed (DE) proteins														
No.	**Protein**	**Protein Name**	**Proteins**	**Peptides**	**RazorUniquePeptides**	**Unique Peptides**	**LFQ intensity CAS-1**	**LFQ intensity CAS-2**	**LFQ intensity CAS-3**	**LFQ intensity Ctrl-1**	**LFQ intensity Ctrl-2**	**LFQ intensity Ctrl-3**	**CAS/Ctrl**	**p value**
1	G1U9S2	ALB	2	61	61	50	7.6E+11	6.48E+11	1.27E+12	2.28E+11	2.09E+11	1.96E+11	4.230	0.023
2	G1TFV7	LOC1000008[illegible]	2	20	3	3	2.63E+09	2.31E+09	4.74E+09	1.04E+09	1.33E+09	4.64E+08	3.411	0.047
3	P06912	RBP4	2	10	10	10	3.1E+09	1.8E+09	3.3E+09	7.56E+08	9.55E+08	6.95E+08	3.405	0.016
4	G1SS69	CFB	2	23	23	23	2.85E+09	2.16E+09	3.91E+09	9.07E+08	7.94E+08	9.28E+08	3.396	0.015
5	G8ZF28	HBA1	13	8	8	0	7.66E+09	4.73E+09	1.05E+10	2.99E+09	2.99E+09	1.73E+09	2.974	0.043
6	G1U5G9	F7	1	6	1	1	4.2E+08	6.74E+08	4.6E+08	1.82E+08	1.54E+08	1.98E+08	2.913	0.013
7	G1T4J9	RNASE4	1	5	5	5	1.66E+09	1.08E+09	1.09E+09	3.33E+08	3.8E+08	6.21E+08	2.866	0.018
8	G1TM88	SERPINA3	1	18	18	18	3.41E+09	1.96E+09	2.96E+09	1.53E+09	1.33E+09	1.24E+09	2.036	0.032
9	O77782		2	3	3	3	2.13E+09	2.11E+09	1.06E+09	2.56E+09	3.87E+09	4.34E+09	0.492	0.046
10	P29751	ACTB	1	15	15	2	4.44E+09	3.93E+09	2.58E+09	1.12E+10	8.92E+09	5.86E+09	0.421	0.039
11	G1SJ56	VCL	1	22	22	22	3.83E+08	2.72E+08	1.84E+08	7.33E+08	6.11E+08	7.3E+08	0.405	0.004
12	G1SPU6		3	14	2	2	1.39E+09	1.09E+09	6.36E+08	3.25E+09	2.95E+09	2.04E+09	0.378	0.016
13	G1SGW4	PSMB9	1	4	4	4	98403000	1.28E+08		3.28E+08	4.06E+08	3.08E+08	0.326	0.010
14	U3KP46	TUBB1	1	9	7	5	94105000	42326000	1.57E+08	5.62E+08	4.1E+08	2.41E+08	0.242	0.036
15	G1SL68	MYH9	6	35	35	35		1.05E+08	49896000	9.96E+08	7.56E+08	6.31E+08	0.098	0.014
16	A0A0C6G3[illegible]		1	7	3	1	1.29E+09	1.45E+09	1.16E+09	7.43E+08	7.98E+08	8.3E+08	1.643	0.005
17	G1SE06	PLA2G7	1	17	17	17	1.33E+09	1.26E+09	1.28E+09	1.41E+09	1.55E+09	1.53E+09	0.861	0.014
18	G1SHV9	PSMB3	1	6	6	6	7.82E+08	7.14E+08	4.97E+08	9.92E+08	9.85E+08	9.03E+08	0.692	0.031
19	G1SIK0	SERPINC1	2	40	40	40	1.13E+11	1.15E+11	1.06E+11	7.79E+10	8.27E+10	6.91E+10	1.455	0.002
20	G1SKW5		1	3	2	2	3.94E+08	4.21E+08	4.08E+08	5.19E+08	5.56E+08	5.3E+08	0.761	0.001
21	Q9XSC5	CLU	2	17	17	17	1.3E+10	1.87E+10	1.36E+10	3.08E+10	2.82E+10	2.01E+10	0.573	0.038
22	G1SQG6	SERPINA5	1	15	15	15	8.05E+09	6.43E+09	5.39E+09	4.85E+09	3.99E+09	3.78E+09	1.575	0.045
23	G1SU36	APOM	1	10	10	10	6.95E+09	6.19E+09	4.94E+09	8.35E+09	1.02E+10	9.37E+09	0.647	0.015
24	G1T0F8	HABP2	1	17	17	17	2.13E+09	3.29E+09	1.91E+09	4.58E+09	4.28E+09	3.73E+09	0.582	0.024
25	G1T1B8		1	19	19	19	1.42E+10	1.36E+10	1.32E+10	9.57E+09	1.03E+10	1.06E+10	1.346	0.001
26	G1T4X8	PSMB2	4	10	10	10	1.32E+09	1.37E+09	1.65E+09	1.86E+09	1.87E+09	1.68E+09	0.802	0.040
27	G1T763	PIGR	1	14	14	3	4.74E+08	6.89E+08	5.7E+08	7.63E+08	8.64E+08	8.43E+08	0.702	0.024

28	G1T7A2	C4BPA	1	33	33	30	2.66E+10	2.73E+10	2.12E+10	3.18E+10	4.1E+10	4.61E+10	0.631	0.033
29	G1THD3		1	2	2	2	4.6E+08	4.98E+08	7.2E+08	3.43E+08	3.25E+08	2.91E+08	1.749	0.044
30	G1TYH7	APMAP	1	15	14	14	5.61E+08	5.19E+08	5.83E+08	9.36E+08	8.06E+08	8.31E+08	0.646	0.002
31	G1TZR6		1	3	3	3	1.85E+08	1.67E+08	2E+08	1.44E+08	1.27E+08	1.29E+08	1.379	0.010
32	G1U242	ANGPTL6	2	3	3	3	95428000	96832000	91975000	65072000	80520000	58496000	1.393	0.016
33	G1U679		1	3	3	3		1.11E+09	1.06E+09	5E+08	6.95E+08	8.3E+08	1.610	0.046
34	G1U6C9		1	10	10	10	7.65E+09	7.67E+09	7.48E+09	4.11E+09	5.02E+09	5.56E+09	1.552	0.003
35	O19045	F10	2	12	12	12	1.76E+09	2.38E+09	2.33E+09	3.29E+09	2.93E+09	3.24E+09	0.685	0.012
36	G1U754	HRG	2	25	25	25	4.55E+10	3.64E+10	5.72E+10	2.68E+10	2.81E+10	2.75E+10	1.688	0.035
37	G1U9R4	APOB	5	209	209	208	3.37E+10	3.7E+10	3.33E+10	5.11E+10	5.92E+10	6.39E+10	0.597	0.004
38	P01686		1	2	1	0	8.01E+08	7.87E+08	8.11E+08	4.71E+08	5.6E+08	4.14E+08	1.660	0.002
39	P26203		2	4	4	4	7.11E+08	7.51E+08	7.06E+08	6.5E+08	5.31E+08	5.63E+08	1.243	0.021
40	P33047	APOC1	1	5	3	3	3.12E+09	4.85E+09	5.11E+09	1.77E+09	2.51E+09	2.54E+09	1.917	0.036
41	P55057	APOC4	1	5	5	5	7.06E+09	7.33E+09	9.93E+09	3.97E+09	4.79E+09	4.93E+09	1.777	0.021
42	Q9GLY5	ITIH3	3	29	29	29	5.72E+09	7.14E+09	6.27E+09	8.76E+09	8.4E+09	7.83E+09	0.766	0.017
43	A0A0A0MC	S100A9	2	5	5	5	2.48E+08	1.82E+08		2.63E+08	4.65E+08	8.19E+08	0.417	0.250
44	A0A0B4J1C		1	5	2	2	2.18E+09	1.96E+09	5.39E+09	1.92E+09	4.38E+09	1.77E+09	1.181	0.745
45	A0A0C6FQU		4	8	4	3	1.79E+09	3.36E+09	5.14E+09	2.25E+09	2.4E+09	2.09E+09	1.527	0.290
46	A0A0C6G5I		3	8	8	1	2.02E+10	2.24E+10	2.53E+10	2.71E+10	2.79E+10	1.76E+10	0.934	0.680
47	A0A0G2JH2	HSP90AA1	2	13	13	10	1.49E+08	1.53E+08	1.82E+08	9.27E+08	4.53E+08	1.43E+08	0.318	0.203
48	P07466		2	2	2	2	3.48E+08	95122000	3.11E+08	1.8E+08	4.14E+08	5.62E+08	0.651	0.379
49	A0A140TAV	HBB2	1	11	11	3	1.58E+10	7.29E+09	1.67E+10	6.4E+09	5.83E+09	3.89E+09	2.467	0.063
50	A0A1Y1B84		3	5	1	0	1.51E+08	1.31E+08	6.03E+08	4.58E+08	1.74E+08	69262000	1.262	0.767
51	A0A1Y1B9F		5	5	1	1	1E+09	5.87E+08	8.36E+08	1.86E+09	1.41E+09	9.66E+08	0.573	0.102
52	A0A1Y1B8E		1	5	1	1	1.96E+09	5.32E+09	3.02E+09	6.12E+09	3.68E+09	1.33E+09	0.926	0.880
53	A0A1Y1B9N		4	5	1	0	1.06E+09	3.8E+09	3.18E+09	9.31E+08	1.68E+09	1.61E+09	1.904	0.215
54	A0A1Y1BBC		1	6	2	0	1.29E+10	6.14E+09	6.28E+09	8.08E+09	9.69E+09	6.58E+09	1.041	0.897
55	A0A1Y1BBF		2	7	2	1	97439000	2.48E+08	6.12E+08	3.65E+08	3.29E+08	2.33E+08	1.033	0.952
56	A0A1Y1BDX		9	6	2	0	4.85E+09	4.45E+09	6.55E+09	5.74E+09	3.25E+09	3.64E+09	1.256	0.344
57	A0A1Y1BE3		3	6	1	0	4.98E+09	2.79E+09	3.77E+09	4.6E+09	6.03E+09	2.38E+09	0.887	0.711
58	A5HC55		1	17	1	1	1.92E+09	2.09E+09	5.31E+08	1.44E+09	1.13E+09	1.36E+09	1.160	0.699
59	A5HC69		3	6	6	6	3.45E+08	7.48E+08	5.84E+08	1.12E+09	7.85E+08	2.59E+08	0.776	0.590
60	B0ZYR1	SLC	2	2	2	2	31459000		34999000	44904000	23812000		0.967	0.926

61	B6S6L6	apoCI	1	6	6	4	2.43E+10	2.85E+10	3.35E+10	1.34E+10	1.66E+10	2.59E+10	1.545	0.092
62	B7NZD7	PSMB4	2	7	7	7	1.24E+09	1.32E+09	7.34E+08	9.16E+08	1.09E+09	1.18E+09	1.036	0.856
63	G1TX15	APOA5	2	8	8	8	2.64E+08	2.31E+08	66095000	24639000	48200000	1.4E+08	2.633	0.176
64	B7NZM0	APOA4	2	25	25	25	1.09E+11	1.23E+11	1.06E+11	9.76E+10	1.1E+11	1.3E+11	1.004	0.968
65	B7NZM1	RA_m003_j	2	35	35	0	3.21E+11	3.43E+11	3.78E+11	2.87E+11	3.26E+11	4.22E+11	1.007	0.955
66	G1SE95	FLNA	5	27	27	27		9493200	51758000	8.22E+08	4.5E+08	1.03E+08	0.067	0.210
67	B7NZR5	F8	3	15	15	15	3.51E+08	2.55E+08	3.23E+08	1.49E+08	1.81E+08	2.76E+08	1.534	0.087
68	P02057	HBB1	13	11	3	3	1.53E+09	2.77E+09	5.58E+09		4.08E+08	8.83E+08	5.106	0.187
69	D4P4R2		2	23	23	2	3.58E+09	1.16E+10	5.48E+09	1.56E+09	5.5E+09	1.18E+10	1.096	0.882
70	D5FIT0	LPL	1	6	6	6	2.78E+08	2.4E+08	1.44E+08	89358000	64983000		2.854	0.072
71	D5G340		1	14	14	8	3.08E+10	3.33E+10	1.76E+10	3.82E+10	3.09E+10	4.15E+10	0.740	0.173
72	F5XVB8	VWF	4	84	84	84	7.31E+09	5.51E+09	6.92E+09	1.14E+09	2.26E+09	5.32E+09	2.264	0.055
73	G1SCJ8	SERPING1	1	7	7	7	3.49E+08	2.28E+08	5.08E+08	4.32E+08	3.03E+08	3.56E+08	0.995	0.984
74	G1SCK5	SERPINF1	2	13	13	13	1.29E+09	1.88E+09	1.72E+09	9.68E+08	1.36E+09	2.37E+09	1.040	0.897
75	G1SD43	GPLD1	1	40	40	40	2.13E+10	3.07E+10	2.1E+10	2.29E+10	2.59E+10	2.93E+10	0.935	0.668
76	G1SDA8	PSMA1	1	14	14	14	2.3E+09	2.39E+09	1.73E+09	2.31E+09	2.25E+09	1.82E+09	1.007	0.957
77	G1SDD6	AGT	1	14	14	14	1.3E+09	8.57E+08	1.94E+09	5.56E+08	4.14E+08	5.79E+08	2.648	0.057
78	G1SDR2	MYL9	2	7	7	7	1.2E+08		1.51E+08	2.36E+08	1.36E+08	1.55E+08	0.772	0.402
79	G1SDY5	YWHAZ	3	13	13	5	3.25E+08	2.28E+08	4.74E+08	1.36E+09	1.15E+09	5.7E+08	0.333	0.050
80	G1SEB2	ANGPTL3	1	13	13	13	2.35E+08	3.52E+08	3.35E+08	2.42E+08	2.6E+08	3.21E+08	1.121	0.490
81	G1SEK8	FETUB	2	10	10	10	4.65E+09	4.22E+09	4.98E+09	3.07E+09	6.83E+09	1.29E+10	0.607	0.357
82	G1SEL1	CFHR5	1	16	16	16	6.66E+08	8.28E+08	5.96E+08	3.43E+08	6.62E+08	1.09E+09	0.997	0.994
83	G1SEL4	F13B	2	30	30	30	1.74E+10	1.73E+10	1.05E+10	1.26E+10	1.3E+10	1.7E+10	1.059	0.771
84	G1SF18	LOC1003557	3	10	10	1	3.45E+08	4.98E+08	1.57E+09	37473000	1.16E+08	3.18E+08	5.125	0.176
85	P62975		4	3	3	3	84408000		62762000	67394000	73486000		1.045	0.806
86	G1SFD7		1	1	1	1	53976000	62923000	71333000		18242000	44622000	1.996	0.076
87	G1SFR3	ADAMDEC1	1	7	7	7	1.4E+08	93614000		1.39E+08	71741000	89946000	1.166	0.629
88	G1SFU2	LIPC	2	6	6	6	1.58E+08	1.02E+08		1.15E+08	90555000		1.266	0.465
89	G1SGF8	C6	1	34	34	34	1.4E+10	1.38E+10	1.26E+10	1.52E+10	1.19E+10	9.04E+09	1.121	0.469
90	G1SGQ2	SPP2	1	6	6	6	2.53E+09	2.52E+09	2.89E+09	2.27E+09	2.48E+09	1.59E+09	1.254	0.144
91	G1SGU0	VNN1	1	8	8	8	6.01E+08	4.46E+08	4.36E+08	2.67E+08	3.21E+08	4.62E+08	1.411	0.143
92	G1SGV9	PSMB8	1	8	8	8	4.81E+08	4.37E+08	5.04E+08	3.49E+08	3.97E+08	4.98E+08	1.143	0.285
93	G1SH05	TUBB	4	11	11	2	1.24E+08	59805000	1.35E+08	5.35E+08	3.35E+08	1.22E+08	0.322	0.139

94	G1SH58	CPN1	2	13	13	13	1.52E+09	1.52E+09	1.44E+09	1.72E+09	2.52E+09	3.24E+09	0.599	0.086
95	G1SHH2	AFM	2	16	16	16	1.99E+09	2.2E+09	2.46E+09	1.37E+09	2.05E+09	2.12E+09	1.199	0.253
96	G1SHU0	SHBG	2	11	11	11	9.7E+08	7.96E+08	1.63E+09	6.79E+08	6.55E+08	4.55E+08	1.896	0.112
97	G1SIB7	F12	1	16	16	16	2.66E+09	2.73E+09	1.93E+09	2.23E+09	2.92E+09	3.01E+09	0.897	0.476
98	G1SJM1	APOH	1	14	14	14	4.36E+09	3.64E+09	7.84E+09	1.72E+09	2.77E+09	3.68E+09	1.938	0.145
99	G1U155	LOC100344	9	2	2	2	59323000	52454000	1.14E+08	42234000	34452000		1.962	0.240
100	G1SJX3	CP	3	44	44	44	9.84E+09	1.05E+10	1.23E+10	2.08E+10	1.55E+10	1.12E+10	0.688	0.161
101	G1SKM9	CFI	3	15	15	14	1.4E+09	1.05E+09	2.09E+09	5.42E+08	6.15E+08	8.13E+08	2.307	0.054
102	Q28680	CD14	2	5	5	5	50952000	87838000			62527000	1.26E+08	0.736	0.568
103	G1SLP6	ADAMTS13	1	27	27	27	8.09E+08	8.33E+08	7.02E+08	3.73E+08	5.96E+08	1.11E+09	1.126	0.715
104	G1SM64	ITIH2	3	21	21	21	3.62E+09	4.64E+09	4.3E+09	2.98E+09	3.43E+09	4.12E+09	1.192	0.205
105	G1SMZ3	MST1	1	16	16	16	7.8E+08	1.77E+09	1.18E+09	9.27E+08	1.48E+09	1.38E+09	0.984	0.953
106	G1SN00		4	24	24	8	3.49E+10	2.65E+10	2.32E+10	4.36E+10	4.16E+10	2.89E+10	0.741	0.163
107	G1SN96	PON1	1	16	1	1	4.46E+08	3.25E+08		2.95E+08	5.19E+08	5.07E+08	0.875	0.635
108	G1SND0	QSOX1	1	17	17	17	4.44E+08	7.88E+08	1.99E+08	9.57E+08	7.23E+08	5.55E+08	0.640	0.264
109	G1SNT3	CARD6	3	39	39	39	1.93E+10	1.78E+10	1.74E+10	2.31E+10	1.84E+10	1.25E+10	1.010	0.957
110	G1SP97	LUM	3	9	9	9	1.54E+09	1.53E+09	2.53E+09	1.21E+09	1.34E+09	1.29E+09	1.457	0.156
111	G1SPF9	C5	2	80	80	80	4.14E+10	4.26E+10	3.45E+10	4.47E+10	3.98E+10	3.16E+10	1.021	0.870
112	G1SPH7	CPSF2	1	1	1	1	1.47E+08	1.45E+08	3.28E+08	1.44E+08	2.55E+08	2.41E+08	0.967	0.925
113	G1SQ02	PRDX1	1	2	2	2	4.65E+08	3.21E+08	1.75E+08	7.19E+08	5.48E+08	3.67E+08	0.588	0.164
114	G1SQ70	A2M	1	70	70	64	4.4E+10	2.74E+10	9.39E+10	1.25E+10	1.06E+10	7.47E+09	5.416	0.088
115	G1SQU1	PSMB10	1	4	4	4	1.16E+08	1.48E+08	1.98E+08	1.42E+08	1.88E+08	1.62E+08	0.940	0.735
116	G1SQV9		3	62	62	62	3.56E+10	3.2E+10	4.43E+10	3.32E+10	3.74E+10	4.18E+10	0.996	0.975
117	G1SRA2	PLA2G2A	5	3	3	3	72008000	1.75E+08	2.42E+08	5.33E+08	2.77E+08	81532000	0.548	0.391
118	G1SS66	C2	1	21	21	21	1.44E+09	8.37E+08	2.56E+09	2.13E+09	1.51E+09	4.35E+08	1.186	0.739
119	G1SS91	LOC100356	2	96	96	85	1.95E+11	2.71E+11	1.71E+11	2.36E+11	2.55E+11	2.65E+11	0.841	0.273
120	G1SSN7	VIT	1	1	1	1	2.65E+08	5.58E+08	4.37E+08	5.96E+08	57173000	3.39E+08	1.270	0.641
121	G1ST02	QPCT	1	4	4	4	1.72E+08	1.25E+08	92329000	4.32E+08	2.61E+08	90147000	0.498	0.265
122	G1ST17	PGLYRP1	1	4	4	4	1.64E+08	53671000			1.2E+08	1.46E+08	0.819	0.712
123	G1TSY8	AMBP	3	13	13	13	1.24E+09	1.26E+09	2.16E+09	1.33E+09	1.74E+09	1.93E+09	0.930	0.756
124	G1STA7	PGLYRP2	1	7	7	7	2.23E+08	2.08E+08	3.13E+08	2.54E+08	2.45E+08	2.07E+08	1.053	0.745
125	P68135	ACTA1	8	12	4	4	5.97E+08	1.76E+08	4.57E+08	6.81E+08	6.74E+08	4.59E+08	0.678	0.247
126	P19134	TF	6	45	45	44	6.59E+10	5.14E+10	1.52E+11	1.55E+10	1.21E+10	6.95E+09	7.794	0.068

127	G1STJ4		2	56	56	21	8.1E+10	1.08E+11	7.3E+10	1E+11	1.1E+11	1.26E+11	0.777	0.124
128	G1SU47	RARRES2	1	4	4	4	3.91E+08	2.91E+08	2.58E+08	3.71E+08	2.89E+08	3.94E+08	0.893	0.504
129	G1SU71	PSMB1	1	8	8	8	1.79E+09	1.7E+09	1.48E+09	2.04E+09	2.12E+09	1.54E+09	0.871	0.297
130	G1SU82	GC	4	29	29	29	1.85E+10	1.72E+10	3.61E+10	2.14E+10	1.83E+10	1.19E+10	1.392	0.372
131	O97862	CST3	2	5	5	5	2.73E+08	2.73E+08	2.31E+08	1.52E+08	2.96E+08	2.25E+08	1.156	0.469
132	G1SVE2	IGFBP2	1	9	9	9	6.61E+08	3.98E+08	3.07E+08	2.39E+08	3E+08	6.08E+08	1.190	0.665
133	G1SVR4	COLEC11	1	4	4	4	1.22E+08	1.76E+08	2.08E+08	1.25E+08	1.3E+08	1.52E+08	1.242	0.280
134	G1SWF0	SERPIND1	3	18	18	18	3.66E+09	4.82E+09	3.65E+09	2.54E+09	6.49E+09	1.16E+10	0.587	0.345
135	G1SWF6	HP	2	16	16	16	3.54E+09	2.01E+09	6.69E+09	3.5E+09	2.62E+09	4.46E+08	1.862	0.317
136	G1SWI7	PSMA8	1	4	4	3	1.09E+09	1.04E+09	7.49E+08	1.88E+09	1.56E+09	1.18E+09	0.623	0.064
137	G1SWK8	PSMB7	1	3	3	3	4.35E+08	3.37E+08	2.53E+08	4.93E+08	2.81E+08	3.33E+08	0.927	0.760
138	G1SWS9	VIM	1	2	2	2	38752000	49675000	37757000	36189000	24771000	5494800	1.899	0.110
139	G1SWV4	THBS4	2	14	14	13	1.21E+08	2.79E+08	3.53E+08	37113000	1.65E+08	5.91E+08	0.949	0.944
140	G1SX17	TCN2	1	12	12	12	3.07E+08	2.54E+08	3.36E+08	2.16E+08	5.85E+08	1.6E+09	0.374	0.293
141	G1SXB5	ARHGAP11	1	1	1	1	1.03E+08	1.55E+08	1.41E+08	1.97E+08	84304000	1.42E+08	0.942	0.832
142	G1SXD0	PLA1A	1	8	8	8	1.76E+08	1.14E+08		1.83E+08	1.88E+08	1.44E+08	0.845	0.430
143	G1SXG6	S100A8	1	5	5	5	1.66E+08	1.77E+08		1.35E+08	3.79E+08	6.3E+08	0.450	0.338
144	G1SY36	TPM4	4	9	9	5	1.51E+08	69511000	86398000	1.96E+09	1.09E+09	3.04E+08	0.092	0.101
145	G1TNQ1		6	4	4	3	1.95E+10	1.44E+10	1.26E+10	5.92E+08	6.21E+09	1.31E+10	2.342	0.099
146	G1SYK4	LOC100346	1	63	63	63	6.98E+09	5.83E+09	4.32E+09	1.16E+10	8.37E+09	3.09E+09	0.742	0.489
147	G1SYM4	A1BG	1	13	13	13	3.82E+09	3.46E+09	9.42E+09	1.31E+09	1.09E+09	1.1E+09	4.772	0.085
148	G1SYV9	TLN1	2	50	50	50	12734000	10075000	17319000	3.62E+09	1.48E+09	4.77E+08	0.007	0.117
149	G1SYW6	ANTXR1	1	3	2	2	21237000	30900000	20203000	14144000	25447000	41965000	0.887	0.744
150	G1SZ14	PSMA3	1	9	9	9	1.64E+09	1.64E+09	1.08E+09	1.66E+09	9.23E+08	1.82E+09	0.990	0.968
151	G1T0J6	ITIH1	4	26	26	26	2.09E+09	2.6E+09	4.34E+09	2.22E+09	2.49E+09	3.37E+09	1.117	0.700
152	G1T088	SBSN	1	1	1	1	53617000		63182000		35843000	39909000	1.542	0.059
153	G1T093	SACS	1	2	2	2	7.67E+09	6.08E+09	1.14E+10	8.83E+09	5.86E+09	3.57E+09	1.378	0.353
154	G1T0Q0		1	5	4	4	3.34E+09	3.96E+09	3.27E+09	2.44E+09	2.6E+09	3.65E+09	1.216	0.226
155	G1T0U8	PCYOX1	1	11	11	11	9.74E+08	1.15E+09	8.9E+08	1.48E+09	1.31E+09	1.11E+09	0.771	0.087
156	G1T0W8	FGB	1	39	39	29	8.63E+10	7.21E+10	7E+10	5.52E+10	9.03E+10	1.14E+11	0.881	0.592
157	G1T0X2	FGA	2	45	45	8	8.77E+10	7.44E+10	7.15E+10	5.98E+10	8.82E+10	1.27E+11	0.849	0.529
158	G1T127	KLKB1	1	15	14	14	1.09E+09	7.77E+08	9.38E+08	6.03E+08	6.58E+08	9.78E+08	1.255	0.269
159	G1T155	SPON1	1	8	8	8	2.41E+08	1.88E+08		2.23E+08	1.08E+08		1.297	0.519

160	G1T1Z5	ECM1	1	24	24	24	6.18E+09	8.57E+09	7.06E+09	5.9E+09	8.16E+09	1.23E+10	0.828	0.491
161	G1T235	PSMB6	1	6	6	6	8.13E+08	6.13E+08	4.55E+08	1.01E+09	9.53E+08	8.44E+08	0.669	0.054
162	G1T2L1	PSMA2	1	8	8	8	1.45E+09	1.51E+09	8.33E+08	1.11E+09	1.17E+09	1.28E+09	1.066	0.740
163	P36233	SPARC	2	6	6	6		2.15E+08	43134000	1.77E+08	1.31E+08	75870000	1.008	0.989
164	G1T2R2	ITPR1	1	1	1	1	8.5E+08	1.39E+09	4.45E+08	5.21E+08	6.24E+08	2E+09	0.852	0.792
165	G1T3V2	HSPB1	2	3	3	3	16536000	86345000	41515000	32683000		14332000	2.048	0.433
166	G1T3V4	TLK1	1	1	1	1		73679000	89604000	85131000	42697000		1.277	0.516
167	G1T3X1	C9	1	32	3	3	1.39E+09	1.42E+09	1.09E+09	2.91E+09	2.75E+09	1.32E+09	0.557	0.116
168	G1T3Z1	MBL2	1	4	4	4	2.31E+09	2.14E+09	8.75E+08	1.85E+09	1.41E+09	1.59E+09	1.098	0.754
169	G1T4Q9	PSMB5	1	8	8	8	8.19E+08	5.74E+08	3.09E+08	9.39E+08	6.11E+08	6.37E+08	0.778	0.422
170	G1T4W4	FBLN5	1	11	11	11	6.84E+08	7.1E+08	6.06E+08	4.94E+08	4.9E+08	7.17E+08	1.176	0.286
171	G1T4Z1	LRP1	3	15	15	15	42248000	71414000		1.5E+08	96855000	1.05E+08	0.484	0.085
172	G1T519	PSMA4	1	10	10	10	1.31E+09	1.19E+09	1.48E+09	1.19E+09	1.21E+09	1.42E+09	1.040	0.667
173	G1T5K3	APOA2	1	4	4	4	1.52E+09	1.23E+09	2.07E+09	1.16E+09	1.02E+09	1.14E+09	1.450	0.120
174	G1T5S1	LBP	2	14	14	14	1.55E+10	1.66E+09	1.58E+09	1.35E+10	9.02E+09	1.31E+09	0.785	0.784
175	G1T670	SORT1	2	10	10	10	1.98E+09	1.84E+09	1.46E+09	2.31E+09	2.57E+09	1.74E+09	0.799	0.200
176	G1T6X7		5	7	1	0	1.65E+08	1.41E+08	8.62E+08	20282000	36020000	41527000	11.947	0.206
177	G1T739	APOF	1	6	6	6	1.58E+08	1.5E+08	2.34E+08	3.63E+08	1.77E+08	1.32E+08	0.807	0.598
178	G1T7G7	C1S	1	19	19	18	2.51E+09	2.43E+09	1.95E+09	2.87E+09	3.14E+09	2.69E+09	0.793	0.051
179	G1T7H9	C1R	1	21	21	21	3.23E+09	3.12E+09	2.15E+09	3.97E+09	3.66E+09	2.95E+09	0.804	0.204
180	G1T7U6	EFEMP1	1	17	17	17	2.05E+09	1.81E+09	1.57E+09	1.21E+09	1.75E+09	1.9E+09	1.117	0.493
181	G1T821	FGL1	1	16	16	16	1.42E+10	7.21E+09	5.49E+09	1.25E+10	1.04E+10	5.13E+09	0.957	0.912
182	G1T8T3	C1QC	1	5	5	5	1.28E+10	1.36E+10	5.09E+09	1.38E+10	1.56E+10	1.51E+10	0.707	0.192
183	G1T8V2	MFAP4	1	3	3	3	5.09E+08	9.53E+08	5.69E+08	1.64E+09	2E+09	1.99E+08	0.529	0.348
184	G1T9L4	CAMP	2	3	3	3	33373000	74952000	1.43E+08	1.68E+08	1.41E+08	1.72E+08	0.522	0.083
185	G1T9V4	PSMA6	1	13	13	13	2.11E+09	2.33E+09	1.79E+09	2.94E+09	2.51E+09	2.09E+09	0.826	0.210
186	Q28661	PROC	2	10	10	10	2.16E+09	3.22E+09	1.93E+09	2.49E+09	2.38E+09	3.05E+09	0.922	0.670
187	G1TAN8	AOC2	1	1	1	1	1.12E+08	60851000			43442000	56201000	1.733	0.299
188	G1TAQ7		2	5	5	5	45405000	55493000		52032000	42939000	57103000	0.995	0.973
189	G1TAR4	OIT3	1	13	13	13	4.26E+08	5.39E+08	4.11E+08	4.13E+08	4.09E+08	4.78E+08	1.059	0.613
190	G1TB36	F2	4	32	32	32	7.59E+10	8.81E+10	9.28E+10	7.46E+10	9.59E+10	9.1E+10	0.982	0.856
191	G1TBC1	HSP90B1	2	6	5	5	60593000	49041000			25514000	47124000	1.509	0.270
192	P23775	SERPINA6	2	8	8	8	7.88E+08	1.14E+09	1.63E+09	2.39E+08	6.32E+08	1.1E+09	1.807	0.203

193	G1TBS8	CFH	1	57	57	57	6.33E+10	5.59E+10	3.38E+10	6.81E+10	6.9E+10	6.31E+10	0.764	0.157
194	G1TN68	LOC1003549	2	5	5	5	94735000	75002000		64252000	61252000		1.352	0.157
195	P98136	C8A	2	23	23	23	7.01E+09	5.53E+09	2.92E+09	7.35E+09	5.46E+09	4E+09	0.919	0.784
196	G1TKL2	MYL6	3	4	4	4	1.48E+08	1.57E+08	1.09E+08	2.67E+08	2.09E+08	1E+08	0.720	0.352
197	G1TE43		1	2	2	2	89952000	2.31E+08			38965000	78386000	2.732	0.299
198	G1TET0	CPB2	2	14	14	14	1.96E+09	1.54E+09	1.58E+09	1.73E+09	1.65E+09	1.08E+09	1.137	0.452
199	G1TET2	LCP1	3	12	12	12	1.64E+08	1.58E+08	1.38E+08	70778000	1.13E+08	3E+08	0.953	0.919
200	G1TF67	CORO1A	2	4	4	4	27896000	18234000		23220000	29291000	24611000	0.897	0.583
201	G1TFU9	LGALS3BP	1	21	21	21	1.54E+10	3.15E+10	3.22E+10	2.03E+10	2.25E+10	2.73E+10	1.127	0.640
202	G1TFW8	LTF	1	29	28	28	2.17E+09	9.31E+08	6.27E+08	1.32E+09	1.39E+09	1.01E+09	1.000	0.999
203	G1TFX2	LOC100328[illegible]	2	21	2	2	3.57E+08	2.58E+08	7.53E+08	78214000	32283000	82169000	7.094	0.062
204	G1TGM2	SAA4	1	11	11	10	1.89E+10	4.11E+09	4.24E+09	3.15E+10	2.02E+10	5.12E+09	0.480	0.340
205	G1TGU6		1	1	1	1	63140000	89602000	1.96E+08		42135000	88733000	1.778	0.425
206	G1THZ6		57	16	4	3	2.33E+10	2.96E+10	3.63E+10	1.59E+10	1.62E+10	2.4E+10	1.589	0.074
207	G1TI71	UGP2	1	13	13	13	24326000	3.4E+08	69820000		2.1E+08	1E+09	0.239	0.247
208	G1TIU0		31	2	2	1	1.79E+08	2.28E+08	1.52E+08	1.5E+08	95557000	1.64E+08	1.368	0.176
209	G1TIY2		1	1	1	1	37682000	37147000	1.41E+08	54557000	55372000	40563000	1.436	0.566
210	G1TJG6		6	13	13	13	4.56E+08	4.28E+08	5.87E+08	1.16E+09	9.71E+08	5.61E+08	0.547	0.090
211	G1TJP4	CHI3L1	1	5	5	5	51462000	27809000		1.04E+08	55842000		0.497	0.272
212	P01692		3	1	1	1	38766000		77615000	36906000	26380000		1.839	0.318
213	G1TKC9	TGFBI	2	6	6	6	63287000	52900000		50026000		57156000	1.084	0.549
214	G1U693		2	2	2	2		1.2E+08	84568000	1.21E+08	1.04E+08	1.1E+08	0.914	0.560
215	G1TKP3		3	6	6	2	2.39E+10	2.49E+10	1.78E+10	7.55E+09	1.25E+10	1.93E+10	1.696	0.089
216	G1TKX3	FGG	1	27	27	27	5.98E+10	4.93E+10	3.76E+10	3.85E+10	5.52E+10	7.81E+10	0.854	0.560
217	G1TLA0		2	4	3	3	56672000	65961000	1.76E+08	95920000	2.3E+08		0.610	0.433
218	G1TLD3	NUCB1	1	3	3	3	26118000	44596000			41582000	43531000	0.831	0.519
219	P01693		2	1	1	1	1.19E+09	1.12E+09	91819000	7.36E+08	3.66E+08	2.62E+08	1.763	0.417
220	G1U1J4	LCAT	3	10	10	10	1.94E+09	2.5E+09	1.76E+09	2.27E+09	2.15E+09	2.33E+09	0.917	0.457
221	G1TN00		1	8	8	8	1.6E+09	2.49E+09	1.3E+09	2.02E+09	1.93E+09	2.27E+09	0.868	0.503
222	G1TN25	AZGP1	1	13	13	13	9.74E+08	1.08E+09	3.83E+09	2.34E+08	2.45E+08	2.8E+08	7.755	0.142
223	G1TN48		1	1	1	1	1.09E+08	1.3E+08	1.04E+08	1.58E+08	1.38E+08	1.21E+08	0.821	0.137
224	G1TN89	HSPG2	1	19	19	19	4.02E+08	3.52E+08	3.38E+08	3.14E+08	2.79E+08	3.87E+08	1.114	0.372
225	G1TNC5		1	1	1	1	4.01E+08	1.54E+08	2.38E+08	1.32E+08	1.28E+08	71420000	2.395	0.110

226	G1TNS3		2	1	1	1	94961000	2.13E+08	3.02E+08	70963000	81842000	1.24E+08	2.201	0.149
227	G1TNZ4	PROS1	3	13	13	13	6.32E+08	7.17E+08	7.46E+08	4.86E+08	6.4E+08	1.46E+09	0.809	0.618
228	G1TPR4		1	1	1	1	18864000	54203000		57737000	65088000	44009000	0.657	0.303
229	G1TPZ5		1	6	3	3	2.09E+08	1.61E+08		2.15E+08	2.07E+08	1.3E+08	1.004	0.985
230	G1TQR0	ACTN1	5	40	40	23	1.98E+08	1.63E+08	3.02E+08	2.38E+09	1.35E+09	6.31E+08	0.152	0.072
231	G1TR31		1	26	25	25	9.84E+08	2.38E+09	3.85E+09	93640000	4.71E+08	8.21E+08	5.208	0.085
232	G1TR82	TUBA4A	1	13	13	3	3.93E+08	3.29E+08	5.79E+08	1.38E+09	1.18E+09	5.57E+08	0.418	0.080
233	G1TRK9		4	7	7	7	4.06E+09	3.07E+09	4.17E+09	3.13E+09	2.14E+09	2.09E+09	1.537	0.054
234	G1TRN9		1	2	2	2	1.22E+08	1.13E+08	1.78E+08	1.74E+08	1.05E+08	59675000	1.218	0.561
235	G1TRW8	LCN2	1	11	11	11	3.67E+09	2.58E+09	4.01E+09	3.12E+09	4.45E+09	5.07E+09	0.812	0.333
236	G1TSN0	CHGA	1	3	3	3	45219000	36828000	64315000	55053000	66066000	73538000	0.752	0.174
237	G1TT86		1	4	3	1	7.24E+08	1.99E+09	3.22E+09	2.2E+09	2.07E+09	2.26E+09	0.909	0.797
238	G1TUX5	LOC1003527	1	3	3	3	2.55E+08	1.98E+08	6.18E+08	1.84E+08	1.52E+08	72743000	2.624	0.179
239	Q2TIL1		3	3	3	3	59724000		1.27E+08	54343000	34217000	43637000	2.114	0.156
240	G1TVN7		2	2	2	2	1.44E+09	3.71E+09	2.72E+09	3.58E+09	4.19E+09	1.89E+09	0.814	0.564
241	G1TVS4	HPX	3	21	21	21	8.63E+09	5.11E+09	1.53E+10	4.76E+09	3.46E+09	9.67E+08	3.162	0.107
242	G1TVU6	C1QTNF3	1	3	3	3	5.22E+08	4.94E+08	1.02E+08	2.96E+08	5.07E+08	5.46E+08	0.828	0.646
243	G1TVZ5		1	1	1	1	4.63E+08	1.45E+08	6.07E+08	5.48E+08	4.28E+08	2.99E+08	0.953	0.902
244	G1TWA7	FGA	4	38	1	1	1.85E+08	2.42E+08		2.29E+08	2.49E+08	4.55E+08	0.687	0.385
245	G1TXI3		1	1	1	1	2.21E+08	3.13E+08	3.55E+08	2.51E+08	2.15E+08	2.26E+08	1.284	0.185
246	G1TXW2		1	4	3	3	3.49E+09	1.87E+08	2.66E+08		1.06E+08	2.8E+08	6.811	0.483
247	G1TY29	ITIH4	1	36	36	3	6.51E+10	6.08E+10	3.67E+10	4.65E+10	5.27E+10	6.3E+10	1.003	0.988
248	G1TY57		1	4	4	4		3.45E+08	4.07E+08	6.04E+08	7.6E+08		0.552	0.068
249	G1TZI0		2	2	2	2	38036000	63986000		65134000	29632000	49324000	1.062	0.867
250	G1TYZ5		1	2	2	1	45432000	77317000	1.75E+08	1.51E+08	1.06E+08	37385000	1.014	0.980
251	G1TZA1	LOC1003524	1	5	5	5	2.18E+08	89433000	6.58E+08	3.35E+08	1.6E+08	29983000	1.839	0.490
252	G1TZC1	LOC1033507	2	17	17	17	4.3E+08	3.05E+08	1.9E+09	38968000	28136000	55355000	21.521	0.177
253	G1U0D7	COLEC10	1	4	4	4	1.35E+08	1.57E+08		1.1E+08	1.42E+08	1.26E+08	1.158	0.268
254	G1U128	MASP1	1	17	16	11	1.79E+09	1.25E+09	9.35E+08	1.59E+09	1.62E+09	1.13E+09	0.912	0.689
255	G1U2G9	MCP2	3	2	2	2	1.76E+09	4.46E+08	1.31E+09	5.85E+08	5.18E+08	4.81E+08	2.218	0.171
256	G1U2Q3	APOC2	1	2	2	2	6.41E+09	1.42E+10	1.18E+10	6.98E+09	7.77E+09	5.82E+09	1.578	0.171
257	G1U2V8	APOC3	1	4	4	4	1.64E+10	2.22E+10	2.43E+10	1.31E+10	1.87E+10	1.93E+10	1.229	0.271
258	G1U3C5	IGFBP5	1	5	5	5	50933000	57786000	1.12E+08	80728000	1.02E+08	69996000	0.874	0.650

259	G1U3U2	CLSTN1	1	11	11	11	5.37E+08	3.68E+08	1.83E+08	3.58E+08	3.39E+08	3.74E+08	1.015	0.961
260	G1U3Y6	ANTXR2	1	8	8	7	93832000	1.56E+08	1.11E+08	89017000	75280000	1.65E+08	1.099	0.763
261	G1U415	VTN	2	14	14	13	1.6E+10	1.55E+10	1.2E+10	2.29E+10	1.89E+10	1.38E+10	0.782	0.240
262	G1U466	TUBA1A	4	12	3	0		39531000	49339000	97439000	62999000	81120000	0.552	0.074
263	G1U4J2		2	3	3	2	25245000	1E+09	1.02E+09		2.92E+08	1.35E+09	0.834	0.830
264	G1U575	LOC1003533	1	1	1	1		18306000	1.27E+08	89146000	23236000		1.293	0.820
265	G1U5Y2		1	2	1	1		53869000	71574000	80812000	80694000	48315000	0.897	0.672
266	G1U612	LOC1003449	1	11	11	11	4.78E+10	5.35E+10	5.02E+10	5.51E+10	5.53E+10	6.32E+10	0.873	0.078
267	G1U6P5		1	12	5	5	2.72E+09	1.29E+09	8.21E+08	2.03E+09	1.58E+09	7.2E+08	1.115	0.822
268	G1U6S4	IHH	2	7	7	7	1.02E+08	1.16E+08	1.22E+08	1.23E+08	1.39E+08	1.56E+08	0.811	0.077
269	G1U6T0	F5	1	72	72	64	6.32E+10	5.03E+10	3.73E+10	7.62E+10	7.03E+10	4.8E+10	0.775	0.269
270	G1U7L4	HSPA5	5	14	14	12	6.25E+08	3.95E+08	4.36E+08	4.65E+08	4E+08	3.56E+08	1.193	0.368
271	G1U804		1	3	3	3	90312000	61268000	90813000	95796000	1.53E+08	99750000	0.696	0.165
272	G1U9Q9		2	48	48	48	4.74E+10	4.16E+10	2.6E+10	3.53E+10	6.75E+10	8.11E+10	0.625	0.201
273	G1U9R6	FN1	5	96	96	84	5.42E+10	5.9E+10	4.43E+10	4.47E+10	5.82E+10	7.53E+10	0.884	0.523
274	G1U9R8	GSN	3	32	32	32	1.72E+10	2.06E+10	1.74E+10	7.28E+09	2.5E+10	4.95E+10	0.674	0.509
275	G1U9S1	CFP	1	13	13	13	1.97E+09	2.94E+09	1.75E+09	1.76E+09	1.85E+09	3.39E+09	0.952	0.868
276	G1U9U2	F9	2	8	8	8	7.3E+08	7.93E+08	4.76E+08	8.24E+08	1.06E+09	9.35E+08	0.708	0.082
277	P01948		5	8	2	1	3.44E+09	1.81E+09	5.59E+09	7.44E+08	7.5E+08	1.01E+09	4.338	0.065
278	P01684		1	1	1	1	1.51E+08	2.23E+08	3.28E+08	2.2E+08	1.8E+08	93005000	1.425	0.334
279	P01685		2	2	2	1	1.78E+08	1.94E+08	2.55E+08	3.89E+08	2.88E+08	1E+08	0.808	0.601
280	P01687		2	2	1	0	4.82E+08	6.28E+08		1.01E+09	8.6E+08	6.35E+08	0.664	0.161
281	P01697		1	3	3	1	2.81E+09	4.15E+09	6.73E+09	1.61E+09	1.78E+09	2.22E+09	2.441	0.082
282	P01870		2	18	18	6	1.03E+11	1.43E+11	1.91E+11	8.67E+10	9.65E+10	9.79E+10	1.553	0.112
283	P01879		1	14	14	7	4.84E+09	4.1E+09	2.47E+09	4.07E+09	3.32E+09	2.74E+09	1.126	0.622
284	P02742	CRP	1	10	10	2	1.3E+10	1.37E+09	1.59E+09	2.1E+10	1.62E+10	1.16E+09	0.417	0.353
285	P03988		5	14	14	1	3.34E+10	4.72E+10	4.23E+10	3.01E+10	3.42E+10	4.97E+10	1.078	0.702
286	P07467		1	2	2	2	5.67E+08	1.33E+08	1.58E+08	1.03E+08	2.57E+08	2.09E+08	1.509	0.549
287	P07489	TTR	2	7	7	7	1.36E+10	1.71E+10	2.58E+10	1.08E+10	8.57E+09	7.1E+09	2.132	0.058
288	P11974	PKM	4	9	9	9	89847000	64342000	59454000	1.61E+08	61660000	42034000	0.808	0.679
289	P14461	FGA	1	1	1	1	1.55E+08	1.37E+08	2.44E+08	1.54E+08	8.41E+08	1.8E+09	0.192	0.191
290	P18287	APOE	1	13	7	5	2.07E+10	2.09E+10	3.12E+10	1.59E+10	1.3E+10	1.91E+10	1.515	0.101
291	P22000	SAA2	1	10	1	0	2.07E+09	1.44E+08		4.38E+09	4.74E+09	1.69E+08	0.358	0.399

292	P22687	CETP	1	22	1	1		6.26E+08	2.74E+08	39822000	3.41E+08	3.33E+08	1.891	0.331
293	P23108	JCHAIN	2	6	6	6	4.37E+09	4.71E+09	3.77E+09	5.25E+09	4.58E+09	4.18E+09	0.917	0.404
294	P25227	ORM1	1	2	2	2	31031000	21800000	1.6E+08	61843000	33689000		1.484	0.719
295	P27170	PON1	1	18	18	3	6.1E+10	7.64E+10	7.32E+10	4.75E+10	6.44E+10	8.63E+10	1.062	0.752
296	P30801	S100A6	1	2	2	2	1.37E+08	79983000	49783000	61512000	59790000	79087000	1.330	0.448
297	P30947	HSP90AB1	4	8	5	4	1.17E+08	1.42E+08	1.17E+08	1.81E+08	1.59E+08	1.34E+08	0.793	0.110
298	P31347	ANG	2	3	3	3	4.69E+08	3.87E+08	1.25E+08	2.45E+08	3.04E+08	3.8E+08	1.057	0.881
299	P37153	APOD	2	5	5	5	1.66E+10	1.58E+10	1.5E+10	1.06E+10	1.38E+10	2.02E+10	1.064	0.755
300	P41975	SOD3	1	8	8	8	6.13E+08	5.17E+08	9.3E+08	3.41E+08	4.68E+08	4.92E+08	1.583	0.131
301	P48747	C9	1	33	33	4	5.18E+10	6.33E+10	4.47E+10	6.45E+10	6.27E+10	5.48E+10	0.878	0.297
302	P83470	PF4	1	2	1	1		1.25E+08	4.88E+08	1.42E+08	7.43E+08	94309000	0.938	0.950
303	P98137	C8B	2	25	25	25	8.23E+09	8.49E+09	6.07E+09	1.38E+10	1.16E+10	6.87E+09	0.707	0.221
304	P98139	F7	1	6	6	1	98715000	1.36E+08	1.02E+08	1.4E+08	2.79E+08	3.11E+08	0.461	0.072
305	Q07298	LOC100008	1	21	21	1	1.56E+10	1.61E+10	3.27E+10	6.2E+09	7.5E+09	5.76E+09	3.308	0.057
306	Q28665	LOC100008	3	21	2	2	2.07E+08	1.86E+08	7.27E+08	61989000	72249000	61161000	5.733	0.156
307	Q28679	C8G	1	8	8	8	5.13E+09	4.38E+09	4.91E+09	7.89E+09	5.7E+09	3.91E+09	0.824	0.429
308	Q29514	LOC100009	1	10	9	1	2.63E+10	8.84E+08	1.47E+09	6.68E+10	5.43E+10	1.94E+09	0.232	0.218
309	Q2PPJ8	apoA-I	1	35	2	0	1.54E+10	1.38E+08	2.08E+08	2.91E+10	2.03E+10	6.12E+08	0.316	0.311
310	Q45GR2	SERPINF2	1	19	19	19	4.8E+09	6.43E+09	7.54E+09	6.01E+09	4.89E+09	3.01E+09	1.349	0.244
311	Q5VI85		2	7	7	6	6.18E+09	9.71E+09	1.44E+10	2.03E+10	1.93E+10	4.7E+09	0.682	0.447
312	Q6B736		1	2	1	1	46043000	14002000	16846000	27477000	23651000		1.003	0.996
313	Q7M322		1	1	1	1	8.56E+08	6.32E+08		2.44E+08	4.44E+08	9.47E+08	1.365	0.532
314	Q8HYV6	masp-1/3	1	8	3	3	68614000	61153000	77297000	85358000	91455000	68053000	0.846	0.209
315	Q95JG0	PLTP	3	14	14	14	3.73E+09	4.98E+09	3.84E+09	2.66E+09	4.06E+09	4.65E+09	1.105	0.607
316	Q95ME7		2	22	22	22	1.46E+09	1.62E+09	1.07E+09	1.21E+09	1.64E+09	2.1E+09	0.840	0.437
317	Q9BGN0	PON3	1	16	16	16	5.7E+09	6.56E+09	4.27E+09	6.62E+09	6.84E+09	6.5E+09	0.828	0.164
318	Q9GLM9		1	3	1	1	32911000	36467000	63491000	65546000	58196000	58534000	0.729	0.173
319	Q9GLY4	ITI-HC4	1	35	2	2	1.75E+09	1.25E+09	7.67E+08	1.65E+09	1.98E+09	1.2E+09	0.781	0.387
320	Q9XSG0		1	13	1	1	2.01E+08	4.57E+08	3.36E+08	3.37E+08	3.77E+08	3.28E+08	0.953	0.840
321	U3KMI4	RAP1A	2	2	2	2	49021000	46459000	87347000	1.22E+08	1E+08	38898000	0.699	0.406
322	U3KMB5	C1QA	1	5	5	5	4.42E+09	4.71E+09	1.64E+09	4.15E+09	6.23E+09	7.22E+09	0.612	0.163
323	U3KMC5		1	3	3	3	2.15E+08	1.79E+08	3.02E+08	1.22E+08	61956000	1.75E+08	1.941	0.084
324	U3KMR2	AHSG	3	11	11	11	1.88E+10	1.84E+10	3.45E+10	1.22E+10	1.71E+10	2.97E+10	1.216	0.598

325	U3KPI5	CPN2	1	17	17	17	3.18E+09	3.41E+09	3.56E+09	4.02E+09	5.78E+09	7.45E+09	0.589	0.076
**Specific proteins**														
326	A0A1Y1B8E		1	6	1	0	2.76E+08	2.34E+08	2.89E+08				N/A	N/A
327	A0A1Y1B8Z		6	7	1	1		9467400	1.23E+08				N/A	N/A
328	P68105	EEF1A1	4	1	1	1	40862000		16307000				N/A	N/A
329	G1SMC0	CRP	1	9	1	1	2.15E+08	30059000					N/A	N/A
330	G1SQE6	SPARCL1	1	1	1	1	10235000	8940000					N/A	N/A
331	P60990	PIP	2	2	2	2		18147000	67569000				N/A	N/A
332	G1SR31		1	4	4	4	75876000	55860000	1.33E+08				N/A	N/A
333	G1T6G3		1	1	1	1	86710000		56588000				N/A	N/A
334	G1T7R2	YWHAE	2	7	4	4	42763000	1.13E+08					N/A	N/A
335	G1TC91	NELL2	1	2	2	2		46024000	35405000				N/A	N/A
336	G1TCW1	TFRC	1	3	3	3	53922000	23669000	59977000				N/A	N/A
337	G1TIZ7	ZSWIM1	1	1	1	1	41111000		1.05E+08				N/A	N/A
338	G1TW85		1	2	2	2	1.49E+08	1.54E+08	1.97E+08				N/A	N/A
339	Q6Q7K2		1	2	2	2	39068000	28694000					N/A	N/A
340	A0A1Y1B8E		2	6	1	1				81295000	66877000		N/A	N/A
341	B7NZB9	CTGF	3	6	6	6				3.94E+08	2.64E+08		N/A	N/A
342	G1THV1	OR51G1	2	1	1	1				1.88E+08	1.05E+08	99328000	N/A	N/A
343	G1SKI1	EZR	4	1	1	1				18260000	13511000	14717000	N/A	N/A
344	G1SIB0	LMAN2	1	1	1	1				35816000	27027000		N/A	N/A
345	G1SIE3	IGFBP4	1	3	3	3				1.81E+08	1.75E+08	74042000	N/A	N/A
346	G1SIL2	MINPP1	1	2	2	2				31788000	29065000	32917000	N/A	N/A
347	G1SJ23	CAPN1	1	3	3	3				20709000	19396000	22424000	N/A	N/A
348	G1SR03	VCP	1	4	4	4				89346000	58104000		N/A	N/A
349	G1SSK9	LOC100349	1	4	2	2				35784000	33288000		N/A	N/A
350	G1SSP5	TREML1	1	1	1	1				32689000	24087000		N/A	N/A
351	G1SZD6	YWHAQ	2	8	3	3				37576000	19806000		N/A	N/A
352	G1T1Q2	CALM1	2	3	3	3				1.05E+08	57976000		N/A	N/A
353	G1T5L0	IGHM	2	12	1	1				5774900	4284900	2463500	N/A	N/A
354	G1T8P4	RPLP1	1	1	1	1				9420800	9124700		N/A	N/A
355	G1TC37	EIF5A2	2	1	1	1				54135000	11730000		N/A	N/A
356	G1TGX7		1	3	1	1				1.04E+08	83923000		N/A	N/A

357	G1THE8		1	2	2	2				1.45E+08	1.09E+08		N/A	N/A
358	G1THQ5	ST6GAL1	1	3	3	3					16060000	33437000	N/A	N/A
359	G1TJU8		2	1	1	1				58855000		69373000	N/A	N/A
360	G1TKC8		1	2	2	2				42965000	11299000		N/A	N/A
361	G1U640		4	1	1	1				24613000	32978000		N/A	N/A
362	G1TMV1	DSTN	2	3	3	3				1.27E+08	70007000	56147000	N/A	N/A
363	G1TN86	CUTA	1	1	1	1					17895000	35423000	N/A	N/A
364	G1TNI5		1	3	1	0				18853000	37361000		N/A	N/A
365	G1TP66	LOC100350[illegible]	13	3	3	3					6026700	42246000	N/A	N/A
366	G1TT06	SELENBP1	2	4	4	4				54676000	36972000		N/A	N/A
367	G1TVD5		1	8	1	1				1.04E+08	1.21E+08		N/A	N/A
368	G1TW43	TNC	2	8	8	8				92004000	1.03E+08		N/A	N/A
369	G1TWM1		3	3	1	0					53914000	34400000	N/A	N/A
370	G1U6B2	ALAD	1	3	3	3				1.68E+08	1.71E+08		N/A	N/A
371	G1U7Q2		1	3	2	1					28730000	29658000	N/A	N/A
372	O19048	PCBP1	1	2	2	2				50143000	30537000		N/A	N/A
373	P01827		1	1	1	1				34670000	17952000		N/A	N/A
374	P01832	PIGR	1	14	3	3				1.49E+08	99773000		N/A	N/A
375	P09809	APOA1	1	33	1	0				2.38E+09	1.67E+09		N/A	N/A
376	U3KLX7	CUBN	1	4	4	4				36498000	29611000	23300000	N/A	N/A
377	U3KM01		1	2	1	1				34209000	26153000		N/A	N/A

N/A, not applicable

Table S2. GO enrichment analysis

GO_ID	Description	Term	Test	Ref	TestAll	RefAll	Test_per	Ref_per	P value	richFactor
GO:0032984	protein-containing complex disassembly	BP	3	4	67	483	0.044776	0.008282	0.00924	0.75
GO:0010628	positive regulation of gene expression	BP	7	19	67	483	0.104478	0.039337	0.00928	0.368421
GO:0042802	identical protein binding	MF	10	34	67	483	0.149254	0.070393	0.01131	0.294118
GO:0043228	non-membrane-bounded organelle	CC	11	41	67	483	0.164179	0.084886	0.016422	0.268293
GO:0043232	intracellular non-membrane-bounded organelle	CC	11	41	67	483	0.164179	0.084886	0.016422	0.268293
GO:0005905	clathrin-coated pit	CC	2	2	67	483	0.029851	0.004141	0.018994	1
GO:0043531	ADP binding	MF	2	2	67	483	0.029851	0.004141	0.018994	1
GO:0043243	positive regulation of protein complex disassembly	BP	2	2	67	483	0.029851	0.004141	0.018994	1
GO:0016887	ATPase activity	MF	2	2	67	483	0.029851	0.004141	0.018994	1
GO:0005903	brush border	CC	2	2	67	483	0.029851	0.004141	0.018994	1
GO:0098862	cluster of actin-based cell projections	CC	2	2	67	483	0.029851	0.004141	0.018994	1
GO:0006414	translational elongation	BP	2	2	67	483	0.029851	0.004141	0.018994	1
GO:0003676	nucleic acid binding	MF	6	17	67	483	0.089552	0.035197	0.020287	0.352941
GO:0044877	protein-containing complex binding	MF	8	28	67	483	0.119403	0.057971	0.028156	0.285714
GO:0010604	positive regulation of macromolecule metabolic process	BP	9	34	67	483	0.134328	0.070393	0.032721	0.264706
GO:0098590	plasma membrane region	CC	3	6	67	483	0.044776	0.012422	0.037497	0.5
GO:0043112	receptor metabolic process	BP	2	3	67	483	0.029851	0.006211	0.051849	0.666667
GO:0022612	gland morphogenesis	BP	2	3	67	483	0.029851	0.006211	0.051849	0.666667
GO:0008135	translation factor activity, RNA binding	MF	2	3	67	483	0.029851	0.006211	0.051849	0.666667
GO:0019079	viral genome replication	BP	2	3	67	483	0.029851	0.006211	0.051849	0.666667
GO:0051014	actin filament severing	BP	2	3	67	483	0.029851	0.006211	0.051849	0.666667
GO:0043244	regulation of protein complex disassembly	BP	2	3	67	483	0.029851	0.006211	0.051849	0.666667
GO:0003746	translation elongation factor activity	MF	2	3	67	483	0.029851	0.006211	0.051849	0.666667
GO:0051640	organelle localization	BP	2	3	67	483	0.029851	0.006211	0.051849	0.666667
GO:0043624	cellular protein complex disassembly	BP	2	3	67	483	0.029851	0.006211	0.051849	0.666667
GO:0015629	actin cytoskeleton	CC	4	11	67	483	0.059701	0.022774	0.05231	0.363636
GO:0003723	RNA binding	MF	4	11	67	483	0.059701	0.022774	0.05231	0.363636
GO:0009893	positive regulation of metabolic process	BP	9	37	67	483	0.134328	0.076605	0.054609	0.243243
GO:0010468	regulation of gene expression	BP	9	37	67	483	0.134328	0.076605	0.054609	0.243243
GO:0043230	extracellular organelle	CC	3	7	67	483	0.044776	0.014493	0.059177	0.428571

GO:1903561	extracellular vesicle	CC	3	7	67	483	0.044776	0.014493	0.059177	0.428571
GO:0070062	extracellular exosome	CC	3	7	67	483	0.044776	0.014493	0.059177	0.428571
GO:0005856	cytoskeleton	CC	7	27	67	483	0.104478	0.055901	0.06482	0.259259
GO:0006897	endocytosis	BP	4	12	67	483	0.059701	0.024845	0.070423	0.333333
GO:0042803	protein homodimerization activity	MF	4	12	67	483	0.059701	0.024845	0.070423	0.333333
GO:0098657	import into cell	BP	4	12	67	483	0.059701	0.024845	0.070423	0.333333
GO:1901566	organonitrogen compound biosynthetic process	BP	5	17	67	483	0.074627	0.035197	0.071783	0.294118
GO:0051130	positive regulation of cellular component organization	BP	5	17	67	483	0.074627	0.035197	0.071783	0.294118
GO:0034645	cellular macromolecule biosynthetic process	BP	7	28	67	483	0.104478	0.057971	0.077041	0.25
GO:0044271	cellular nitrogen compound biosynthetic process	BP	7	28	67	483	0.104478	0.057971	0.077041	0.25
GO:0005829	cytosol	CC	8	34	67	483	0.119403	0.070393	0.08184	0.235294
GO:0022411	cellular component disassembly	BP	3	8	67	483	0.044776	0.016563	0.085448	0.375
GO:0016192	vesicle-mediated transport	BP	5	18	67	483	0.074627	0.037267	0.088955	0.277778
GO:0017111	nucleoside-triphosphatase activity	MF	4	13	67	483	0.059701	0.026915	0.091344	0.307692
GO:0019058	viral life cycle	BP	2	4	67	483	0.029851	0.008282	0.094458	0.5
GO:0030099	myeloid cell differentiation	BP	2	4	67	483	0.029851	0.008282	0.094458	0.5
GO:0002573	myeloid leukocyte differentiation	BP	2	4	67	483	0.029851	0.008282	0.094458	0.5
GO:0006259	DNA metabolic process	BP	2	4	67	483	0.029851	0.008282	0.094458	0.5
GO:1903050	regulation of proteolysis involved in cellular protein catabolic proc	BP	2	4	67	483	0.029851	0.008282	0.094458	0.5
GO:0061136	regulation of proteasomal protein catabolic process	BP	2	4	67	483	0.029851	0.008282	0.094458	0.5
GO:1901137	carbohydrate derivative biosynthetic process	BP	2	4	67	483	0.029851	0.008282	0.094458	0.5
GO:0060255	regulation of macromolecule metabolic process	BP	12	59	67	483	0.179104	0.122153	0.094876	0.20339
GO:0019222	regulation of metabolic process	BP	13	66	67	483	0.19403	0.136646	0.10286	0.19697
GO:0009059	macromolecule biosynthetic process	BP	7	30	67	483	0.104478	0.062112	0.10532	0.233333
GO:0031325	positive regulation of cellular metabolic process	BP	7	30	67	483	0.104478	0.062112	0.10532	0.233333
GO:0044249	cellular biosynthetic process	BP	8	36	67	483	0.119403	0.074534	0.108201	0.222222
GO:0016462	pyrophosphatase activity	MF	4	14	67	483	0.059701	0.028986	0.114895	0.285714
GO:0016818	hydrolase activity, acting on acid anhydrides, in phosphorus-contai	MF	4	14	67	483	0.059701	0.028986	0.114895	0.285714
GO:0016817	hydrolase activity, acting on acid anhydrides	MF	4	14	67	483	0.059701	0.028986	0.114895	0.285714
GO:0051015	actin filament binding	MF	3	9	67	483	0.044776	0.018634	0.115756	0.333333
GO:1901363	heterocyclic compound binding	MF	11	55	67	483	0.164179	0.113872	0.119317	0.2
GO:0031323	regulation of cellular metabolic process	BP	11	55	67	483	0.164179	0.113872	0.119317	0.2

GO:0010467	gene expression	BP	9	43	67	483	0.134328	0.089027	0.122843	0.209302
GO:1901576	organic substance biosynthetic process	BP	8	37	67	483	0.119403	0.076605	0.122985	0.216216
GO:0016020	membrane	CC	14	75	67	483	0.208955	0.15528	0.131383	0.186667
GO:0034641	cellular nitrogen compound metabolic process	BP	7	32	67	483	0.104478	0.066253	0.138575	0.21875
GO:0070227	lymphocyte apoptotic process	BP	1	1	67	483	0.014925	0.00207	0.138716	1
GO:1900371	regulation of purine nucleotide biosynthetic process	BP	1	1	67	483	0.014925	0.00207	0.138716	1
GO:1903007	positive regulation of Lys63-specific deubiquitinase activity	BP	1	1	67	483	0.014925	0.00207	0.138716	1
GO:0097513	myosin II filament	CC	1	1	67	483	0.014925	0.00207	0.138716	1
GO:0019985	translesion synthesis	BP	1	1	67	483	0.014925	0.00207	0.138716	1
GO:0018279	protein N-linked glycosylation via asparagine	BP	1	1	67	483	0.014925	0.00207	0.138716	1
GO:0002891	positive regulation of immunoglobulin mediated immune response	BP	1	1	67	483	0.014925	0.00207	0.138716	1
GO:0046850	regulation of bone remodeling	BP	1	1	67	483	0.014925	0.00207	0.138716	1
GO:0032982	myosin filament	CC	1	1	67	483	0.014925	0.00207	0.138716	1
GO:0001848	complement binding	MF	1	1	67	483	0.014925	0.00207	0.138716	1
GO:0072512	trivalent inorganic cation transport	BP	1	1	67	483	0.014925	0.00207	0.138716	1
GO:0034632	retinol transmembrane transporter activity	MF	1	1	67	483	0.014925	0.00207	0.138716	1
GO:0006779	porphyrin-containing compound biosynthetic process	BP	1	1	67	483	0.014925	0.00207	0.138716	1
GO:1903919	negative regulation of actin filament severing	BP	1	1	67	483	0.014925	0.00207	0.138716	1
GO:0043467	regulation of generation of precursor metabolites and energy	BP	1	1	67	483	0.014925	0.00207	0.138716	1
GO:0003697	single-stranded DNA binding	MF	1	1	67	483	0.014925	0.00207	0.138716	1
GO:0070228	regulation of lymphocyte apoptotic process	BP	1	1	67	483	0.014925	0.00207	0.138716	1
GO:0045905	positive regulation of translational termination	BP	1	1	67	483	0.014925	0.00207	0.138716	1
GO:0006415	translational termination	BP	1	1	67	483	0.014925	0.00207	0.138716	1
GO:0060740	prostate gland epithelium morphogenesis	BP	1	1	67	483	0.014925	0.00207	0.138716	1
GO:0006449	regulation of translational termination	BP	1	1	67	483	0.014925	0.00207	0.138716	1
GO:1903793	positive regulation of anion transport	BP	1	1	67	483	0.014925	0.00207	0.138716	1
GO:0003730	mRNA 3'-UTR binding	MF	1	1	67	483	0.014925	0.00207	0.138716	1
GO:0050871	positive regulation of B cell activation	BP	1	1	67	483	0.014925	0.00207	0.138716	1
GO:0010888	negative regulation of lipid storage	BP	1	1	67	483	0.014925	0.00207	0.138716	1
GO:1903918	regulation of actin filament severing	BP	1	1	67	483	0.014925	0.00207	0.138716	1
GO:0051659	maintenance of mitochondrion location	BP	1	1	67	483	0.014925	0.00207	0.138716	1
GO:0031254	cell trailing edge	CC	1	1	67	483	0.014925	0.00207	0.138716	1

GO:0045838	positive regulation of membrane potential	BP	1	1	67	483	0.014925	0.00207	0.138716	1
GO:0030888	regulation of B cell proliferation	BP	1	1	67	483	0.014925	0.00207	0.138716	1
GO:0033572	transferrin transport	BP	1	1	67	483	0.014925	0.00207	0.138716	1
GO:0003735	structural constituent of ribosome	MF	1	1	67	483	0.014925	0.00207	0.138716	1
GO:0045901	positive regulation of translational elongation	BP	1	1	67	483	0.014925	0.00207	0.138716	1
GO:0006509	membrane protein ectodomain proteolysis	BP	1	1	67	483	0.014925	0.00207	0.138716	1
GO:0002702	positive regulation of production of molecular mediator of immune	BP	1	1	67	483	0.014925	0.00207	0.138716	1
GO:0002208	somatic diversification of immunoglobulins involved in immune re	BP	1	1	67	483	0.014925	0.00207	0.138716	1
GO:2000379	positive regulation of reactive oxygen species metabolic process	BP	1	1	67	483	0.014925	0.00207	0.138716	1
GO:0042728	flavin-containing compound catabolic process	BP	1	1	67	483	0.014925	0.00207	0.138716	1
GO:1901618	organic hydroxy compound transmembrane transporter activity	MF	1	1	67	483	0.014925	0.00207	0.138716	1
GO:0033135	regulation of peptidyl-serine phosphorylation	BP	1	1	67	483	0.014925	0.00207	0.138716	1
GO:0015980	energy derivation by oxidation of organic compounds	BP	1	1	67	483	0.014925	0.00207	0.138716	1
GO:0001931	uropod	CC	1	1	67	483	0.014925	0.00207	0.138716	1
GO:0070646	protein modification by small protein removal	BP	1	1	67	483	0.014925	0.00207	0.138716	1
GO:1900182	positive regulation of protein localization to nucleus	BP	1	1	67	483	0.014925	0.00207	0.138716	1
GO:0031593	polyubiquitin modification-dependent protein binding	MF	1	1	67	483	0.014925	0.00207	0.138716	1
GO:0036344	platelet morphogenesis	BP	1	1	67	483	0.014925	0.00207	0.138716	1
GO:0051657	maintenance of organelle location	BP	1	1	67	483	0.014925	0.00207	0.138716	1
GO:0034605	cellular response to heat	BP	1	1	67	483	0.014925	0.00207	0.138716	1
GO:0045095	keratin filament	CC	1	1	67	483	0.014925	0.00207	0.138716	1
GO:1903923	positive regulation of protein processing in phagocytic vesicle	BP	1	1	67	483	0.014925	0.00207	0.138716	1
GO:0006119	oxidative phosphorylation	BP	1	1	67	483	0.014925	0.00207	0.138716	1
GO:0042594	response to starvation	BP	1	1	67	483	0.014925	0.00207	0.138716	1
GO:0015934	large ribosomal subunit	CC	1	1	67	483	0.014925	0.00207	0.138716	1
GO:0002889	regulation of immunoglobulin mediated immune response	BP	1	1	67	483	0.014925	0.00207	0.138716	1
GO:0034098	VCP-NPL4-UFD1 AAA ATPase complex	CC	1	1	67	483	0.014925	0.00207	0.138716	1
GO:0070229	negative regulation of lymphocyte apoptotic process	BP	1	1	67	483	0.014925	0.00207	0.138716	1
GO:1903921	regulation of protein processing in phagocytic vesicle	BP	1	1	67	483	0.014925	0.00207	0.138716	1
GO:0043270	positive regulation of ion transport	BP	1	1	67	483	0.014925	0.00207	0.138716	1
GO:0009266	response to temperature stimulus	BP	1	1	67	483	0.014925	0.00207	0.138716	1
GO:0072389	flavin adenine dinucleotide catabolic process	BP	1	1	67	483	0.014925	0.00207	0.138716	1

GO:0019674	NAD metabolic process	BP	1	1	67	483	0.014925	0.00207	0.138716	1
GO:0070849	response to epidermal growth factor	BP	1	1	67	483	0.014925	0.00207	0.138716	1
GO:2000107	negative regulation of leukocyte apoptotic process	BP	1	1	67	483	0.014925	0.00207	0.138716	1
GO:0043568	positive regulation of insulin-like growth factor receptor signaling	BP	1	1	67	483	0.014925	0.00207	0.138716	1
GO:0050864	regulation of B cell activation	BP	1	1	67	483	0.014925	0.00207	0.138716	1
GO:0044070	regulation of anion transport	BP	1	1	67	483	0.014925	0.00207	0.138716	1
GO:0006448	regulation of translational elongation	BP	1	1	67	483	0.014925	0.00207	0.138716	1
GO:0001846	opsonin binding	MF	1	1	67	483	0.014925	0.00207	0.138716	1
GO:1900756	protein processing in phagocytic vesicle	BP	1	1	67	483	0.014925	0.00207	0.138716	1
GO:0006301	postreplication repair	BP	1	1	67	483	0.014925	0.00207	0.138716	1
GO:0140253	cell-cell fusion	BP	1	1	67	483	0.014925	0.00207	0.138716	1
GO:0071826	ribonucleoprotein complex subunit organization	BP	1	1	67	483	0.014925	0.00207	0.138716	1
GO:0036513	Derlin-1 retrotranslocation complex	CC	1	1	67	483	0.014925	0.00207	0.138716	1
GO:0051881	regulation of mitochondrial membrane potential	BP	1	1	67	483	0.014925	0.00207	0.138716	1
GO:0019864	IgG binding	MF	1	1	67	483	0.014925	0.00207	0.138716	1
GO:0045780	positive regulation of bone resorption	BP	1	1	67	483	0.014925	0.00207	0.138716	1
GO:0009060	aerobic respiration	BP	1	1	67	483	0.014925	0.00207	0.138716	1
GO:0033014	tetrapyrrole biosynthetic process	BP	1	1	67	483	0.014925	0.00207	0.138716	1
GO:1903004	regulation of protein K63-linked deubiquitination	BP	1	1	67	483	0.014925	0.00207	0.138716	1
GO:0008373	sialyltransferase activity	MF	1	1	67	483	0.014925	0.00207	0.138716	1
GO:0046852	positive regulation of bone remodeling	BP	1	1	67	483	0.014925	0.00207	0.138716	1
GO:0070842	aggresome assembly	BP	1	1	67	483	0.014925	0.00207	0.138716	1
GO:0015665	alcohol transmembrane transporter activity	MF	1	1	67	483	0.014925	0.00207	0.138716	1
GO:0032153	cell division site	CC	1	1	67	483	0.014925	0.00207	0.138716	1
GO:0032155	cell division site part	CC	1	1	67	483	0.014925	0.00207	0.138716	1
GO:0001778	plasma membrane repair	BP	1	1	67	483	0.014925	0.00207	0.138716	1
GO:0090085	regulation of protein deubiquitination	BP	1	1	67	483	0.014925	0.00207	0.138716	1
GO:0042100	B cell proliferation	BP	1	1	67	483	0.014925	0.00207	0.138716	1
GO:0008430	selenium binding	MF	1	1	67	483	0.014925	0.00207	0.138716	1
GO:0030169	low-density lipoprotein particle binding	MF	1	1	67	483	0.014925	0.00207	0.138716	1
GO:0016447	somatic recombination of immunoglobulin gene segments	BP	1	1	67	483	0.014925	0.00207	0.138716	1
GO:0099535	synapse-associated extracellular matrix	CC	1	1	67	483	0.014925	0.00207	0.138716	1

GO:0032509	endosome transport via multivesicular body sorting pathway	BP	1	1	67	483	0.014925	0.00207	0.138716	1
GO:0005811	lipid droplet	CC	1	1	67	483	0.014925	0.00207	0.138716	1
GO:0071243	cellular response to arsenic-containing substance	BP	1	1	67	483	0.014925	0.00207	0.138716	1
GO:0009408	response to heat	BP	1	1	67	483	0.014925	0.00207	0.138716	1
GO:0044391	ribosomal subunit	CC	1	1	67	483	0.014925	0.00207	0.138716	1
GO:1900542	regulation of purine nucleotide metabolic process	BP	1	1	67	483	0.014925	0.00207	0.138716	1
GO:0097352	autophagosome maturation	BP	1	1	67	483	0.014925	0.00207	0.138716	1
GO:0000212	meiotic spindle organization	BP	1	1	67	483	0.014925	0.00207	0.138716	1
GO:2001138	regulation of phospholipid transport	BP	1	1	67	483	0.014925	0.00207	0.138716	1
GO:0043021	ribonucleoprotein complex binding	MF	1	1	67	483	0.014925	0.00207	0.138716	1
GO:0001768	establishment of T cell polarity	BP	1	1	67	483	0.014925	0.00207	0.138716	1
GO:0060070	canonical Wnt signaling pathway	BP	1	1	67	483	0.014925	0.00207	0.138716	1
GO:0039694	viral RNA genome replication	BP	1	1	67	483	0.014925	0.00207	0.138716	1
GO:0090322	regulation of superoxide metabolic process	BP	1	1	67	483	0.014925	0.00207	0.138716	1
GO:1903843	cellular response to arsenite ion	BP	1	1	67	483	0.014925	0.00207	0.138716	1
GO:0006778	porphyrin-containing compound metabolic process	BP	1	1	67	483	0.014925	0.00207	0.138716	1
GO:0045911	positive regulation of DNA recombination	BP	1	1	67	483	0.014925	0.00207	0.138716	1
GO:0070536	protein K63-linked deubiquitination	BP	1	1	67	483	0.014925	0.00207	0.138716	1
GO:0042440	pigment metabolic process	BP	1	1	67	483	0.014925	0.00207	0.138716	1
GO:0060056	mammary gland involution	BP	1	1	67	483	0.014925	0.00207	0.138716	1
GO:0042554	superoxide anion generation	BP	1	1	67	483	0.014925	0.00207	0.138716	1
GO:0071985	multivesicular body sorting pathway	BP	1	1	67	483	0.014925	0.00207	0.138716	1
GO:0006515	protein quality control for misfolded or incompletely synthesized p	BP	1	1	67	483	0.014925	0.00207	0.138716	1
GO:0030220	platelet formation	BP	1	1	67	483	0.014925	0.00207	0.138716	1
GO:1903729	regulation of plasma membrane organization	BP	1	1	67	483	0.014925	0.00207	0.138716	1
GO:0002708	positive regulation of lymphocyte mediated immunity	BP	1	1	67	483	0.014925	0.00207	0.138716	1
GO:0004655	porphobilinogen synthase activity	MF	1	1	67	483	0.014925	0.00207	0.138716	1
GO:0055037	recycling endosome	CC	1	1	67	483	0.014925	0.00207	0.138716	1
GO:0006040	amino sugar metabolic process	BP	1	1	67	483	0.014925	0.00207	0.138716	1
GO:0035967	cellular response to topologically incorrect protein	BP	1	1	67	483	0.014925	0.00207	0.138716	1
GO:0002204	somatic recombination of immunoglobulin genes involved in immu	BP	1	1	67	483	0.014925	0.00207	0.138716	1
GO:2000106	regulation of leukocyte apoptotic process	BP	1	1	67	483	0.014925	0.00207	0.138716	1

GO:0010324	membrane invagination	BP	1	1	67	483	0.014925	0.00207	0.138716	1
GO:0071218	cellular response to misfolded protein	BP	1	1	67	483	0.014925	0.00207	0.138716	1
GO:0006054	N-acetylneuraminate metabolic process	BP	1	1	67	483	0.014925	0.00207	0.138716	1
GO:0002200	somatic diversification of immune receptors	BP	1	1	67	483	0.014925	0.00207	0.138716	1
GO:0006452	translational frameshifting	BP	1	1	67	483	0.014925	0.00207	0.138716	1
GO:0032446	protein modification by small protein conjugation	BP	1	1	67	483	0.014925	0.00207	0.138716	1
GO:0051295	establishment of meiotic spindle localization	BP	1	1	67	483	0.014925	0.00207	0.138716	1
GO:0009267	cellular response to starvation	BP	1	1	67	483	0.014925	0.00207	0.138716	1
GO:0000018	regulation of DNA recombination	BP	1	1	67	483	0.014925	0.00207	0.138716	1
GO:0042623	ATPase activity, coupled	MF	1	1	67	483	0.014925	0.00207	0.138716	1
GO:1903322	positive regulation of protein modification by small protein conjug	BP	1	1	67	483	0.014925	0.00207	0.138716	1
GO:1990730	VCP-NSFL1C complex	CC	1	1	67	483	0.014925	0.00207	0.138716	1
GO:0099024	plasma membrane invagination	BP	1	1	67	483	0.014925	0.00207	0.138716	1
GO:0002381	immunoglobulin production involved in immunoglobulin mediated	BP	1	1	67	483	0.014925	0.00207	0.138716	1
GO:0004998	transferrin receptor activity	MF	1	1	67	483	0.014925	0.00207	0.138716	1
GO:0016579	protein deubiquitination	BP	1	1	67	483	0.014925	0.00207	0.138716	1
GO:0002703	regulation of leukocyte mediated immunity	BP	1	1	67	483	0.014925	0.00207	0.138716	1
GO:1900544	positive regulation of purine nucleotide metabolic process	BP	1	1	67	483	0.014925	0.00207	0.138716	1
GO:0033013	tetrapyrrole metabolic process	BP	1	1	67	483	0.014925	0.00207	0.138716	1
GO:0071364	cellular response to epidermal growth factor stimulus	BP	1	1	67	483	0.014925	0.00207	0.138716	1
GO:0016918	retinal binding	MF	1	1	67	483	0.014925	0.00207	0.138716	1
GO:0001767	establishment of lymphocyte polarity	BP	1	1	67	483	0.014925	0.00207	0.138716	1
GO:0032801	receptor catabolic process	BP	1	1	67	483	0.014925	0.00207	0.138716	1
GO:1905684	regulation of plasma membrane repair	BP	1	1	67	483	0.014925	0.00207	0.138716	1
GO:0000146	microfilament motor activity	MF	1	1	67	483	0.014925	0.00207	0.138716	1
GO:0018209	peptidyl-serine modification	BP	1	1	67	483	0.014925	0.00207	0.138716	1
GO:1901881	positive regulation of protein depolymerization	BP	1	1	67	483	0.014925	0.00207	0.138716	1
GO:1900022	regulation of D-erythro-sphingosine kinase activity	BP	1	1	67	483	0.014925	0.00207	0.138716	1
GO:0030224	monocyte differentiation	BP	1	1	67	483	0.014925	0.00207	0.138716	1
GO:0030010	establishment of cell polarity	BP	1	1	67	483	0.014925	0.00207	0.138716	1
GO:0061857	endoplasmic reticulum stress-induced pre-emptive quality control	BP	1	1	67	483	0.014925	0.00207	0.138716	1
GO:0070938	contractile ring	CC	1	1	67	483	0.014925	0.00207	0.138716	1

GO:1903862	positive regulation of oxidative phosphorylation	BP	1	1	67	483	0.014925	0.00207	0.138716	1
GO:0045333	cellular respiration	BP	1	1	67	483	0.014925	0.00207	0.138716	1
GO:0007520	myoblast fusion	BP	1	1	67	483	0.014925	0.00207	0.138716	1
GO:0030890	positive regulation of B cell proliferation	BP	1	1	67	483	0.014925	0.00207	0.138716	1
GO:2001169	regulation of ATP biosynthetic process	BP	1	1	67	483	0.014925	0.00207	0.138716	1
GO:1902995	positive regulation of phospholipid efflux	BP	1	1	67	483	0.014925	0.00207	0.138716	1
GO:0043457	regulation of cellular respiration	BP	1	1	67	483	0.014925	0.00207	0.138716	1
GO:0016836	hydro-lyase activity	MF	1	1	67	483	0.014925	0.00207	0.138716	1
GO:0030836	positive regulation of actin filament depolymerization	BP	1	1	67	483	0.014925	0.00207	0.138716	1
GO:1902994	regulation of phospholipid efflux	BP	1	1	67	483	0.014925	0.00207	0.138716	1
GO:0016567	protein ubiquitination	BP	1	1	67	483	0.014925	0.00207	0.138716	1
GO:1990712	HFE-transferrin receptor complex	CC	1	1	67	483	0.014925	0.00207	0.138716	1
GO:0002377	immunoglobulin production	BP	1	1	67	483	0.014925	0.00207	0.138716	1
GO:0005826	actomyosin contractile ring	CC	1	1	67	483	0.014925	0.00207	0.138716	1
GO:0015682	ferric iron transport	BP	1	1	67	483	0.014925	0.00207	0.138716	1
GO:0045981	positive regulation of nucleotide metabolic process	BP	1	1	67	483	0.014925	0.00207	0.138716	1
GO:0030810	positive regulation of nucleotide biosynthetic process	BP	1	1	67	483	0.014925	0.00207	0.138716	1
GO:0035861	site of double-strand break	CC	1	1	67	483	0.014925	0.00207	0.138716	1
GO:0060512	prostate gland morphogenesis	BP	1	1	67	483	0.014925	0.00207	0.138716	1
GO:0046148	pigment biosynthetic process	BP	1	1	67	483	0.014925	0.00207	0.138716	1
GO:1903320	regulation of protein modification by small protein conjugation or	BP	1	1	67	483	0.014925	0.00207	0.138716	1
GO:0043022	ribosome binding	MF	1	1	67	483	0.014925	0.00207	0.138716	1
GO:0000910	cytokinesis	BP	1	1	67	483	0.014925	0.00207	0.138716	1
GO:0042391	regulation of membrane potential	BP	1	1	67	483	0.014925	0.00207	0.138716	1
GO:0007528	neuromuscular junction development	BP	1	1	67	483	0.014925	0.00207	0.138716	1
GO:0032418	lysosome localization	BP	1	1	67	483	0.014925	0.00207	0.138716	1
GO:0060828	regulation of canonical Wnt signaling pathway	BP	1	1	67	483	0.014925	0.00207	0.138716	1
GO:0006734	NADH metabolic process	BP	1	1	67	483	0.014925	0.00207	0.138716	1
GO:0030808	regulation of nucleotide biosynthetic process	BP	1	1	67	483	0.014925	0.00207	0.138716	1
GO:0002312	B cell activation involved in immune response	BP	1	1	67	483	0.014925	0.00207	0.138716	1
GO:1903008	organelle disassembly	BP	1	1	67	483	0.014925	0.00207	0.138716	1
GO:2000377	regulation of reactive oxygen species metabolic process	BP	1	1	67	483	0.014925	0.00207	0.138716	1

GO:0005819	spindle	CC	1	1	67	483	0.014925	0.00207	0.138716	1
GO:0008333	endosome to lysosome transport	BP	1	1	67	483	0.014925	0.00207	0.138716	1
GO:0004930	G protein-coupled receptor activity	MF	1	1	67	483	0.014925	0.00207	0.138716	1
GO:0045830	positive regulation of isotype switching	BP	1	1	67	483	0.014925	0.00207	0.138716	1
GO:1903842	response to arsenite ion	BP	1	1	67	483	0.014925	0.00207	0.138716	1
GO:0000731	DNA synthesis involved in DNA repair	BP	1	1	67	483	0.014925	0.00207	0.138716	1
GO:0042113	B cell activation	BP	1	1	67	483	0.014925	0.00207	0.138716	1
GO:0002705	positive regulation of leukocyte mediated immunity	BP	1	1	67	483	0.014925	0.00207	0.138716	1
GO:0010494	cytoplasmic stress granule	CC	1	1	67	483	0.014925	0.00207	0.138716	1
GO:0002082	regulation of oxidative phosphorylation	BP	1	1	67	483	0.014925	0.00207	0.138716	1
GO:0051653	spindle localization	BP	1	1	67	483	0.014925	0.00207	0.138716	1
GO:0070231	T cell apoptotic process	BP	1	1	67	483	0.014925	0.00207	0.138716	1
GO:0033138	positive regulation of peptidyl-serine phosphorylation	BP	1	1	67	483	0.014925	0.00207	0.138716	1
GO:0003729	mRNA binding	MF	1	1	67	483	0.014925	0.00207	0.138716	1
GO:1904949	ATPase complex	CC	1	1	67	483	0.014925	0.00207	0.138716	1
GO:0070647	protein modification by small protein conjugation or removal	BP	1	1	67	483	0.014925	0.00207	0.138716	1
GO:1903578	regulation of ATP metabolic process	BP	1	1	67	483	0.014925	0.00207	0.138716	1
GO:0043413	macromolecule glycosylation	BP	1	1	67	483	0.014925	0.00207	0.138716	1
GO:0051301	cell division	BP	1	1	67	483	0.014925	0.00207	0.138716	1
GO:0002562	somatic diversification of immune receptors via germline recombir	BP	1	1	67	483	0.014925	0.00207	0.138716	1
GO:0072387	flavin adenine dinucleotide metabolic process	BP	1	1	67	483	0.014925	0.00207	0.138716	1
GO:1903003	positive regulation of protein deubiquitination	BP	1	1	67	483	0.014925	0.00207	0.138716	1
GO:0030898	actin-dependent ATPase activity	MF	1	1	67	483	0.014925	0.00207	0.138716	1
GO:0006140	regulation of nucleotide metabolic process	BP	1	1	67	483	0.014925	0.00207	0.138716	1
GO:0008180	COP9 signalosome	CC	1	1	67	483	0.014925	0.00207	0.138716	1
GO:0071712	ER-associated misfolded protein catabolic process	BP	1	1	67	483	0.014925	0.00207	0.138716	1
GO:0032988	ribonucleoprotein complex disassembly	BP	1	1	67	483	0.014925	0.00207	0.138716	1
GO:0000768	syncytium formation by plasma membrane fusion	BP	1	1	67	483	0.014925	0.00207	0.138716	1
GO:0070232	regulation of T cell apoptotic process	BP	1	1	67	483	0.014925	0.00207	0.138716	1
GO:1903715	regulation of aerobic respiration	BP	1	1	67	483	0.014925	0.00207	0.138716	1
GO:1903580	positive regulation of ATP metabolic process	BP	1	1	67	483	0.014925	0.00207	0.138716	1
GO:0005614	interstitial matrix	CC	1	1	67	483	0.014925	0.00207	0.138716	1

GO:0031623	receptor internalization	BP	1	1	67	483	0.014925	0.00207	0.138716	1
GO:0043495	protein membrane anchor	MF	1	1	67	483	0.014925	0.00207	0.138716	1
GO:0032510	endosome to lysosome transport via multivesicular body sorting pa	BP	1	1	67	483	0.014925	0.00207	0.138716	1
GO:0002637	regulation of immunoglobulin production	BP	1	1	67	483	0.014925	0.00207	0.138716	1
GO:0140110	transcription regulator activity	MF	1	1	67	483	0.014925	0.00207	0.138716	1
GO:0010918	positive regulation of mitochondrial membrane potential	BP	1	1	67	483	0.014925	0.00207	0.138716	1
GO:0015643	toxic substance binding	MF	1	1	67	483	0.014925	0.00207	0.138716	1
GO:0090263	positive regulation of canonical Wnt signaling pathway	BP	1	1	67	483	0.014925	0.00207	0.138716	1
GO:0009101	glycoprotein biosynthetic process	BP	1	1	67	483	0.014925	0.00207	0.138716	1
GO:0002706	regulation of lymphocyte mediated immunity	BP	1	1	67	483	0.014925	0.00207	0.138716	1
GO:0030177	positive regulation of Wnt signaling pathway	BP	1	1	67	483	0.014925	0.00207	0.138716	1
GO:0060443	mammary gland morphogenesis	BP	1	1	67	483	0.014925	0.00207	0.138716	1
GO:0032154	cleavage furrow	CC	1	1	67	483	0.014925	0.00207	0.138716	1
GO:0006783	heme biosynthetic process	BP	1	1	67	483	0.014925	0.00207	0.138716	1
GO:2001171	positive regulation of ATP biosynthetic process	BP	1	1	67	483	0.014925	0.00207	0.138716	1
GO:0044342	type B pancreatic cell proliferation	BP	1	1	67	483	0.014925	0.00207	0.138716	1
GO:1990381	ubiquitin-specific protease binding	MF	1	1	67	483	0.014925	0.00207	0.138716	1
GO:0032930	positive regulation of superoxide anion generation	BP	1	1	67	483	0.014925	0.00207	0.138716	1
GO:2001140	positive regulation of phospholipid transport	BP	1	1	67	483	0.014925	0.00207	0.138716	1
GO:0006486	protein glycosylation	BP	1	1	67	483	0.014925	0.00207	0.138716	1
GO:0045124	regulation of bone resorption	BP	1	1	67	483	0.014925	0.00207	0.138716	1
GO:0000981	DNA-binding transcription factor activity, RNA polymerase II-spe	MF	1	1	67	483	0.014925	0.00207	0.138716	1
GO:0003700	DNA-binding transcription factor activity	MF	1	1	67	483	0.014925	0.00207	0.138716	1
GO:0140030	modification-dependent protein binding	MF	1	1	67	483	0.014925	0.00207	0.138716	1
GO:0018105	peptidyl-serine phosphorylation	BP	1	1	67	483	0.014925	0.00207	0.138716	1
GO:0070233	negative regulation of T cell apoptotic process	BP	1	1	67	483	0.014925	0.00207	0.138716	1
GO:0071887	leukocyte apoptotic process	BP	1	1	67	483	0.014925	0.00207	0.138716	1
GO:0030316	osteoclast differentiation	BP	1	1	67	483	0.014925	0.00207	0.138716	1
GO:0001849	complement component C1q binding	MF	1	1	67	483	0.014925	0.00207	0.138716	1
GO:1900373	positive regulation of purine nucleotide biosynthetic process	BP	1	1	67	483	0.014925	0.00207	0.138716	1
GO:0099544	perisynaptic space	CC	1	1	67	483	0.014925	0.00207	0.138716	1
GO:1904288	BAT3 complex binding	MF	1	1	67	483	0.014925	0.00207	0.138716	1

GO:0045191	regulation of isotype switching	BP	1	1	67	483	0.014925	0.00207	0.138716	1
GO:0035617	stress granule disassembly	BP	1	1	67	483	0.014925	0.00207	0.138716	1
GO:0036435	K48-linked polyubiquitin modification-dependent protein binding	MF	1	1	67	483	0.014925	0.00207	0.138716	1
GO:0046685	response to arsenic-containing substance	BP	1	1	67	483	0.014925	0.00207	0.138716	1
GO:0022625	cytosolic large ribosomal subunit	CC	1	1	67	483	0.014925	0.00207	0.138716	1
GO:0032928	regulation of superoxide anion generation	BP	1	1	67	483	0.014925	0.00207	0.138716	1
GO:0033619	membrane protein proteolysis	BP	1	1	67	483	0.014925	0.00207	0.138716	1
GO:0045545	syndecan binding	MF	1	1	67	483	0.014925	0.00207	0.138716	1
GO:0002712	regulation of B cell mediated immunity	BP	1	1	67	483	0.014925	0.00207	0.138716	1
GO:1903131	mononuclear cell differentiation	BP	1	1	67	483	0.014925	0.00207	0.138716	1
GO:0002714	positive regulation of B cell mediated immunity	BP	1	1	67	483	0.014925	0.00207	0.138716	1
GO:0060739	mesenchymal-epithelial cell signaling involved in prostate gland de	BP	1	1	67	483	0.014925	0.00207	0.138716	1
GO:1903006	positive regulation of protein K63-linked deubiquitination	BP	1	1	67	483	0.014925	0.00207	0.138716	1
GO:0002639	positive regulation of immunoglobulin production	BP	1	1	67	483	0.014925	0.00207	0.138716	1
GO:0070085	glycosylation	BP	1	1	67	483	0.014925	0.00207	0.138716	1
GO:0051293	establishment of spindle localization	BP	1	1	67	483	0.014925	0.00207	0.138716	1
GO:1903829	positive regulation of cellular protein localization	BP	1	1	67	483	0.014925	0.00207	0.138716	1
GO:0004984	olfactory receptor activity	MF	1	1	67	483	0.014925	0.00207	0.138716	1
GO:0097264	self proteolysis	BP	1	1	67	483	0.014925	0.00207	0.138716	1
GO:0009109	coenzyme catabolic process	BP	1	1	67	483	0.014925	0.00207	0.138716	1
GO:0042168	heme metabolic process	BP	1	1	67	483	0.014925	0.00207	0.138716	1
GO:0051646	mitochondrion localization	BP	1	1	67	483	0.014925	0.00207	0.138716	1
GO:0032506	cytokinetic process	BP	1	1	67	483	0.014925	0.00207	0.138716	1
GO:0098966	perisynaptic extracellular matrix	CC	1	1	67	483	0.014925	0.00207	0.138716	1
GO:0045190	isotype switching	BP	1	1	67	483	0.014925	0.00207	0.138716	1
GO:0060638	mesenchymal-epithelial cell signaling	BP	1	1	67	483	0.014925	0.00207	0.138716	1
GO:0016445	somatic diversification of immunoglobulins	BP	1	1	67	483	0.014925	0.00207	0.138716	1
GO:0003835	beta-galactoside alpha-2,6-sialyltransferase activity	MF	1	1	67	483	0.014925	0.00207	0.138716	1
GO:0018196	peptidyl-asparagine modification	BP	1	1	67	483	0.014925	0.00207	0.138716	1
GO:0006911	phagocytosis, engulfment	BP	1	1	67	483	0.014925	0.00207	0.138716	1
GO:0006487	protein N-linked glycosylation	BP	1	1	67	483	0.014925	0.00207	0.138716	1
GO:0016444	somatic cell DNA recombination	BP	1	1	67	483	0.014925	0.00207	0.138716	1

GO:0030879	mammary gland development	BP	1	1	67	483	0.014925	0.00207	0.138716	1
GO:0098847	sequence-specific single stranded DNA binding	MF	1	1	67	483	0.014925	0.00207	0.138716	1
GO:0042726	flavin-containing compound metabolic process	BP	1	1	67	483	0.014925	0.00207	0.138716	1
GO:0035800	deubiquitinase activator activity	MF	1	1	67	483	0.014925	0.00207	0.138716	1
GO:0006949	syncytium formation	BP	1	1	67	483	0.014925	0.00207	0.138716	1
GO:0003779	actin binding	MF	4	15	67	483	0.059701	0.031056	0.140842	0.266667
GO:0098588	bounding membrane of organelle	CC	2	5	67	483	0.029851	0.010352	0.143561	0.4
GO:0005768	endosome	CC	2	5	67	483	0.029851	0.010352	0.143561	0.4
GO:1903362	regulation of cellular protein catabolic process	BP	2	5	67	483	0.029851	0.010352	0.143561	0.4
GO:0006898	receptor-mediated endocytosis	BP	2	5	67	483	0.029851	0.010352	0.143561	0.4
GO:0051186	cofactor metabolic process	BP	2	5	67	483	0.029851	0.010352	0.143561	0.4
GO:0035770	ribonucleoprotein granule	CC	2	5	67	483	0.029851	0.010352	0.143561	0.4
GO:0016032	viral process	BP	2	5	67	483	0.029851	0.010352	0.143561	0.4
GO:0048732	gland development	BP	2	5	67	483	0.029851	0.010352	0.143561	0.4
GO:0031984	organelle subcompartment	CC	2	5	67	483	0.029851	0.010352	0.143561	0.4
GO:0070663	regulation of leukocyte proliferation	BP	2	5	67	483	0.029851	0.010352	0.143561	0.4
GO:0036464	cytoplasmic ribonucleoprotein granule	CC	2	5	67	483	0.029851	0.010352	0.143561	0.4
GO:0031334	positive regulation of protein complex assembly	BP	2	5	67	483	0.029851	0.010352	0.143561	0.4
GO:0010035	response to inorganic substance	BP	2	5	67	483	0.029851	0.010352	0.143561	0.4
GO:0032944	regulation of mononuclear cell proliferation	BP	2	5	67	483	0.029851	0.010352	0.143561	0.4
GO:0043229	intracellular organelle	CC	19	109	67	483	0.283582	0.225673	0.143978	0.174312
GO:0045935	positive regulation of nucleobase-containing compound metabolic	BP	3	10	67	483	0.044776	0.020704	0.149462	0.3
GO:1990904	ribonucleoprotein complex	CC	3	10	67	483	0.044776	0.020704	0.149462	0.3
GO:0005622	intracellular	CC	21	123	67	483	0.313433	0.254658	0.149625	0.170732
GO:0044424	intracellular part	CC	21	123	67	483	0.313433	0.254658	0.149625	0.170732
GO:0032270	positive regulation of cellular protein metabolic process	BP	5	21	67	483	0.074627	0.043478	0.151818	0.238095
GO:0043226	organelle	CC	19	110	67	483	0.283582	0.227743	0.154473	0.172727
GO:0009058	biosynthetic process	BP	8	39	67	483	0.119403	0.080745	0.15563	0.205128
GO:0097159	organic cyclic compound binding	MF	11	59	67	483	0.164179	0.122153	0.174326	0.186441
GO:0018130	heterocycle biosynthetic process	BP	5	22	67	483	0.074627	0.045549	0.17613	0.227273
GO:0019438	aromatic compound biosynthetic process	BP	5	22	67	483	0.074627	0.045549	0.17613	0.227273
GO:0051128	regulation of cellular component organization	BP	7	34	67	483	0.104478	0.070393	0.176437	0.205882

GO:0051173	positive regulation of nitrogen compound metabolic process	BP	6	28	67	483	0.089552	0.057971	0.177762	0.214286
GO:0031326	regulation of cellular biosynthetic process	BP	6	28	67	483	0.089552	0.057971	0.177762	0.214286
GO:0005794	Golgi apparatus	CC	3	11	67	483	0.044776	0.022774	0.185894	0.272727
GO:0018193	peptidyl-amino acid modification	BP	3	11	67	483	0.044776	0.022774	0.185894	0.272727
GO:0005515	protein binding	MF	17	100	67	483	0.253731	0.207039	0.194796	0.17
GO:0030055	cell-substrate junction	CC	2	6	67	483	0.029851	0.012422	0.196593	0.333333
GO:0006417	regulation of translation	BP	2	6	67	483	0.029851	0.012422	0.196593	0.333333
GO:0070661	leukocyte proliferation	BP	2	6	67	483	0.029851	0.012422	0.196593	0.333333
GO:0019842	vitamin binding	MF	2	6	67	483	0.029851	0.012422	0.196593	0.333333
GO:0005925	focal adhesion	CC	2	6	67	483	0.029851	0.012422	0.196593	0.333333
GO:0042176	regulation of protein catabolic process	BP	2	6	67	483	0.029851	0.012422	0.196593	0.333333
GO:0019825	oxygen binding	MF	2	6	67	483	0.029851	0.012422	0.196593	0.333333
GO:0062012	regulation of small molecule metabolic process	BP	2	6	67	483	0.029851	0.012422	0.196593	0.333333
GO:0017144	drug metabolic process	BP	2	6	67	483	0.029851	0.012422	0.196593	0.333333
GO:0045862	positive regulation of proteolysis	BP	2	6	67	483	0.029851	0.012422	0.196593	0.333333
GO:0005924	cell-substrate adherens junction	CC	2	6	67	483	0.029851	0.012422	0.196593	0.333333
GO:0005520	insulin-like growth factor binding	MF	2	6	67	483	0.029851	0.012422	0.196593	0.333333
GO:0010608	posttranscriptional regulation of gene expression	BP	2	6	67	483	0.029851	0.012422	0.196593	0.333333
GO:0070161	anchoring junction	CC	2	6	67	483	0.029851	0.012422	0.196593	0.333333
GO:0032970	regulation of actin filament-based process	BP	2	6	67	483	0.029851	0.012422	0.196593	0.333333
GO:0032943	mononuclear cell proliferation	BP	2	6	67	483	0.029851	0.012422	0.196593	0.333333
GO:0005912	adherens junction	CC	2	6	67	483	0.029851	0.012422	0.196593	0.333333
GO:0044425	membrane part	CC	10	54	67	483	0.149254	0.111801	0.197172	0.185185
GO:0043067	regulation of programmed cell death	BP	4	17	67	483	0.059701	0.035197	0.198796	0.235294
GO:0051641	cellular localization	BP	4	17	67	483	0.059701	0.035197	0.198796	0.235294
GO:0042981	regulation of apoptotic process	BP	4	17	67	483	0.059701	0.035197	0.198796	0.235294
GO:0046983	protein dimerization activity	MF	4	17	67	483	0.059701	0.035197	0.198796	0.235294
GO:0008092	cytoskeletal protein binding	MF	4	17	67	483	0.059701	0.035197	0.198796	0.235294
GO:0009889	regulation of biosynthetic process	BP	6	29	67	483	0.089552	0.060041	0.200514	0.206897
GO:2000112	regulation of cellular macromolecule biosynthetic process	BP	5	23	67	483	0.074627	0.047619	0.201849	0.217391
GO:0045184	establishment of protein localization	BP	5	23	67	483	0.074627	0.047619	0.201849	0.217391
GO:0051247	positive regulation of protein metabolic process	BP	5	23	67	483	0.074627	0.047619	0.201849	0.217391

GO:0015031	protein transport	BP	5	23	67	483	0.074627	0.047619	0.201849	0.217391
GO:1901362	organic cyclic compound biosynthetic process	BP	5	23	67	483	0.074627	0.047619	0.201849	0.217391
GO:0048522	positive regulation of cellular process	BP	10	55	67	483	0.149254	0.113872	0.214474	0.181818
GO:0060548	negative regulation of cell death	BP	3	12	67	483	0.044776	0.024845	0.224384	0.25
GO:0043066	negative regulation of apoptotic process	BP	3	12	67	483	0.044776	0.024845	0.224384	0.25
GO:0033554	cellular response to stress	BP	3	12	67	483	0.044776	0.024845	0.224384	0.25
GO:0043069	negative regulation of programmed cell death	BP	3	12	67	483	0.044776	0.024845	0.224384	0.25
GO:0015833	peptide transport	BP	5	24	67	483	0.074627	0.049689	0.228786	0.208333
GO:0042886	amide transport	BP	5	24	67	483	0.074627	0.049689	0.228786	0.208333
GO:0044260	cellular macromolecule metabolic process	BP	13	76	67	483	0.19403	0.15735	0.234987	0.171053
GO:0016021	integral component of membrane	CC	7	37	67	483	0.104478	0.076605	0.240593	0.189189
GO:0050794	regulation of cellular process	BP	17	104	67	483	0.253731	0.215321	0.24949	0.163462
GO:0002521	leukocyte differentiation	BP	2	7	67	483	0.029851	0.014493	0.251559	0.285714
GO:0043254	regulation of protein complex assembly	BP	2	7	67	483	0.029851	0.014493	0.251559	0.285714
GO:0043604	amide biosynthetic process	BP	2	7	67	483	0.029851	0.014493	0.251559	0.285714
GO:0043043	peptide biosynthetic process	BP	2	7	67	483	0.029851	0.014493	0.251559	0.285714
GO:0030054	cell junction	CC	2	7	67	483	0.029851	0.014493	0.251559	0.285714
GO:0006412	translation	BP	2	7	67	483	0.029851	0.014493	0.251559	0.285714
GO:1905952	regulation of lipid localization	BP	2	7	67	483	0.029851	0.014493	0.251559	0.285714
GO:0034248	regulation of cellular amide metabolic process	BP	2	7	67	483	0.029851	0.014493	0.251559	0.285714
GO:0048771	tissue remodeling	BP	2	7	67	483	0.029851	0.014493	0.251559	0.285714
GO:0006909	phagocytosis	BP	2	7	67	483	0.029851	0.014493	0.251559	0.285714
GO:0044445	cytosolic part	CC	2	7	67	483	0.029851	0.014493	0.251559	0.285714
GO:0051235	maintenance of location	BP	2	7	67	483	0.029851	0.014493	0.251559	0.285714
GO:0003677	DNA binding	MF	2	7	67	483	0.029851	0.014493	0.251559	0.285714
GO:0048471	perinuclear region of cytoplasm	CC	2	7	67	483	0.029851	0.014493	0.251559	0.285714
GO:0071705	nitrogen compound transport	BP	5	25	67	483	0.074627	0.05176	0.256744	0.2
GO:0010556	regulation of macromolecule biosynthetic process	BP	5	25	67	483	0.074627	0.05176	0.256744	0.2
GO:0008104	protein localization	BP	5	25	67	483	0.074627	0.05176	0.256744	0.2
GO:0009991	response to extracellular stimulus	BP	1	2	67	483	0.014925	0.004141	0.258438	0.5
GO:0033218	amide binding	MF	1	2	67	483	0.014925	0.004141	0.258438	0.5
GO:0005501	retinoid binding	MF	1	2	67	483	0.014925	0.004141	0.258438	0.5

GO:0051054	positive regulation of DNA metabolic process	BP	1	2	67	483	0.014925	0.004141	0.258438	0.5
GO:0007163	establishment or maintenance of cell polarity	BP	1	2	67	483	0.014925	0.004141	0.258438	0.5
GO:0031103	axon regeneration	BP	1	2	67	483	0.014925	0.004141	0.258438	0.5
GO:0010008	endosome membrane	CC	1	2	67	483	0.014925	0.004141	0.258438	0.5
GO:2001056	positive regulation of cysteine-type endopeptidase activity	BP	1	2	67	483	0.014925	0.004141	0.258438	0.5
GO:0098827	endoplasmic reticulum subcompartment	CC	1	2	67	483	0.014925	0.004141	0.258438	0.5
GO:0045732	positive regulation of protein catabolic process	BP	1	2	67	483	0.014925	0.004141	0.258438	0.5
GO:0006801	superoxide metabolic process	BP	1	2	67	483	0.014925	0.004141	0.258438	0.5
GO:0005840	ribosome	CC	1	2	67	483	0.014925	0.004141	0.258438	0.5
GO:0070279	vitamin B6 binding	MF	1	2	67	483	0.014925	0.004141	0.258438	0.5
GO:1903052	positive regulation of proteolysis involved in cellular protein catab	BP	1	2	67	483	0.014925	0.004141	0.258438	0.5
GO:0098802	plasma membrane receptor complex	CC	1	2	67	483	0.014925	0.004141	0.258438	0.5
GO:0003774	motor activity	MF	1	2	67	483	0.014925	0.004141	0.258438	0.5
GO:0004197	cysteine-type endopeptidase activity	MF	1	2	67	483	0.014925	0.004141	0.258438	0.5
GO:0010883	regulation of lipid storage	BP	1	2	67	483	0.014925	0.004141	0.258438	0.5
GO:2000060	positive regulation of ubiquitin-dependent protein catabolic proces	BP	1	2	67	483	0.014925	0.004141	0.258438	0.5
GO:0032436	positive regulation of proteasomal ubiquitin-dependent protein cata	BP	1	2	67	483	0.014925	0.004141	0.258438	0.5
GO:0002822	regulation of adaptive immune response based on somatic recombi	BP	1	2	67	483	0.014925	0.004141	0.258438	0.5
GO:0019902	phosphatase binding	MF	1	2	67	483	0.014925	0.004141	0.258438	0.5
GO:0022626	cytosolic ribosome	CC	1	2	67	483	0.014925	0.004141	0.258438	0.5
GO:0010874	regulation of cholesterol efflux	BP	1	2	67	483	0.014925	0.004141	0.258438	0.5
GO:0002700	regulation of production of molecular mediator of immune respons	BP	1	2	67	483	0.014925	0.004141	0.258438	0.5
GO:0002824	positive regulation of adaptive immune response based on somatic	BP	1	2	67	483	0.014925	0.004141	0.258438	0.5
GO:0044440	endosomal part	CC	1	2	67	483	0.014925	0.004141	0.258438	0.5
GO:0043281	regulation of cysteine-type endopeptidase activity involved in apop	BP	1	2	67	483	0.014925	0.004141	0.258438	0.5
GO:0034105	positive regulation of tissue remodeling	BP	1	2	67	483	0.014925	0.004141	0.258438	0.5
GO:0010745	negative regulation of macrophage derived foam cell differentiatio	BP	1	2	67	483	0.014925	0.004141	0.258438	0.5
GO:0043567	regulation of insulin-like growth factor receptor signaling pathway	BP	1	2	67	483	0.014925	0.004141	0.258438	0.5
GO:0016236	macroautophagy	BP	1	2	67	483	0.014925	0.004141	0.258438	0.5
GO:0030048	actin filament-based movement	BP	1	2	67	483	0.014925	0.004141	0.258438	0.5
GO:0051187	cofactor catabolic process	BP	1	2	67	483	0.014925	0.004141	0.258438	0.5
GO:0032796	uropod organization	BP	1	2	67	483	0.014925	0.004141	0.258438	0.5

GO:0090482	vitamin transmembrane transporter activity	MF	1	2	67	483	0.014925	0.004141	0.258438	0.5
GO:0034250	positive regulation of cellular amide metabolic process	BP	1	2	67	483	0.014925	0.004141	0.258438	0.5
GO:0051261	protein depolymerization	BP	1	2	67	483	0.014925	0.004141	0.258438	0.5
GO:0006310	DNA recombination	BP	1	2	67	483	0.014925	0.004141	0.258438	0.5
GO:0030834	regulation of actin filament depolymerization	BP	1	2	67	483	0.014925	0.004141	0.258438	0.5
GO:2000482	regulation of interleukin-8 secretion	BP	1	2	67	483	0.014925	0.004141	0.258438	0.5
GO:0045453	bone resorption	BP	1	2	67	483	0.014925	0.004141	0.258438	0.5
GO:0043565	sequence-specific DNA binding	MF	1	2	67	483	0.014925	0.004141	0.258438	0.5
GO:0005882	intermediate filament	CC	1	2	67	483	0.014925	0.004141	0.258438	0.5
GO:0006919	activation of cysteine-type endopeptidase activity involved in apop	BP	1	2	67	483	0.014925	0.004141	0.258438	0.5
GO:0006302	double-strand break repair	BP	1	2	67	483	0.014925	0.004141	0.258438	0.5
GO:0090734	site of DNA damage	CC	1	2	67	483	0.014925	0.004141	0.258438	0.5
GO:0045727	positive regulation of translation	BP	1	2	67	483	0.014925	0.004141	0.258438	0.5
GO:0031669	cellular response to nutrient levels	BP	1	2	67	483	0.014925	0.004141	0.258438	0.5
GO:1903513	endoplasmic reticulum to cytosol transport	BP	1	2	67	483	0.014925	0.004141	0.258438	0.5
GO:0070670	response to interleukin-4	BP	1	2	67	483	0.014925	0.004141	0.258438	0.5
GO:0031667	response to nutrient levels	BP	1	2	67	483	0.014925	0.004141	0.258438	0.5
GO:0071496	cellular response to external stimulus	BP	1	2	67	483	0.014925	0.004141	0.258438	0.5
GO:1901800	positive regulation of proteasomal protein catabolic process	BP	1	2	67	483	0.014925	0.004141	0.258438	0.5
GO:0070841	inclusion body assembly	BP	1	2	67	483	0.014925	0.004141	0.258438	0.5
GO:0006109	regulation of carbohydrate metabolic process	BP	1	2	67	483	0.014925	0.004141	0.258438	0.5
GO:0030170	pyridoxal phosphate binding	MF	1	2	67	483	0.014925	0.004141	0.258438	0.5
GO:0043534	blood vessel endothelial cell migration	BP	1	2	67	483	0.014925	0.004141	0.258438	0.5
GO:0014012	peripheral nervous system axon regeneration	BP	1	2	67	483	0.014925	0.004141	0.258438	0.5
GO:0051651	maintenance of location in cell	BP	1	2	67	483	0.014925	0.004141	0.258438	0.5
GO:0042288	MHC class I protein binding	MF	1	2	67	483	0.014925	0.004141	0.258438	0.5
GO:0070001	aspartic-type peptidase activity	MF	1	2	67	483	0.014925	0.004141	0.258438	0.5
GO:1901799	negative regulation of proteasomal protein catabolic process	BP	1	2	67	483	0.014925	0.004141	0.258438	0.5
GO:0002440	production of molecular mediator of immune response	BP	1	2	67	483	0.014925	0.004141	0.258438	0.5
GO:0009100	glycoprotein metabolic process	BP	1	2	67	483	0.014925	0.004141	0.258438	0.5
GO:0051321	meiotic cell cycle	BP	1	2	67	483	0.014925	0.004141	0.258438	0.5
GO:0019865	immunoglobulin binding	MF	1	2	67	483	0.014925	0.004141	0.258438	0.5

GO:0032370	positive regulation of lipid transport	BP	1	2	67	483	0.014925	0.004141	0.258438	0.5
GO:0042175	nuclear outer membrane-endoplasmic reticulum membrane networl	CC	1	2	67	483	0.014925	0.004141	0.258438	0.5
GO:0032373	positive regulation of sterol transport	BP	1	2	67	483	0.014925	0.004141	0.258438	0.5
GO:0016460	myosin II complex	CC	1	2	67	483	0.014925	0.004141	0.258438	0.5
GO:1903981	enterobactin binding	MF	1	2	67	483	0.014925	0.004141	0.258438	0.5
GO:0005764	lysosome	CC	1	2	67	483	0.014925	0.004141	0.258438	0.5
GO:0032376	positive regulation of cholesterol transport	BP	1	2	67	483	0.014925	0.004141	0.258438	0.5
GO:0030970	retrograde protein transport, ER to cytosol	BP	1	2	67	483	0.014925	0.004141	0.258438	0.5
GO:1903051	negative regulation of proteolysis involved in cellular protein catab	BP	1	2	67	483	0.014925	0.004141	0.258438	0.5
GO:0000139	Golgi membrane	CC	1	2	67	483	0.014925	0.004141	0.258438	0.5
GO:0000323	lytic vacuole	CC	1	2	67	483	0.014925	0.004141	0.258438	0.5
GO:0016607	nuclear speck	CC	1	2	67	483	0.014925	0.004141	0.258438	0.5
GO:0007041	lysosomal transport	BP	1	2	67	483	0.014925	0.004141	0.258438	0.5
GO:0031102	neuron projection regeneration	BP	1	2	67	483	0.014925	0.004141	0.258438	0.5
GO:0043280	positive regulation of cysteine-type endopeptidase activity involved	BP	1	2	67	483	0.014925	0.004141	0.258438	0.5
GO:0071813	lipoprotein particle binding	MF	1	2	67	483	0.014925	0.004141	0.258438	0.5
GO:0002699	positive regulation of immune effector process	BP	1	2	67	483	0.014925	0.004141	0.258438	0.5
GO:0034504	protein localization to nucleus	BP	1	2	67	483	0.014925	0.004141	0.258438	0.5
GO:0008234	cysteine-type peptidase activity	MF	1	2	67	483	0.014925	0.004141	0.258438	0.5
GO:0010952	positive regulation of peptidase activity	BP	1	2	67	483	0.014925	0.004141	0.258438	0.5
GO:0097433	dense body	CC	1	2	67	483	0.014925	0.004141	0.258438	0.5
GO:0036503	ERAD pathway	BP	1	2	67	483	0.014925	0.004141	0.258438	0.5
GO:0016323	basolateral plasma membrane	CC	1	2	67	483	0.014925	0.004141	0.258438	0.5
GO:0032945	negative regulation of mononuclear cell proliferation	BP	1	2	67	483	0.014925	0.004141	0.258438	0.5
GO:1901879	regulation of protein depolymerization	BP	1	2	67	483	0.014925	0.004141	0.258438	0.5
GO:0007009	plasma membrane organization	BP	1	2	67	483	0.014925	0.004141	0.258438	0.5
GO:0030850	prostate gland development	BP	1	2	67	483	0.014925	0.004141	0.258438	0.5
GO:0045879	negative regulation of smoothened signaling pathway	BP	1	2	67	483	0.014925	0.004141	0.258438	0.5
GO:0042287	MHC protein binding	MF	1	2	67	483	0.014925	0.004141	0.258438	0.5
GO:0007034	vacuolar transport	BP	1	2	67	483	0.014925	0.004141	0.258438	0.5
GO:0030042	actin filament depolymerization	BP	1	2	67	483	0.014925	0.004141	0.258438	0.5
GO:0051656	establishment of organelle localization	BP	1	2	67	483	0.014925	0.004141	0.258438	0.5

GO:0005773	vacuole	CC	1	2	67	483	0.014925	0.004141	0.258438	0.5
GO:0042277	peptide binding	MF	1	2	67	483	0.014925	0.004141	0.258438	0.5
GO:0019841	retinol binding	MF	1	2	67	483	0.014925	0.004141	0.258438	0.5
GO:1903046	meiotic cell cycle process	BP	1	2	67	483	0.014925	0.004141	0.258438	0.5
GO:0004190	aspartic-type endopeptidase activity	MF	1	2	67	483	0.014925	0.004141	0.258438	0.5
GO:0010950	positive regulation of endopeptidase activity	BP	1	2	67	483	0.014925	0.004141	0.258438	0.5
GO:0005789	endoplasmic reticulum membrane	CC	1	2	67	483	0.014925	0.004141	0.258438	0.5
GO:0001540	amyloid-beta binding	MF	1	2	67	483	0.014925	0.004141	0.258438	0.5
GO:0010675	regulation of cellular carbohydrate metabolic process	BP	1	2	67	483	0.014925	0.004141	0.258438	0.5
GO:0004519	endonuclease activity	MF	1	2	67	483	0.014925	0.004141	0.258438	0.5
GO:0071353	cellular response to interleukin-4	BP	1	2	67	483	0.014925	0.004141	0.258438	0.5
GO:0042953	lipoprotein transport	BP	1	2	67	483	0.014925	0.004141	0.258438	0.5
GO:0016604	nuclear body	CC	1	2	67	483	0.014925	0.004141	0.258438	0.5
GO:0070664	negative regulation of leukocyte proliferation	BP	1	2	67	483	0.014925	0.004141	0.258438	0.5
GO:0018206	peptidyl-methionine modification	BP	1	2	67	483	0.014925	0.004141	0.258438	0.5
GO:0071814	protein-lipid complex binding	MF	1	2	67	483	0.014925	0.004141	0.258438	0.5
GO:0019903	protein phosphatase binding	MF	1	2	67	483	0.014925	0.004141	0.258438	0.5
GO:0002821	positive regulation of adaptive immune response	BP	1	2	67	483	0.014925	0.004141	0.258438	0.5
GO:0016835	carbon-oxygen lyase activity	MF	1	2	67	483	0.014925	0.004141	0.258438	0.5
GO:0019840	isoprenoid binding	MF	1	2	67	483	0.014925	0.004141	0.258438	0.5
GO:0007051	spindle organization	BP	1	2	67	483	0.014925	0.004141	0.258438	0.5
GO:0010875	positive regulation of cholesterol efflux	BP	1	2	67	483	0.014925	0.004141	0.258438	0.5
GO:0047485	protein N-terminus binding	MF	1	2	67	483	0.014925	0.004141	0.258438	0.5
GO:0030433	ubiquitin-dependent ERAD pathway	BP	1	2	67	483	0.014925	0.004141	0.258438	0.5
GO:1903364	positive regulation of cellular protein catabolic process	BP	1	2	67	483	0.014925	0.004141	0.258438	0.5
GO:1900180	regulation of protein localization to nucleus	BP	1	2	67	483	0.014925	0.004141	0.258438	0.5
GO:0018158	protein oxidation	BP	1	2	67	483	0.014925	0.004141	0.258438	0.5
GO:0048678	response to axon injury	BP	1	2	67	483	0.014925	0.004141	0.258438	0.5
GO:0006281	DNA repair	BP	1	2	67	483	0.014925	0.004141	0.258438	0.5
GO:0010906	regulation of glucose metabolic process	BP	1	2	67	483	0.014925	0.004141	0.258438	0.5
GO:0002819	regulation of adaptive immune response	BP	1	2	67	483	0.014925	0.004141	0.258438	0.5
GO:0031594	neuromuscular junction	CC	1	2	67	483	0.014925	0.004141	0.258438	0.5

GO:0004198	calcium-dependent cysteine-type endopeptidase activity	MF	1	2	67	483	0.014925	0.004141	0.258438	0.5
GO:0016999	antibiotic metabolic process	BP	1	2	67	483	0.014925	0.004141	0.258438	0.5
GO:0048009	insulin-like growth factor receptor signaling pathway	BP	1	2	67	483	0.014925	0.004141	0.258438	0.5
GO:0044872	lipoprotein localization	BP	1	2	67	483	0.014925	0.004141	0.258438	0.5
GO:0045055	regulated exocytosis	BP	1	2	67	483	0.014925	0.004141	0.258438	0.5
GO:0031668	cellular response to extracellular stimulus	BP	1	2	67	483	0.014925	0.004141	0.258438	0.5
GO:0044446	intracellular organelle part	CC	9	51	67	483	0.134328	0.10559	0.262415	0.176471
GO:0006915	apoptotic process	BP	4	19	67	483	0.059701	0.039337	0.262736	0.210526
GO:0019219	regulation of nucleobase-containing compound metabolic process	BP	4	19	67	483	0.059701	0.039337	0.262736	0.210526
GO:0012501	programmed cell death	BP	4	19	67	483	0.059701	0.039337	0.262736	0.210526
GO:0042325	regulation of phosphorylation	BP	4	19	67	483	0.059701	0.039337	0.262736	0.210526
GO:0010941	regulation of cell death	BP	4	19	67	483	0.059701	0.039337	0.262736	0.210526
GO:0032268	regulation of cellular protein metabolic process	BP	7	38	67	483	0.104478	0.078675	0.263577	0.184211
GO:0031224	intrinsic component of membrane	CC	7	38	67	483	0.104478	0.078675	0.263577	0.184211
GO:0044422	organelle part	CC	9	52	67	483	0.134328	0.10766	0.282881	0.173077
GO:0044464	cell part	CC	23	148	67	483	0.343284	0.306418	0.283895	0.155405
GO:0048518	positive regulation of biological process	BP	13	79	67	483	0.19403	0.163561	0.284878	0.164557
GO:0046483	heterocycle metabolic process	BP	5	26	67	483	0.074627	0.05383	0.285522	0.192308
GO:0006725	cellular aromatic compound metabolic process	BP	5	26	67	483	0.074627	0.05383	0.285522	0.192308
GO:0009891	positive regulation of biosynthetic process	BP	3	14	67	483	0.044776	0.028986	0.305041	0.214286
GO:0031401	positive regulation of protein modification process	BP	3	14	67	483	0.044776	0.028986	0.305041	0.214286
GO:0045937	positive regulation of phosphate metabolic process	BP	3	14	67	483	0.044776	0.028986	0.305041	0.214286
GO:0010562	positive regulation of phosphorus metabolic process	BP	3	14	67	483	0.044776	0.028986	0.305041	0.214286
GO:0031328	positive regulation of cellular biosynthetic process	BP	3	14	67	483	0.044776	0.028986	0.305041	0.214286
GO:0042327	positive regulation of phosphorylation	BP	3	14	67	483	0.044776	0.028986	0.305041	0.214286
GO:0019838	growth factor binding	MF	2	8	67	483	0.029851	0.016563	0.30693	0.25
GO:0042110	T cell activation	BP	2	8	67	483	0.029851	0.016563	0.30693	0.25
GO:0044089	positive regulation of cellular component biogenesis	BP	2	8	67	483	0.029851	0.016563	0.30693	0.25
GO:0090066	regulation of anatomical structure size	BP	2	8	67	483	0.029851	0.016563	0.30693	0.25
GO:0055114	oxidation-reduction process	BP	2	8	67	483	0.029851	0.016563	0.30693	0.25
GO:0002520	immune system development	BP	2	8	67	483	0.029851	0.016563	0.30693	0.25
GO:0010498	proteasomal protein catabolic process	BP	2	8	67	483	0.029851	0.016563	0.30693	0.25

GO:0030097	hemopoiesis	BP	2	8	67	483	0.029851	0.016563	0.30693	0.25
GO:0031090	organelle membrane	CC	2	8	67	483	0.029851	0.016563	0.30693	0.25
GO:0048534	hematopoietic or lymphoid organ development	BP	2	8	67	483	0.029851	0.016563	0.30693	0.25
GO:0008144	drug binding	MF	5	27	67	483	0.074627	0.055901	0.314919	0.185185
GO:0005886	plasma membrane	CC	8	47	67	483	0.119403	0.097308	0.319196	0.170213
GO:0051171	regulation of nitrogen compound metabolic process	BP	9	54	67	483	0.134328	0.111801	0.325141	0.166667
GO:1901360	organic cyclic compound metabolic process	BP	6	34	67	483	0.089552	0.070393	0.327251	0.176471
GO:0051174	regulation of phosphorus metabolic process	BP	4	21	67	483	0.059701	0.043478	0.330109	0.190476
GO:0019220	regulation of phosphate metabolic process	BP	4	21	67	483	0.059701	0.043478	0.330109	0.190476
GO:0008219	cell death	BP	4	21	67	483	0.059701	0.043478	0.330109	0.190476
GO:0034654	nucleobase-containing compound biosynthetic process	BP	4	21	67	483	0.059701	0.043478	0.330109	0.190476
GO:0071944	cell periphery	CC	8	48	67	483	0.119403	0.099379	0.342139	0.166667
GO:0043412	macromolecule modification	BP	5	28	67	483	0.074627	0.057971	0.344735	0.178571
GO:0006464	cellular protein modification process	BP	5	28	67	483	0.074627	0.057971	0.344735	0.178571
GO:0036211	protein modification process	BP	5	28	67	483	0.074627	0.057971	0.344735	0.178571
GO:0009967	positive regulation of signal transduction	BP	3	15	67	483	0.044776	0.031056	0.346091	0.2
GO:0005623	cell	CC	23	153	67	483	0.343284	0.31677	0.355031	0.150327
GO:0043170	macromolecule metabolic process	BP	18	118	67	483	0.268657	0.244306	0.358307	0.152542
GO:0006518	peptide metabolic process	BP	2	9	67	483	0.029851	0.018634	0.361551	0.222222
GO:0003924	GTPase activity	MF	2	9	67	483	0.029851	0.018634	0.361551	0.222222
GO:0046649	lymphocyte activation	BP	2	9	67	483	0.029851	0.018634	0.361551	0.222222
GO:0031329	regulation of cellular catabolic process	BP	2	9	67	483	0.029851	0.018634	0.361551	0.222222
GO:0043603	cellular amide metabolic process	BP	2	9	67	483	0.029851	0.018634	0.361551	0.222222
GO:0072593	reactive oxygen species metabolic process	BP	1	3	67	483	0.014925	0.006211	0.361733	0.333333
GO:0006733	oxidoreduction coenzyme metabolic process	BP	1	3	67	483	0.014925	0.006211	0.361733	0.333333
GO:0009161	ribonucleoside monophosphate metabolic process	BP	1	3	67	483	0.014925	0.006211	0.361733	0.333333
GO:0034103	regulation of tissue remodeling	BP	1	3	67	483	0.014925	0.006211	0.361733	0.333333
GO:0043123	positive regulation of I-kappaB kinase/NF-kappaB signaling	BP	1	3	67	483	0.014925	0.006211	0.361733	0.333333
GO:0009141	nucleoside triphosphate metabolic process	BP	1	3	67	483	0.014925	0.006211	0.361733	0.333333
GO:0072522	purine-containing compound biosynthetic process	BP	1	3	67	483	0.014925	0.006211	0.361733	0.333333
GO:0031331	positive regulation of cellular catabolic process	BP	1	3	67	483	0.014925	0.006211	0.361733	0.333333
GO:0008589	regulation of smoothened signaling pathway	BP	1	3	67	483	0.014925	0.006211	0.361733	0.333333

GO:0005739	mitochondrion	CC	1	3	67	483	0.014925	0.006211	0.361733	0.333333
GO:1905114	cell surface receptor signaling pathway involved in cell-cell signali	BP	1	3	67	483	0.014925	0.006211	0.361733	0.333333
GO:0009144	purine nucleoside triphosphate metabolic process	BP	1	3	67	483	0.014925	0.006211	0.361733	0.333333
GO:0033365	protein localization to organelle	BP	1	3	67	483	0.014925	0.006211	0.361733	0.333333
GO:0097756	negative regulation of blood vessel diameter	BP	1	3	67	483	0.014925	0.006211	0.361733	0.333333
GO:0051092	positive regulation of NF-kappaB transcription factor activity	BP	1	3	67	483	0.014925	0.006211	0.361733	0.333333
GO:0032527	protein exit from endoplasmic reticulum	BP	1	3	67	483	0.014925	0.006211	0.361733	0.333333
GO:0009156	ribonucleoside monophosphate biosynthetic process	BP	1	3	67	483	0.014925	0.006211	0.361733	0.333333
GO:0046390	ribose phosphate biosynthetic process	BP	1	3	67	483	0.014925	0.006211	0.361733	0.333333
GO:1990823	response to leukemia inhibitory factor	BP	1	3	67	483	0.014925	0.006211	0.361733	0.333333
GO:0062013	positive regulation of small molecule metabolic process	BP	1	3	67	483	0.014925	0.006211	0.361733	0.333333
GO:0010743	regulation of macrophage derived foam cell differentiation	BP	1	3	67	483	0.014925	0.006211	0.361733	0.333333
GO:0009127	purine nucleoside monophosphate biosynthetic process	BP	1	3	67	483	0.014925	0.006211	0.361733	0.333333
GO:0051188	cofactor biosynthetic process	BP	1	3	67	483	0.014925	0.006211	0.361733	0.333333
GO:0003725	double-stranded RNA binding	MF	1	3	67	483	0.014925	0.006211	0.361733	0.333333
GO:0001726	ruffle	CC	1	3	67	483	0.014925	0.006211	0.361733	0.333333
GO:0050907	detection of chemical stimulus involved in sensory perception	BP	1	3	67	483	0.014925	0.006211	0.361733	0.333333
GO:0044448	cell cortex part	CC	1	3	67	483	0.014925	0.006211	0.361733	0.333333
GO:0009145	purine nucleoside triphosphate biosynthetic process	BP	1	3	67	483	0.014925	0.006211	0.361733	0.333333
GO:0016459	myosin complex	CC	1	3	67	483	0.014925	0.006211	0.361733	0.333333
GO:0006753	nucleoside phosphate metabolic process	BP	1	3	67	483	0.014925	0.006211	0.361733	0.333333
GO:0009124	nucleoside monophosphate biosynthetic process	BP	1	3	67	483	0.014925	0.006211	0.361733	0.333333
GO:0032946	positive regulation of mononuclear cell proliferation	BP	1	3	67	483	0.014925	0.006211	0.361733	0.333333
GO:0009201	ribonucleoside triphosphate biosynthetic process	BP	1	3	67	483	0.014925	0.006211	0.361733	0.333333
GO:0050671	positive regulation of lymphocyte proliferation	BP	1	3	67	483	0.014925	0.006211	0.361733	0.333333
GO:0008083	growth factor activity	MF	1	3	67	483	0.014925	0.006211	0.361733	0.333333
GO:0070665	positive regulation of leukocyte proliferation	BP	1	3	67	483	0.014925	0.006211	0.361733	0.333333
GO:0016757	transferase activity, transferring glycosyl groups	MF	1	3	67	483	0.014925	0.006211	0.361733	0.333333
GO:1990830	cellular response to leukemia inhibitory factor	BP	1	3	67	483	0.014925	0.006211	0.361733	0.333333
GO:1901292	nucleoside phosphate catabolic process	BP	1	3	67	483	0.014925	0.006211	0.361733	0.333333
GO:0198738	cell-cell signaling by wnt	BP	1	3	67	483	0.014925	0.006211	0.361733	0.333333
GO:0030111	regulation of Wnt signaling pathway	BP	1	3	67	483	0.014925	0.006211	0.361733	0.333333

GO:0004518	nuclease activity	MF	1	3	67	483	0.014925	0.006211	0.361733	0.333333
GO:0010038	response to metal ion	BP	1	3	67	483	0.014925	0.006211	0.361733	0.333333
GO:0046496	nicotinamide nucleotide metabolic process	BP	1	3	67	483	0.014925	0.006211	0.361733	0.333333
GO:0072606	interleukin-8 secretion	BP	1	3	67	483	0.014925	0.006211	0.361733	0.333333
GO:2000058	regulation of ubiquitin-dependent protein catabolic process	BP	1	3	67	483	0.014925	0.006211	0.361733	0.333333
GO:0016791	phosphatase activity	MF	1	3	67	483	0.014925	0.006211	0.361733	0.333333
GO:0016197	endosomal transport	BP	1	3	67	483	0.014925	0.006211	0.361733	0.333333
GO:0050913	sensory perception of bitter taste	BP	1	3	67	483	0.014925	0.006211	0.361733	0.333333
GO:0016504	peptidase activator activity	MF	1	3	67	483	0.014925	0.006211	0.361733	0.333333
GO:0009165	nucleotide biosynthetic process	BP	1	3	67	483	0.014925	0.006211	0.361733	0.333333
GO:1905954	positive regulation of lipid localization	BP	1	3	67	483	0.014925	0.006211	0.361733	0.333333
GO:0045177	apical part of cell	CC	1	3	67	483	0.014925	0.006211	0.361733	0.333333
GO:0010954	positive regulation of protein processing	BP	1	3	67	483	0.014925	0.006211	0.361733	0.333333
GO:0006732	coenzyme metabolic process	BP	1	3	67	483	0.014925	0.006211	0.361733	0.333333
GO:0016829	lyase activity	MF	1	3	67	483	0.014925	0.006211	0.361733	0.333333
GO:0045111	intermediate filament cytoskeleton	CC	1	3	67	483	0.014925	0.006211	0.361733	0.333333
GO:0014902	myotube differentiation	BP	1	3	67	483	0.014925	0.006211	0.361733	0.333333
GO:0051146	striated muscle cell differentiation	BP	1	3	67	483	0.014925	0.006211	0.361733	0.333333
GO:0030864	cortical actin cytoskeleton	CC	1	3	67	483	0.014925	0.006211	0.361733	0.333333
GO:0046849	bone remodeling	BP	1	3	67	483	0.014925	0.006211	0.361733	0.333333
GO:0007606	sensory perception of chemical stimulus	BP	1	3	67	483	0.014925	0.006211	0.361733	0.333333
GO:0061024	membrane organization	BP	1	3	67	483	0.014925	0.006211	0.361733	0.333333
GO:0034404	nucleobase-containing small molecule biosynthetic process	BP	1	3	67	483	0.014925	0.006211	0.361733	0.333333
GO:0006914	autophagy	BP	1	3	67	483	0.014925	0.006211	0.361733	0.333333
GO:0071897	DNA biosynthetic process	BP	1	3	67	483	0.014925	0.006211	0.361733	0.333333
GO:0000226	microtubule cytoskeleton organization	BP	1	3	67	483	0.014925	0.006211	0.361733	0.333333
GO:0051253	negative regulation of RNA metabolic process	BP	1	3	67	483	0.014925	0.006211	0.361733	0.333333
GO:0006164	purine nucleotide biosynthetic process	BP	1	3	67	483	0.014925	0.006211	0.361733	0.333333
GO:0033293	monocarboxylic acid binding	MF	1	3	67	483	0.014925	0.006211	0.361733	0.333333
GO:0051091	positive regulation of DNA-binding transcription factor activity	BP	1	3	67	483	0.014925	0.006211	0.361733	0.333333
GO:0044451	nucleoplasm part	CC	1	3	67	483	0.014925	0.006211	0.361733	0.333333
GO:0009152	purine ribonucleotide biosynthetic process	BP	1	3	67	483	0.014925	0.006211	0.361733	0.333333

GO:0009593	detection of chemical stimulus	BP	1	3	67	483	0.014925	0.006211	0.361733	0.333333
GO:0045892	negative regulation of transcription, DNA-templated	BP	1	3	67	483	0.014925	0.006211	0.361733	0.333333
GO:0009259	ribonucleotide metabolic process	BP	1	3	67	483	0.014925	0.006211	0.361733	0.333333
GO:0009199	ribonucleoside triphosphate metabolic process	BP	1	3	67	483	0.014925	0.006211	0.361733	0.333333
GO:0002009	morphogenesis of an epithelium	BP	1	3	67	483	0.014925	0.006211	0.361733	0.333333
GO:0009166	nucleotide catabolic process	BP	1	3	67	483	0.014925	0.006211	0.361733	0.333333
GO:0008064	regulation of actin polymerization or depolymerization	BP	1	3	67	483	0.014925	0.006211	0.361733	0.333333
GO:0051052	regulation of DNA metabolic process	BP	1	3	67	483	0.014925	0.006211	0.361733	0.333333
GO:0061919	process utilizing autophagic mechanism	BP	1	3	67	483	0.014925	0.006211	0.361733	0.333333
GO:0051090	regulation of DNA-binding transcription factor activity	BP	1	3	67	483	0.014925	0.006211	0.361733	0.333333
GO:0046034	ATP metabolic process	BP	1	3	67	483	0.014925	0.006211	0.361733	0.333333
GO:0006163	purine nucleotide metabolic process	BP	1	3	67	483	0.014925	0.006211	0.361733	0.333333
GO:0001772	immunological synapse	CC	1	3	67	483	0.014925	0.006211	0.361733	0.333333
GO:0050912	detection of chemical stimulus involved in sensory perception of ta	BP	1	3	67	483	0.014925	0.006211	0.361733	0.333333
GO:0051788	response to misfolded protein	BP	1	3	67	483	0.014925	0.006211	0.361733	0.333333
GO:0006826	iron ion transport	BP	1	3	67	483	0.014925	0.006211	0.361733	0.333333
GO:0009117	nucleotide metabolic process	BP	1	3	67	483	0.014925	0.006211	0.361733	0.333333
GO:0072521	purine-containing compound metabolic process	BP	1	3	67	483	0.014925	0.006211	0.361733	0.333333
GO:0019362	pyridine nucleotide metabolic process	BP	1	3	67	483	0.014925	0.006211	0.361733	0.333333
GO:0031532	actin cytoskeleton reorganization	BP	1	3	67	483	0.014925	0.006211	0.361733	0.333333
GO:0032434	regulation of proteasomal ubiquitin-dependent protein catabolic pr	BP	1	3	67	483	0.014925	0.006211	0.361733	0.333333
GO:0008360	regulation of cell shape	BP	1	3	67	483	0.014925	0.006211	0.361733	0.333333
GO:1905953	negative regulation of lipid localization	BP	1	3	67	483	0.014925	0.006211	0.361733	0.333333
GO:0031099	regeneration	BP	1	3	67	483	0.014925	0.006211	0.361733	0.333333
GO:0090077	foam cell differentiation	BP	1	3	67	483	0.014925	0.006211	0.361733	0.333333
GO:0009168	purine ribonucleoside monophosphate biosynthetic process	BP	1	3	67	483	0.014925	0.006211	0.361733	0.333333
GO:0009260	ribonucleotide biosynthetic process	BP	1	3	67	483	0.014925	0.006211	0.361733	0.333333
GO:1902679	negative regulation of RNA biosynthetic process	BP	1	3	67	483	0.014925	0.006211	0.361733	0.333333
GO:0050766	positive regulation of phagocytosis	BP	1	3	67	483	0.014925	0.006211	0.361733	0.333333
GO:0072524	pyridine-containing compound metabolic process	BP	1	3	67	483	0.014925	0.006211	0.361733	0.333333
GO:0019915	lipid storage	BP	1	3	67	483	0.014925	0.006211	0.361733	0.333333
GO:0044431	Golgi apparatus part	CC	1	3	67	483	0.014925	0.006211	0.361733	0.333333

GO:0006754	ATP biosynthetic process	BP	1	3	67	483	0.014925	0.006211	0.361733	0.333333
GO:0044262	cellular carbohydrate metabolic process	BP	1	3	67	483	0.014925	0.006211	0.361733	0.333333
GO:0098791	Golgi subcompartment	CC	1	3	67	483	0.014925	0.006211	0.361733	0.333333
GO:0019693	ribose phosphate metabolic process	BP	1	3	67	483	0.014925	0.006211	0.361733	0.333333
GO:0005504	fatty acid binding	MF	1	3	67	483	0.014925	0.006211	0.361733	0.333333
GO:0000041	transition metal ion transport	BP	1	3	67	483	0.014925	0.006211	0.361733	0.333333
GO:0016055	Wnt signaling pathway	BP	1	3	67	483	0.014925	0.006211	0.361733	0.333333
GO:0042692	muscle cell differentiation	BP	1	3	67	483	0.014925	0.006211	0.361733	0.333333
GO:0050909	sensory perception of taste	BP	1	3	67	483	0.014925	0.006211	0.361733	0.333333
GO:0001580	detection of chemical stimulus involved in sensory perception of bi	BP	1	3	67	483	0.014925	0.006211	0.361733	0.333333
GO:0001894	tissue homeostasis	BP	1	3	67	483	0.014925	0.006211	0.361733	0.333333
GO:1903507	negative regulation of nucleic acid-templated transcription	BP	1	3	67	483	0.014925	0.006211	0.361733	0.333333
GO:0030139	endocytic vesicle	CC	1	3	67	483	0.014925	0.006211	0.361733	0.333333
GO:0009123	nucleoside monophosphate metabolic process	BP	1	3	67	483	0.014925	0.006211	0.361733	0.333333
GO:0009150	purine ribonucleotide metabolic process	BP	1	3	67	483	0.014925	0.006211	0.361733	0.333333
GO:0042102	positive regulation of T cell proliferation	BP	1	3	67	483	0.014925	0.006211	0.361733	0.333333
GO:0044433	cytoplasmic vesicle part	CC	1	3	67	483	0.014925	0.006211	0.361733	0.333333
GO:0030832	regulation of actin filament length	BP	1	3	67	483	0.014925	0.006211	0.361733	0.333333
GO:2000116	regulation of cysteine-type endopeptidase activity	BP	1	3	67	483	0.014925	0.006211	0.361733	0.333333
GO:0009142	nucleoside triphosphate biosynthetic process	BP	1	3	67	483	0.014925	0.006211	0.361733	0.333333
GO:1903319	positive regulation of protein maturation	BP	1	3	67	483	0.014925	0.006211	0.361733	0.333333
GO:0009126	purine nucleoside monophosphate metabolic process	BP	1	3	67	483	0.014925	0.006211	0.361733	0.333333
GO:0035966	response to topologically incorrect protein	BP	1	3	67	483	0.014925	0.006211	0.361733	0.333333
GO:0009167	purine ribonucleoside monophosphate metabolic process	BP	1	3	67	483	0.014925	0.006211	0.361733	0.333333
GO:1901293	nucleoside phosphate biosynthetic process	BP	1	3	67	483	0.014925	0.006211	0.361733	0.333333
GO:0006091	generation of precursor metabolites and energy	BP	1	3	67	483	0.014925	0.006211	0.361733	0.333333
GO:0034976	response to endoplasmic reticulum stress	BP	1	3	67	483	0.014925	0.006211	0.361733	0.333333
GO:1903363	negative regulation of cellular protein catabolic process	BP	1	3	67	483	0.014925	0.006211	0.361733	0.333333
GO:0010742	macrophage derived foam cell differentiation	BP	1	3	67	483	0.014925	0.006211	0.361733	0.333333
GO:0009206	purine ribonucleoside triphosphate biosynthetic process	BP	1	3	67	483	0.014925	0.006211	0.361733	0.333333
GO:0016324	apical plasma membrane	CC	1	3	67	483	0.014925	0.006211	0.361733	0.333333
GO:0045934	negative regulation of nucleobase-containing compound metabolic	BP	1	3	67	483	0.014925	0.006211	0.361733	0.333333

GO:0009205	purine ribonucleoside triphosphate metabolic process	BP	1	3	67	483	0.014925	0.006211	0.361733	0.333333
GO:0030863	cortical cytoskeleton	CC	1	3	67	483	0.014925	0.006211	0.361733	0.333333
GO:0090304	nucleic acid metabolic process	BP	4	22	67	483	0.059701	0.045549	0.364327	0.181818
GO:0043231	intracellular membrane-bounded organelle	CC	12	77	67	483	0.179104	0.15942	0.374038	0.155844
GO:0032555	purine ribonucleotide binding	MF	5	29	67	483	0.074627	0.060041	0.374781	0.172414
GO:0032553	ribonucleotide binding	MF	5	29	67	483	0.074627	0.060041	0.374781	0.172414
GO:0035639	purine ribonucleoside triphosphate binding	MF	5	29	67	483	0.074627	0.060041	0.374781	0.172414
GO:0017076	purine nucleotide binding	MF	5	29	67	483	0.074627	0.060041	0.374781	0.172414
GO:0010647	positive regulation of cell communication	BP	3	16	67	483	0.044776	0.033126	0.386979	0.1875
GO:0023056	positive regulation of signaling	BP	3	16	67	483	0.044776	0.033126	0.386979	0.1875
GO:0005634	nucleus	CC	8	50	67	483	0.119403	0.10352	0.388761	0.16
GO:0080090	regulation of primary metabolic process	BP	9	57	67	483	0.134328	0.118012	0.390781	0.157895
GO:0044444	cytoplasmic part	CC	12	78	67	483	0.179104	0.161491	0.393348	0.153846
GO:0005488	binding	MF	32	220	67	483	0.477612	0.455487	0.396618	0.145455
GO:0016310	phosphorylation	BP	4	23	67	483	0.059701	0.047619	0.398532	0.173913
GO:0044428	nuclear part	CC	4	23	67	483	0.059701	0.047619	0.398532	0.173913
GO:0051246	regulation of protein metabolic process	BP	7	44	67	483	0.104478	0.091097	0.410924	0.159091
GO:0036094	small molecule binding	MF	8	51	67	483	0.119403	0.10559	0.412267	0.156863
GO:0044267	cellular protein metabolic process	BP	10	65	67	483	0.149254	0.134576	0.413222	0.153846
GO:0045202	synapse	CC	2	10	67	483	0.029851	0.020704	0.414573	0.2
GO:0022414	reproductive process	BP	2	10	67	483	0.029851	0.020704	0.414573	0.2
GO:0000003	reproduction	BP	2	10	67	483	0.029851	0.020704	0.414573	0.2
GO:0019904	protein domain specific binding	MF	2	10	67	483	0.029851	0.020704	0.414573	0.2
GO:0008270	zinc ion binding	MF	2	10	67	483	0.029851	0.020704	0.414573	0.2
GO:0045321	leukocyte activation	BP	2	10	67	483	0.029851	0.020704	0.414573	0.2
GO:0007017	microtubule-based process	BP	2	10	67	483	0.029851	0.020704	0.414573	0.2
GO:0005198	structural molecule activity	MF	3	17	67	483	0.044776	0.035197	0.427304	0.176471
GO:2001141	regulation of RNA biosynthetic process	BP	3	17	67	483	0.044776	0.035197	0.427304	0.176471
GO:0006355	regulation of transcription, DNA-templated	BP	3	17	67	483	0.044776	0.035197	0.427304	0.176471
GO:0030162	regulation of proteolysis	BP	3	17	67	483	0.044776	0.035197	0.427304	0.176471
GO:1903506	regulation of nucleic acid-templated transcription	BP	3	17	67	483	0.044776	0.035197	0.427304	0.176471
GO:0044237	cellular metabolic process	BP	15	101	67	483	0.223881	0.20911	0.428348	0.148515

GO:0000166	nucleotide binding	MF	5	31	67	483	0.074627	0.064182	0.434846	0.16129
GO:1901265	nucleoside phosphate binding	MF	5	31	67	483	0.074627	0.064182	0.434846	0.16129
GO:0050867	positive regulation of cell activation	BP	1	4	67	483	0.014925	0.008282	0.450824	0.25
GO:0048729	tissue morphogenesis	BP	1	4	67	483	0.014925	0.008282	0.450824	0.25
GO:1902905	positive regulation of supramolecular fiber organization	BP	1	4	67	483	0.014925	0.008282	0.450824	0.25
GO:0044270	cellular nitrogen compound catabolic process	BP	1	4	67	483	0.014925	0.008282	0.450824	0.25
GO:0005730	nucleolus	CC	1	4	67	483	0.014925	0.008282	0.450824	0.25
GO:0051251	positive regulation of lymphocyte activation	BP	1	4	67	483	0.014925	0.008282	0.450824	0.25
GO:0042129	regulation of T cell proliferation	BP	1	4	67	483	0.014925	0.008282	0.450824	0.25
GO:0031647	regulation of protein stability	BP	1	4	67	483	0.014925	0.008282	0.450824	0.25
GO:0050750	low-density lipoprotein particle receptor binding	MF	1	4	67	483	0.014925	0.008282	0.450824	0.25
GO:1903037	regulation of leukocyte cell-cell adhesion	BP	1	4	67	483	0.014925	0.008282	0.450824	0.25
GO:0043177	organic acid binding	MF	1	4	67	483	0.014925	0.008282	0.450824	0.25
GO:0031032	actomyosin structure organization	BP	1	4	67	483	0.014925	0.008282	0.450824	0.25
GO:0050863	regulation of T cell activation	BP	1	4	67	483	0.014925	0.008282	0.450824	0.25
GO:0032677	regulation of interleukin-8 production	BP	1	4	67	483	0.014925	0.008282	0.450824	0.25
GO:0016049	cell growth	BP	1	4	67	483	0.014925	0.008282	0.450824	0.25
GO:0042177	negative regulation of protein catabolic process	BP	1	4	67	483	0.014925	0.008282	0.450824	0.25
GO:0019439	aromatic compound catabolic process	BP	1	4	67	483	0.014925	0.008282	0.450824	0.25
GO:0015318	inorganic molecular entity transmembrane transporter activity	MF	1	4	67	483	0.014925	0.008282	0.450824	0.25
GO:0043542	endothelial cell migration	BP	1	4	67	483	0.014925	0.008282	0.450824	0.25
GO:0001701	in utero embryonic development	BP	1	4	67	483	0.014925	0.008282	0.450824	0.25
GO:0044427	chromosomal part	CC	1	4	67	483	0.014925	0.008282	0.450824	0.25
GO:0046700	heterocycle catabolic process	BP	1	4	67	483	0.014925	0.008282	0.450824	0.25
GO:0050870	positive regulation of T cell activation	BP	1	4	67	483	0.014925	0.008282	0.450824	0.25
GO:0006006	glucose metabolic process	BP	1	4	67	483	0.014925	0.008282	0.450824	0.25
GO:0006974	cellular response to DNA damage stimulus	BP	1	4	67	483	0.014925	0.008282	0.450824	0.25
GO:0032535	regulation of cellular component size	BP	1	4	67	483	0.014925	0.008282	0.450824	0.25
GO:0110053	regulation of actin filament organization	BP	1	4	67	483	0.014925	0.008282	0.450824	0.25
GO:0033700	phospholipid efflux	BP	1	4	67	483	0.014925	0.008282	0.450824	0.25
GO:0001655	urogenital system development	BP	1	4	67	483	0.014925	0.008282	0.450824	0.25
GO:0035690	cellular response to drug	BP	1	4	67	483	0.014925	0.008282	0.450824	0.25

GO:0050906	detection of stimulus involved in sensory perception	BP	1	4	67	483	0.014925	0.008282	0.450824	0.25
GO:0002696	positive regulation of leukocyte activation	BP	1	4	67	483	0.014925	0.008282	0.450824	0.25
GO:0005996	monosaccharide metabolic process	BP	1	4	67	483	0.014925	0.008282	0.450824	0.25
GO:0022402	cell cycle process	BP	1	4	67	483	0.014925	0.008282	0.450824	0.25
GO:0097746	regulation of blood vessel diameter	BP	1	4	67	483	0.014925	0.008282	0.450824	0.25
GO:0015748	organophosphate ester transport	BP	1	4	67	483	0.014925	0.008282	0.450824	0.25
GO:0050670	regulation of lymphocyte proliferation	BP	1	4	67	483	0.014925	0.008282	0.450824	0.25
GO:0009792	embryo development ending in birth or egg hatching	BP	1	4	67	483	0.014925	0.008282	0.450824	0.25
GO:0043009	chordate embryonic development	BP	1	4	67	483	0.014925	0.008282	0.450824	0.25
GO:0005694	chromosome	CC	1	4	67	483	0.014925	0.008282	0.450824	0.25
GO:0061564	axon development	BP	1	4	67	483	0.014925	0.008282	0.450824	0.25
GO:0050821	protein stabilization	BP	1	4	67	483	0.014925	0.008282	0.450824	0.25
GO:0051260	protein homooligomerization	BP	1	4	67	483	0.014925	0.008282	0.450824	0.25
GO:0050662	coenzyme binding	MF	1	4	67	483	0.014925	0.008282	0.450824	0.25
GO:0005769	early endosome	CC	1	4	67	483	0.014925	0.008282	0.450824	0.25
GO:0001558	regulation of cell growth	BP	1	4	67	483	0.014925	0.008282	0.450824	0.25
GO:0050880	regulation of blood vessel size	BP	1	4	67	483	0.014925	0.008282	0.450824	0.25
GO:0007224	smoothened signaling pathway	BP	1	4	67	483	0.014925	0.008282	0.450824	0.25
GO:0055086	nucleobase-containing small molecule metabolic process	BP	1	4	67	483	0.014925	0.008282	0.450824	0.25
GO:0051259	protein complex oligomerization	BP	1	4	67	483	0.014925	0.008282	0.450824	0.25
GO:0034655	nucleobase-containing compound catabolic process	BP	1	4	67	483	0.014925	0.008282	0.450824	0.25
GO:0051495	positive regulation of cytoskeleton organization	BP	1	4	67	483	0.014925	0.008282	0.450824	0.25
GO:0015914	phospholipid transport	BP	1	4	67	483	0.014925	0.008282	0.450824	0.25
GO:0035296	regulation of tube diameter	BP	1	4	67	483	0.014925	0.008282	0.450824	0.25
GO:0043394	proteoglycan binding	MF	1	4	67	483	0.014925	0.008282	0.450824	0.25
GO:1901361	organic cyclic compound catabolic process	BP	1	4	67	483	0.014925	0.008282	0.450824	0.25
GO:0031406	carboxylic acid binding	MF	1	4	67	483	0.014925	0.008282	0.450824	0.25
GO:0019318	hexose metabolic process	BP	1	4	67	483	0.014925	0.008282	0.450824	0.25
GO:0043122	regulation of I-kappaB kinase/NF-kappaB signaling	BP	1	4	67	483	0.014925	0.008282	0.450824	0.25
GO:0003018	vascular process in circulatory system	BP	1	4	67	483	0.014925	0.008282	0.450824	0.25
GO:0050764	regulation of phagocytosis	BP	1	4	67	483	0.014925	0.008282	0.450824	0.25
GO:1903039	positive regulation of leukocyte cell-cell adhesion	BP	1	4	67	483	0.014925	0.008282	0.450824	0.25

GO:0090132	epithelium migration	BP	1	4	67	483	0.014925	0.008282	0.450824	0.25
GO:0035150	regulation of tube size	BP	1	4	67	483	0.014925	0.008282	0.450824	0.25
GO:0010631	epithelial cell migration	BP	1	4	67	483	0.014925	0.008282	0.450824	0.25
GO:0043933	protein-containing complex subunit organization	BP	5	32	67	483	0.074627	0.066253	0.464537	0.15625
GO:0032549	ribonucleoside binding	MF	2	11	67	483	0.029851	0.022774	0.465391	0.181818
GO:0030030	cell projection organization	BP	2	11	67	483	0.029851	0.022774	0.465391	0.181818
GO:0001882	nucleoside binding	MF	2	11	67	483	0.029851	0.022774	0.465391	0.181818
GO:0120036	plasma membrane bounded cell projection organization	BP	2	11	67	483	0.029851	0.022774	0.465391	0.181818
GO:1901135	carbohydrate derivative metabolic process	BP	2	11	67	483	0.029851	0.022774	0.465391	0.181818
GO:0071345	cellular response to cytokine stimulus	BP	2	11	67	483	0.029851	0.022774	0.465391	0.181818
GO:0005525	GTP binding	MF	2	11	67	483	0.029851	0.022774	0.465391	0.181818
GO:0015630	microtubule cytoskeleton	CC	2	11	67	483	0.029851	0.022774	0.465391	0.181818
GO:0019001	guanyl nucleotide binding	MF	2	11	67	483	0.029851	0.022774	0.465391	0.181818
GO:0032561	guanyl ribonucleotide binding	MF	2	11	67	483	0.029851	0.022774	0.465391	0.181818
GO:0001883	purine nucleoside binding	MF	2	11	67	483	0.029851	0.022774	0.465391	0.181818
GO:0032550	purine ribonucleoside binding	MF	2	11	67	483	0.029851	0.022774	0.465391	0.181818
GO:0044281	small molecule metabolic process	BP	4	25	67	483	0.059701	0.05176	0.465956	0.16
GO:0071310	cellular response to organic substance	BP	4	25	67	483	0.059701	0.05176	0.465956	0.16
GO:0006139	nucleobase-containing compound metabolic process	BP	4	25	67	483	0.059701	0.05176	0.465956	0.16
GO:0031981	nuclear lumen	CC	3	18	67	483	0.044776	0.037267	0.466729	0.166667
GO:0032559	adenyl ribonucleotide binding	MF	3	18	67	483	0.044776	0.037267	0.466729	0.166667
GO:0097659	nucleic acid-templated transcription	BP	3	18	67	483	0.044776	0.037267	0.466729	0.166667
GO:0005783	endoplasmic reticulum	CC	3	18	67	483	0.044776	0.037267	0.466729	0.166667
GO:0032774	RNA biosynthetic process	BP	3	18	67	483	0.044776	0.037267	0.466729	0.166667
GO:0030554	adenyl nucleotide binding	MF	3	18	67	483	0.044776	0.037267	0.466729	0.166667
GO:0006351	transcription, DNA-templated	BP	3	18	67	483	0.044776	0.037267	0.466729	0.166667
GO:0005524	ATP binding	MF	3	18	67	483	0.044776	0.037267	0.466729	0.166667
GO:0051252	regulation of RNA metabolic process	BP	3	18	67	483	0.044776	0.037267	0.466729	0.166667
GO:0043227	membrane-bounded organelle	CC	13	90	67	483	0.19403	0.186335	0.487596	0.144444
GO:0065009	regulation of molecular function	BP	4	26	67	483	0.059701	0.05383	0.498762	0.153846
GO:0031399	regulation of protein modification process	BP	3	19	67	483	0.044776	0.039337	0.504978	0.157895
GO:0016064	immunoglobulin mediated immune response	BP	2	12	67	483	0.029851	0.024845	0.513592	0.166667

GO:1902533	positive regulation of intracellular signal transduction	BP	2	12	67	483	0.029851	0.024845	0.513592	0.166667
GO:0099513	polymeric cytoskeletal fiber	CC	2	12	67	483	0.029851	0.024845	0.513592	0.166667
GO:0019724	B cell mediated immunity	BP	2	12	67	483	0.029851	0.024845	0.513592	0.166667
GO:0044403	symbiont process	BP	2	12	67	483	0.029851	0.024845	0.513592	0.166667
GO:0034097	response to cytokine	BP	2	12	67	483	0.029851	0.024845	0.513592	0.166667
GO:0007267	cell-cell signaling	BP	2	12	67	483	0.029851	0.024845	0.513592	0.166667
GO:0010557	positive regulation of macromolecule biosynthetic process	BP	2	12	67	483	0.029851	0.024845	0.513592	0.166667
GO:0050789	regulation of biological process	BP	19	135	67	483	0.283582	0.279503	0.52024	0.140741
GO:0050808	synapse organization	BP	1	5	67	483	0.014925	0.010352	0.52764	0.2
GO:0005938	cell cortex	CC	1	5	67	483	0.014925	0.010352	0.52764	0.2
GO:0043178	alcohol binding	MF	1	5	67	483	0.014925	0.010352	0.52764	0.2
GO:0040008	regulation of growth	BP	1	5	67	483	0.014925	0.010352	0.52764	0.2
GO:0046651	lymphocyte proliferation	BP	1	5	67	483	0.014925	0.010352	0.52764	0.2
GO:0031330	negative regulation of cellular catabolic process	BP	1	5	67	483	0.014925	0.010352	0.52764	0.2
GO:0044420	extracellular matrix component	CC	1	5	67	483	0.014925	0.010352	0.52764	0.2
GO:0043269	regulation of ion transport	BP	1	5	67	483	0.014925	0.010352	0.52764	0.2
GO:0010256	endomembrane system organization	BP	1	5	67	483	0.014925	0.010352	0.52764	0.2
GO:0002694	regulation of leukocyte activation	BP	1	5	67	483	0.014925	0.010352	0.52764	0.2
GO:0006887	exocytosis	BP	1	5	67	483	0.014925	0.010352	0.52764	0.2
GO:0008154	actin polymerization or depolymerization	BP	1	5	67	483	0.014925	0.010352	0.52764	0.2
GO:0043549	regulation of kinase activity	BP	1	5	67	483	0.014925	0.010352	0.52764	0.2
GO:0019901	protein kinase binding	MF	1	5	67	483	0.014925	0.010352	0.52764	0.2
GO:0042098	T cell proliferation	BP	1	5	67	483	0.014925	0.010352	0.52764	0.2
GO:0009896	positive regulation of catabolic process	BP	1	5	67	483	0.014925	0.010352	0.52764	0.2
GO:0090407	organophosphate biosynthetic process	BP	1	5	67	483	0.014925	0.010352	0.52764	0.2
GO:0032371	regulation of sterol transport	BP	1	5	67	483	0.014925	0.010352	0.52764	0.2
GO:0032637	interleukin-8 production	BP	1	5	67	483	0.014925	0.010352	0.52764	0.2
GO:0001667	ameboidal-type cell migration	BP	1	5	67	483	0.014925	0.010352	0.52764	0.2
GO:0061061	muscle structure development	BP	1	5	67	483	0.014925	0.010352	0.52764	0.2
GO:0048608	reproductive structure development	BP	1	5	67	483	0.014925	0.010352	0.52764	0.2
GO:0005833	hemoglobin complex	CC	1	5	67	483	0.014925	0.010352	0.52764	0.2
GO:0051087	chaperone binding	MF	1	5	67	483	0.014925	0.010352	0.52764	0.2

GO:0032374	regulation of cholesterol transport	BP	1	5	67	483	0.014925	0.010352	0.52764	0.2
GO:0090130	tissue migration	BP	1	5	67	483	0.014925	0.010352	0.52764	0.2
GO:2000113	negative regulation of cellular macromolecule biosynthetic process	BP	1	5	67	483	0.014925	0.010352	0.52764	0.2
GO:0032368	regulation of lipid transport	BP	1	5	67	483	0.014925	0.010352	0.52764	0.2
GO:1903827	regulation of cellular protein localization	BP	1	5	67	483	0.014925	0.010352	0.52764	0.2
GO:0022604	regulation of cell morphogenesis	BP	1	5	67	483	0.014925	0.010352	0.52764	0.2
GO:0061458	reproductive system development	BP	1	5	67	483	0.014925	0.010352	0.52764	0.2
GO:0007249	I-kappaB kinase/NF-kappaB signaling	BP	1	5	67	483	0.014925	0.010352	0.52764	0.2
GO:0140104	molecular carrier activity	MF	1	5	67	483	0.014925	0.010352	0.52764	0.2
GO:0006879	cellular iron ion homeostasis	BP	1	5	67	483	0.014925	0.010352	0.52764	0.2
GO:0051345	positive regulation of hydrolase activity	BP	1	5	67	483	0.014925	0.010352	0.52764	0.2
GO:0051338	regulation of transferase activity	BP	1	5	67	483	0.014925	0.010352	0.52764	0.2
GO:0010558	negative regulation of macromolecule biosynthetic process	BP	1	5	67	483	0.014925	0.010352	0.52764	0.2
GO:0045944	positive regulation of transcription by RNA polymerase II	BP	1	5	67	483	0.014925	0.010352	0.52764	0.2
GO:0046916	cellular transition metal ion homeostasis	BP	1	5	67	483	0.014925	0.010352	0.52764	0.2
GO:0060249	anatomical structure homeostasis	BP	1	5	67	483	0.014925	0.010352	0.52764	0.2
GO:0051249	regulation of lymphocyte activation	BP	1	5	67	483	0.014925	0.010352	0.52764	0.2
GO:0032956	regulation of actin cytoskeleton organization	BP	1	5	67	483	0.014925	0.010352	0.52764	0.2
GO:0042578	phosphoric ester hydrolase activity	MF	1	5	67	483	0.014925	0.010352	0.52764	0.2
GO:1902903	regulation of supramolecular fiber organization	BP	1	5	67	483	0.014925	0.010352	0.52764	0.2
GO:0050707	regulation of cytokine secretion	BP	1	5	67	483	0.014925	0.010352	0.52764	0.2
GO:0055072	iron ion homeostasis	BP	1	5	67	483	0.014925	0.010352	0.52764	0.2
GO:0045807	positive regulation of endocytosis	BP	1	5	67	483	0.014925	0.010352	0.52764	0.2
GO:0051493	regulation of cytoskeleton organization	BP	1	5	67	483	0.014925	0.010352	0.52764	0.2
GO:0055076	transition metal ion homeostasis	BP	1	5	67	483	0.014925	0.010352	0.52764	0.2
GO:0048871	multicellular organismal homeostasis	BP	1	5	67	483	0.014925	0.010352	0.52764	0.2
GO:0004888	transmembrane signaling receptor activity	MF	1	5	67	483	0.014925	0.010352	0.52764	0.2
GO:0007049	cell cycle	BP	1	5	67	483	0.014925	0.010352	0.52764	0.2
GO:0005344	oxygen carrier activity	MF	1	5	67	483	0.014925	0.010352	0.52764	0.2
GO:0008283	cell proliferation	BP	4	27	67	483	0.059701	0.055901	0.530734	0.148148
GO:0044430	cytoskeletal part	CC	3	20	67	483	0.044776	0.041408	0.54183	0.15
GO:0048523	negative regulation of cellular process	BP	8	57	67	483	0.119403	0.118012	0.551013	0.140351

GO:0034613	cellular protein localization	BP	2	13	67	483	0.029851	0.026915	0.558919	0.153846
GO:0070727	cellular macromolecule localization	BP	2	13	67	483	0.029851	0.026915	0.558919	0.153846
GO:0032880	regulation of protein localization	BP	2	13	67	483	0.029851	0.026915	0.558919	0.153846
GO:0001934	positive regulation of protein phosphorylation	BP	2	13	67	483	0.029851	0.026915	0.558919	0.153846
GO:0006811	ion transport	BP	2	13	67	483	0.029851	0.026915	0.558919	0.153846
GO:0044419	interspecies interaction between organisms	BP	2	13	67	483	0.029851	0.026915	0.558919	0.153846
GO:0044087	regulation of cellular component biogenesis	BP	2	13	67	483	0.029851	0.026915	0.558919	0.153846
GO:0009894	regulation of catabolic process	BP	2	13	67	483	0.029851	0.026915	0.558919	0.153846
GO:0009887	animal organ morphogenesis	BP	2	13	67	483	0.029851	0.026915	0.558919	0.153846
GO:0008284	positive regulation of cell proliferation	BP	2	13	67	483	0.029851	0.026915	0.558919	0.153846
GO:0032940	secretion by cell	BP	2	13	67	483	0.029851	0.026915	0.558919	0.153846
GO:0051649	establishment of localization in cell	BP	2	13	67	483	0.029851	0.026915	0.558919	0.153846
GO:0071702	organic substance transport	BP	6	43	67	483	0.089552	0.089027	0.567142	0.139535
GO:0016070	RNA metabolic process	BP	3	21	67	483	0.044776	0.043478	0.577117	0.142857
GO:0050790	regulation of catalytic activity	BP	3	21	67	483	0.044776	0.043478	0.577117	0.142857
GO:0001725	stress fiber	CC	1	6	67	483	0.014925	0.012422	0.59385	0.166667
GO:0044389	ubiquitin-like protein ligase binding	MF	1	6	67	483	0.014925	0.012422	0.59385	0.166667
GO:0050865	regulation of cell activation	BP	1	6	67	483	0.014925	0.012422	0.59385	0.166667
GO:0006875	cellular metal ion homeostasis	BP	1	6	67	483	0.014925	0.012422	0.59385	0.166667
GO:0010638	positive regulation of organelle organization	BP	1	6	67	483	0.014925	0.012422	0.59385	0.166667
GO:0030003	cellular cation homeostasis	BP	1	6	67	483	0.014925	0.012422	0.59385	0.166667
GO:0005506	iron ion binding	MF	1	6	67	483	0.014925	0.012422	0.59385	0.166667
GO:0002366	leukocyte activation involved in immune response	BP	1	6	67	483	0.014925	0.012422	0.59385	0.166667
GO:0042641	actomyosin	CC	1	6	67	483	0.014925	0.012422	0.59385	0.166667
GO:0022857	transmembrane transporter activity	MF	1	6	67	483	0.014925	0.012422	0.59385	0.166667
GO:0055080	cation homeostasis	BP	1	6	67	483	0.014925	0.012422	0.59385	0.166667
GO:0042493	response to drug	BP	1	6	67	483	0.014925	0.012422	0.59385	0.166667
GO:0002697	regulation of immune effector process	BP	1	6	67	483	0.014925	0.012422	0.59385	0.166667
GO:0044432	endoplasmic reticulum part	CC	1	6	67	483	0.014925	0.012422	0.59385	0.166667
GO:0099568	cytoplasmic region	CC	1	6	67	483	0.014925	0.012422	0.59385	0.166667
GO:0002285	lymphocyte activation involved in immune response	BP	1	6	67	483	0.014925	0.012422	0.59385	0.166667
GO:0009890	negative regulation of biosynthetic process	BP	1	6	67	483	0.014925	0.012422	0.59385	0.166667

GO:0006820	anion transport	BP	1	6	67	483	0.014925	0.012422	0.59385	0.166667
GO:0043235	receptor complex	CC	1	6	67	483	0.014925	0.012422	0.59385	0.166667
GO:0043068	positive regulation of programmed cell death	BP	1	6	67	483	0.014925	0.012422	0.59385	0.166667
GO:0031327	negative regulation of cellular biosynthetic process	BP	1	6	67	483	0.014925	0.012422	0.59385	0.166667
GO:0046434	organophosphate catabolic process	BP	1	6	67	483	0.014925	0.012422	0.59385	0.166667
GO:0006812	cation transport	BP	1	6	67	483	0.014925	0.012422	0.59385	0.166667
GO:0070325	lipoprotein particle receptor binding	MF	1	6	67	483	0.014925	0.012422	0.59385	0.166667
GO:0003006	developmental process involved in reproduction	BP	1	6	67	483	0.014925	0.012422	0.59385	0.166667
GO:0052548	regulation of endopeptidase activity	BP	1	6	67	483	0.014925	0.012422	0.59385	0.166667
GO:0022409	positive regulation of cell-cell adhesion	BP	1	6	67	483	0.014925	0.012422	0.59385	0.166667
GO:0060341	regulation of cellular localization	BP	1	6	67	483	0.014925	0.012422	0.59385	0.166667
GO:0043065	positive regulation of apoptotic process	BP	1	6	67	483	0.014925	0.012422	0.59385	0.166667
GO:0050877	nervous system process	BP	1	6	67	483	0.014925	0.012422	0.59385	0.166667
GO:0051606	detection of stimulus	BP	1	6	67	483	0.014925	0.012422	0.59385	0.166667
GO:0019900	kinase binding	MF	1	6	67	483	0.014925	0.012422	0.59385	0.166667
GO:0031625	ubiquitin protein ligase binding	MF	1	6	67	483	0.014925	0.012422	0.59385	0.166667
GO:0098805	whole membrane	CC	1	6	67	483	0.014925	0.012422	0.59385	0.166667
GO:0097517	contractile actin filament bundle	CC	1	6	67	483	0.014925	0.012422	0.59385	0.166667
GO:0031175	neuron projection development	BP	1	6	67	483	0.014925	0.012422	0.59385	0.166667
GO:0055065	metal ion homeostasis	BP	1	6	67	483	0.014925	0.012422	0.59385	0.166667
GO:0050663	cytokine secretion	BP	1	6	67	483	0.014925	0.012422	0.59385	0.166667
GO:0098771	inorganic ion homeostasis	BP	1	6	67	483	0.014925	0.012422	0.59385	0.166667
GO:0032432	actin filament bundle	CC	1	6	67	483	0.014925	0.012422	0.59385	0.166667
GO:0007600	sensory perception	BP	1	6	67	483	0.014925	0.012422	0.59385	0.166667
GO:0006873	cellular ion homeostasis	BP	1	6	67	483	0.014925	0.012422	0.59385	0.166667
GO:0015711	organic anion transport	BP	1	6	67	483	0.014925	0.012422	0.59385	0.166667
GO:0050673	epithelial cell proliferation	BP	1	6	67	483	0.014925	0.012422	0.59385	0.166667
GO:0030001	metal ion transport	BP	1	6	67	483	0.014925	0.012422	0.59385	0.166667
GO:0002263	cell activation involved in immune response	BP	1	6	67	483	0.014925	0.012422	0.59385	0.166667
GO:0038024	cargo receptor activity	MF	1	6	67	483	0.014925	0.012422	0.59385	0.166667
GO:0016788	hydrolase activity, acting on ester bonds	MF	2	14	67	483	0.029851	0.028986	0.601232	0.142857
GO:0002460	adaptive immune response based on somatic recombination of imm	BP	2	14	67	483	0.029851	0.028986	0.601232	0.142857

GO:0002449	lymphocyte mediated immunity	BP	2	14	67	483	0.029851	0.028986	0.601232	0.142857
GO:0002443	leukocyte mediated immunity	BP	2	14	67	483	0.029851	0.028986	0.601232	0.142857
GO:0048037	cofactor binding	MF	2	14	67	483	0.029851	0.028986	0.601232	0.142857
GO:0008047	enzyme activator activity	MF	1	7	67	483	0.014925	0.014493	0.650898	0.142857
GO:1903317	regulation of protein maturation	BP	1	7	67	483	0.014925	0.014493	0.650898	0.142857
GO:0030100	regulation of endocytosis	BP	1	7	67	483	0.014925	0.014493	0.650898	0.142857
GO:0005874	microtubule	CC	1	7	67	483	0.014925	0.014493	0.650898	0.142857
GO:0002020	protease binding	MF	1	7	67	483	0.014925	0.014493	0.650898	0.142857
GO:1903508	positive regulation of nucleic acid-templated transcription	BP	1	7	67	483	0.014925	0.014493	0.650898	0.142857
GO:0050801	ion homeostasis	BP	1	7	67	483	0.014925	0.014493	0.650898	0.142857
GO:0051254	positive regulation of RNA metabolic process	BP	1	7	67	483	0.014925	0.014493	0.650898	0.142857
GO:0052547	regulation of peptidase activity	BP	1	7	67	483	0.014925	0.014493	0.650898	0.142857
GO:0007159	leukocyte cell-cell adhesion	BP	1	7	67	483	0.014925	0.014493	0.650898	0.142857
GO:0045893	positive regulation of transcription, DNA-templated	BP	1	7	67	483	0.014925	0.014493	0.650898	0.142857
GO:0020037	heme binding	MF	1	7	67	483	0.014925	0.014493	0.650898	0.142857
GO:0055082	cellular chemical homeostasis	BP	1	7	67	483	0.014925	0.014493	0.650898	0.142857
GO:0008015	blood circulation	BP	1	7	67	483	0.014925	0.014493	0.650898	0.142857
GO:0005604	basement membrane	CC	1	7	67	483	0.014925	0.014493	0.650898	0.142857
GO:0010942	positive regulation of cell death	BP	1	7	67	483	0.014925	0.014493	0.650898	0.142857
GO:0009895	negative regulation of catabolic process	BP	1	7	67	483	0.014925	0.014493	0.650898	0.142857
GO:0003013	circulatory system process	BP	1	7	67	483	0.014925	0.014493	0.650898	0.142857
GO:0070613	regulation of protein processing	BP	1	7	67	483	0.014925	0.014493	0.650898	0.142857
GO:0005319	lipid transporter activity	MF	1	7	67	483	0.014925	0.014493	0.650898	0.142857
GO:0043410	positive regulation of MAPK cascade	BP	1	7	67	483	0.014925	0.014493	0.650898	0.142857
GO:0043161	proteasome-mediated ubiquitin-dependent protein catabolic proces	BP	1	7	67	483	0.014925	0.014493	0.650898	0.142857
GO:0000904	cell morphogenesis involved in differentiation	BP	1	7	67	483	0.014925	0.014493	0.650898	0.142857
GO:1902680	positive regulation of RNA biosynthetic process	BP	1	7	67	483	0.014925	0.014493	0.650898	0.142857
GO:0033036	macromolecule localization	BP	6	44	67	483	0.089552	0.091097	0.591808	0.136364
GO:0051716	cellular response to stimulus	BP	8	60	67	483	0.119403	0.124224	0.615858	0.133333
GO:0009057	macromolecule catabolic process	BP	4	30	67	483	0.059701	0.062112	0.620381	0.133333
GO:0005737	cytoplasm	CC	14	105	67	483	0.208955	0.217391	0.625507	0.133333
GO:0065007	biological regulation	BP	21	157	67	483	0.313433	0.325052	0.636275	0.133758

GO:0005509	calcium ion binding	MF	6	46	67	483	0.089552	0.095238	0.638972	0.130435
GO:0030036	actin cytoskeleton organization	BP	2	15	67	483	0.029851	0.031056	0.640484	0.133333
GO:0098796	membrane protein complex	CC	2	15	67	483	0.029851	0.031056	0.640484	0.133333
GO:0030029	actin filament-based process	BP	2	15	67	483	0.029851	0.031056	0.640484	0.133333
GO:0044093	positive regulation of molecular function	BP	2	15	67	483	0.029851	0.031056	0.640484	0.133333
GO:0070013	intracellular organelle lumen	CC	3	23	67	483	0.044776	0.047619	0.642543	0.130435
GO:0065003	protein-containing complex assembly	BP	3	23	67	483	0.044776	0.047619	0.642543	0.130435
GO:0046914	transition metal ion binding	MF	3	23	67	483	0.044776	0.047619	0.642543	0.130435
GO:0051603	proteolysis involved in cellular protein catabolic process	BP	3	23	67	483	0.044776	0.047619	0.642543	0.130435
GO:0031974	membrane-enclosed lumen	CC	3	23	67	483	0.044776	0.047619	0.642543	0.130435
GO:0043233	organelle lumen	CC	3	23	67	483	0.044776	0.047619	0.642543	0.130435
GO:0042127	regulation of cell proliferation	BP	3	23	67	483	0.044776	0.047619	0.642543	0.130435
GO:0016043	cellular component organization	BP	9	69	67	483	0.134328	0.142857	0.645993	0.130435
GO:0071840	cellular component organization or biogenesis	BP	9	69	67	483	0.134328	0.142857	0.645993	0.130435
GO:0010033	response to organic substance	BP	4	31	67	483	0.059701	0.064182	0.647867	0.129032
GO:0042221	response to chemical	BP	6	47	67	483	0.089552	0.097308	0.66137	0.12766
GO:0019538	protein metabolic process	BP	13	100	67	483	0.19403	0.207039	0.665074	0.13
GO:0044257	cellular protein catabolic process	BP	3	24	67	483	0.044776	0.049689	0.672552	0.125
GO:0070887	cellular response to chemical stimulus	BP	4	32	67	483	0.059701	0.066253	0.674057	0.125
GO:0006796	phosphate-containing compound metabolic process	BP	4	32	67	483	0.059701	0.066253	0.674057	0.125
GO:0046903	secretion	BP	2	16	67	483	0.029851	0.033126	0.676699	0.125
GO:0005654	nucleoplasm	CC	2	16	67	483	0.029851	0.033126	0.676699	0.125
GO:0003008	system process	BP	2	16	67	483	0.029851	0.033126	0.676699	0.125
GO:0098609	cell-cell adhesion	BP	2	16	67	483	0.029851	0.033126	0.676699	0.125
GO:0006508	proteolysis	BP	5	40	67	483	0.074627	0.082816	0.677915	0.125
GO:0048519	negative regulation of biological process	BP	8	64	67	483	0.119403	0.132505	0.694569	0.125
GO:0016787	hydrolase activity	MF	11	87	67	483	0.164179	0.180124	0.697553	0.126437
GO:0006793	phosphorus metabolic process	BP	4	33	67	483	0.059701	0.068323	0.69892	0.121212
GO:0007155	cell adhesion	BP	4	33	67	483	0.059701	0.068323	0.69892	0.121212
GO:0016125	sterol metabolic process	BP	1	8	67	483	0.014925	0.016563	0.700036	0.125
GO:1901698	response to nitrogen compound	BP	1	8	67	483	0.014925	0.016563	0.700036	0.125
GO:0046906	tetrapyrrole binding	MF	1	8	67	483	0.014925	0.016563	0.700036	0.125

GO:0031252	cell leading edge	CC	1	8	67	483	0.014925	0.016563	0.700036	0.125
GO:0010243	response to organonitrogen compound	BP	1	8	67	483	0.014925	0.016563	0.700036	0.125
GO:0006366	transcription by RNA polymerase II	BP	1	8	67	483	0.014925	0.016563	0.700036	0.125
GO:0009628	response to abiotic stimulus	BP	1	8	67	483	0.014925	0.016563	0.700036	0.125
GO:0060429	epithelium development	BP	1	8	67	483	0.014925	0.016563	0.700036	0.125
GO:0071363	cellular response to growth factor stimulus	BP	1	8	67	483	0.014925	0.016563	0.700036	0.125
GO:0006357	regulation of transcription by RNA polymerase II	BP	1	8	67	483	0.014925	0.016563	0.700036	0.125
GO:0070848	response to growth factor	BP	1	8	67	483	0.014925	0.016563	0.700036	0.125
GO:0022407	regulation of cell-cell adhesion	BP	1	8	67	483	0.014925	0.016563	0.700036	0.125
GO:0008203	cholesterol metabolic process	BP	1	8	67	483	0.014925	0.016563	0.700036	0.125
GO:0050708	regulation of protein secretion	BP	1	8	67	483	0.014925	0.016563	0.700036	0.125
GO:0033344	cholesterol efflux	BP	1	8	67	483	0.014925	0.016563	0.700036	0.125
GO:1902652	secondary alcohol metabolic process	BP	1	8	67	483	0.014925	0.016563	0.700036	0.125
GO:0050778	positive regulation of immune response	BP	3	25	67	483	0.044776	0.05176	0.700724	0.12
GO:0044265	cellular macromolecule catabolic process	BP	3	25	67	483	0.044776	0.05176	0.700724	0.12
GO:0002250	adaptive immune response	BP	3	25	67	483	0.044776	0.05176	0.700724	0.12
GO:0006996	organelle organization	BP	3	25	67	483	0.044776	0.05176	0.700724	0.12
GO:0031982	vesicle	CC	3	25	67	483	0.044776	0.05176	0.700724	0.12
GO:0008152	metabolic process	BP	19	147	67	483	0.283582	0.304348	0.702281	0.129252
GO:0071704	organic substance metabolic process	BP	18	140	67	483	0.268657	0.289855	0.707616	0.128571
GO:0062023	collagen-containing extracellular matrix	CC	2	17	67	483	0.029851	0.035197	0.709952	0.117647
GO:1902531	regulation of intracellular signal transduction	BP	2	17	67	483	0.029851	0.035197	0.709952	0.117647
GO:0001932	regulation of protein phosphorylation	BP	2	17	67	483	0.029851	0.035197	0.709952	0.117647
GO:0001775	cell activation	BP	2	17	67	483	0.029851	0.035197	0.709952	0.117647
GO:0012505	endomembrane system	CC	5	42	67	483	0.074627	0.086957	0.721837	0.119048
GO:0097367	carbohydrate derivative binding	MF	6	50	67	483	0.089552	0.10352	0.723421	0.12
GO:0030163	protein catabolic process	BP	3	26	67	483	0.044776	0.05383	0.727067	0.115385
GO:0007010	cytoskeleton organization	BP	2	18	67	483	0.029851	0.037267	0.740354	0.111111
GO:0000502	proteasome complex	CC	2	18	67	483	0.029851	0.037267	0.740354	0.111111
GO:1905369	endopeptidase complex	CC	2	18	67	483	0.029851	0.037267	0.740354	0.111111
GO:1905368	peptidase complex	CC	2	18	67	483	0.029851	0.037267	0.740354	0.111111
GO:0004175	endopeptidase activity	MF	5	43	67	483	0.074627	0.089027	0.742191	0.116279

GO:0005178	integrin binding	MF	1	9	67	483	0.014925	0.018634	0.742347	0.111111
GO:0005975	carbohydrate metabolic process	BP	1	9	67	483	0.014925	0.018634	0.742347	0.111111
GO:0002791	regulation of peptide secretion	BP	1	9	67	483	0.014925	0.018634	0.742347	0.111111
GO:0007015	actin filament organization	BP	1	9	67	483	0.014925	0.018634	0.742347	0.111111
GO:0040007	growth	BP	1	9	67	483	0.014925	0.018634	0.742347	0.111111
GO:0048666	neuron development	BP	1	9	67	483	0.014925	0.018634	0.742347	0.111111
GO:0007169	transmembrane receptor protein tyrosine kinase signaling pathway	BP	1	9	67	483	0.014925	0.018634	0.742347	0.111111
GO:0043085	positive regulation of catalytic activity	BP	1	9	67	483	0.014925	0.018634	0.742347	0.111111
GO:0060089	molecular transducer activity	MF	1	9	67	483	0.014925	0.018634	0.742347	0.111111
GO:0009306	protein secretion	BP	1	9	67	483	0.014925	0.018634	0.742347	0.111111
GO:0033043	regulation of organelle organization	BP	1	9	67	483	0.014925	0.018634	0.742347	0.111111
GO:0044283	small molecule biosynthetic process	BP	1	9	67	483	0.014925	0.018634	0.742347	0.111111
GO:0045861	negative regulation of proteolysis	BP	1	9	67	483	0.014925	0.018634	0.742347	0.111111
GO:0045785	positive regulation of cell adhesion	BP	1	9	67	483	0.014925	0.018634	0.742347	0.111111
GO:1903530	regulation of secretion by cell	BP	1	9	67	483	0.014925	0.018634	0.742347	0.111111
GO:0008202	steroid metabolic process	BP	1	9	67	483	0.014925	0.018634	0.742347	0.111111
GO:0045596	negative regulation of cell differentiation	BP	1	9	67	483	0.014925	0.018634	0.742347	0.111111
GO:0038023	signaling receptor activity	MF	1	9	67	483	0.014925	0.018634	0.742347	0.111111
GO:0005200	structural constituent of cytoskeleton	MF	1	9	67	483	0.014925	0.018634	0.742347	0.111111
GO:0009897	external side of plasma membrane	CC	1	9	67	483	0.014925	0.018634	0.742347	0.111111
GO:0048513	animal organ development	BP	4	35	67	483	0.059701	0.072464	0.744616	0.114286
GO:0022610	biological adhesion	BP	4	35	67	483	0.059701	0.072464	0.744616	0.114286
GO:0044459	plasma membrane part	CC	3	27	67	483	0.044776	0.055901	0.751608	0.111111
GO:0006807	nitrogen compound metabolic process	BP	15	121	67	483	0.223881	0.250518	0.753019	0.123967
GO:0046872	metal ion binding	MF	10	83	67	483	0.149254	0.171843	0.754198	0.120482
GO:0007154	cell communication	BP	6	52	67	483	0.089552	0.10766	0.760323	0.115385
GO:0048584	positive regulation of response to stimulus	BP	5	44	67	483	0.074627	0.091097	0.761471	0.113636
GO:0065008	regulation of biological quality	BP	8	68	67	483	0.119403	0.140787	0.762791	0.117647
GO:0099080	supramolecular complex	CC	2	19	67	483	0.029851	0.039337	0.768046	0.105263
GO:0006956	complement activation	BP	2	19	67	483	0.029851	0.039337	0.768046	0.105263
GO:0006468	protein phosphorylation	BP	2	19	67	483	0.029851	0.039337	0.768046	0.105263
GO:0031012	extracellular matrix	CC	2	19	67	483	0.029851	0.039337	0.768046	0.105263

GO:0099512	supramolecular fiber	CC	2	19	67	483	0.029851	0.039337	0.768046	0.105263
GO:0099081	supramolecular polymer	CC	2	19	67	483	0.029851	0.039337	0.768046	0.105263
GO:1902494	catalytic complex	CC	2	19	67	483	0.029851	0.039337	0.768046	0.105263
GO:0043169	cation binding	MF	10	84	67	483	0.149254	0.173913	0.76874	0.119048
GO:0002252	immune effector process	BP	3	28	67	483	0.044776	0.057971	0.774393	0.107143
GO:0009966	regulation of signal transduction	BP	3	28	67	483	0.044776	0.057971	0.774393	0.107143
GO:0030545	receptor regulator activity	MF	1	10	67	483	0.014925	0.020704	0.778766	0.1
GO:0002455	humoral immune response mediated by circulating immunoglobuli	BP	1	10	67	483	0.014925	0.020704	0.778766	0.1
GO:0010629	negative regulation of gene expression	BP	1	10	67	483	0.014925	0.020704	0.778766	0.1
GO:1901701	cellular response to oxygen-containing compound	BP	1	10	67	483	0.014925	0.020704	0.778766	0.1
GO:0048018	receptor ligand activity	MF	1	10	67	483	0.014925	0.020704	0.778766	0.1
GO:0008285	negative regulation of cell proliferation	BP	1	10	67	483	0.014925	0.020704	0.778766	0.1
GO:0005887	integral component of plasma membrane	CC	1	10	67	483	0.014925	0.020704	0.778766	0.1
GO:0006066	alcohol metabolic process	BP	1	10	67	483	0.014925	0.020704	0.778766	0.1
GO:0002790	peptide secretion	BP	1	10	67	483	0.014925	0.020704	0.778766	0.1
GO:0019814	immunoglobulin complex	CC	1	10	67	483	0.014925	0.020704	0.778766	0.1
GO:0006886	intracellular protein transport	BP	1	10	67	483	0.014925	0.020704	0.778766	0.1
GO:0051046	regulation of secretion	BP	1	10	67	483	0.014925	0.020704	0.778766	0.1
GO:1901615	organic hydroxy compound metabolic process	BP	1	10	67	483	0.014925	0.020704	0.778766	0.1
GO:0006958	complement activation, classical pathway	BP	1	10	67	483	0.014925	0.020704	0.778766	0.1
GO:0098552	side of membrane	CC	1	10	67	483	0.014925	0.020704	0.778766	0.1
GO:0019899	enzyme binding	MF	2	20	67	483	0.029851	0.041408	0.793181	0.1
GO:0050776	regulation of immune response	BP	3	29	67	483	0.044776	0.060041	0.795479	0.103448
GO:1901565	organonitrogen compound catabolic process	BP	3	29	67	483	0.044776	0.060041	0.795479	0.103448
GO:0044238	primary metabolic process	BP	15	125	67	483	0.223881	0.258799	0.801914	0.12
GO:0044248	cellular catabolic process	BP	4	38	67	483	0.059701	0.078675	0.803251	0.105263
GO:0043408	regulation of MAPK cascade	BP	1	11	67	483	0.014925	0.022774	0.810104	0.090909
GO:0031226	intrinsic component of plasma membrane	CC	1	11	67	483	0.014925	0.022774	0.810104	0.090909
GO:0060627	regulation of vesicle-mediated transport	BP	1	11	67	483	0.014925	0.022774	0.810104	0.090909
GO:0050839	cell adhesion molecule binding	MF	1	11	67	483	0.014925	0.022774	0.810104	0.090909
GO:0030301	cholesterol transport	BP	1	11	67	483	0.014925	0.022774	0.810104	0.090909
GO:0030182	neuron differentiation	BP	1	11	67	483	0.014925	0.022774	0.810104	0.090909

GO:0019637	organophosphate metabolic process	BP	1	11	67	483	0.014925	0.022774	0.810104	0.090909
GO:0000902	cell morphogenesis	BP	1	11	67	483	0.014925	0.022774	0.810104	0.090909
GO:0023014	signal transduction by protein phosphorylation	BP	1	11	67	483	0.014925	0.022774	0.810104	0.090909
GO:0009790	embryo development	BP	1	11	67	483	0.014925	0.022774	0.810104	0.090909
GO:0120025	plasma membrane bounded cell projection	CC	1	11	67	483	0.014925	0.022774	0.810104	0.090909
GO:0015918	sterol transport	BP	1	11	67	483	0.014925	0.022774	0.810104	0.090909
GO:0042995	cell projection	CC	1	11	67	483	0.014925	0.022774	0.810104	0.090909
GO:0015850	organic hydroxy compound transport	BP	1	11	67	483	0.014925	0.022774	0.810104	0.090909
GO:0000165	MAPK cascade	BP	1	11	67	483	0.014925	0.022774	0.810104	0.090909
GO:0051050	positive regulation of transport	BP	1	11	67	483	0.014925	0.022774	0.810104	0.090909
GO:0043167	ion binding	MF	15	126	67	483	0.223881	0.26087	0.813058	0.119048
GO:0031324	negative regulation of cellular metabolic process	BP	2	21	67	483	0.029851	0.043478	0.815924	0.095238
GO:0097708	intracellular vesicle	CC	2	21	67	483	0.029851	0.043478	0.815924	0.095238
GO:0010605	negative regulation of macromolecule metabolic process	BP	2	21	67	483	0.029851	0.043478	0.815924	0.095238
GO:0031410	cytoplasmic vesicle	CC	2	21	67	483	0.029851	0.043478	0.815924	0.095238
GO:0051172	negative regulation of nitrogen compound metabolic process	BP	2	21	67	483	0.029851	0.043478	0.815924	0.095238
GO:0004252	serine-type endopeptidase activity	MF	2	21	67	483	0.029851	0.043478	0.815924	0.095238
GO:1901564	organonitrogen compound metabolic process	BP	13	111	67	483	0.19403	0.229814	0.81673	0.117117
GO:0043168	anion binding	MF	6	56	67	483	0.089552	0.115942	0.823373	0.107143
GO:0008289	lipid binding	MF	3	31	67	483	0.044776	0.064182	0.832835	0.096774
GO:0010646	regulation of cell communication	BP	3	31	67	483	0.044776	0.064182	0.832835	0.096774
GO:0023051	regulation of signaling	BP	3	31	67	483	0.044776	0.064182	0.832835	0.096774
GO:0002684	positive regulation of immune system process	BP	3	31	67	483	0.044776	0.064182	0.832835	0.096774
GO:0008236	serine-type peptidase activity	MF	2	22	67	483	0.029851	0.045549	0.836445	0.090909
GO:0002253	activation of immune response	BP	2	22	67	483	0.029851	0.045549	0.836445	0.090909
GO:0017171	serine hydrolase activity	MF	2	22	67	483	0.029851	0.045549	0.836445	0.090909
GO:0006810	transport	BP	6	57	67	483	0.089552	0.118012	0.836966	0.105263
GO:0051234	establishment of localization	BP	6	57	67	483	0.089552	0.118012	0.836966	0.105263
GO:0009719	response to endogenous stimulus	BP	1	12	67	483	0.014925	0.024845	0.837059	0.083333
GO:0046907	intracellular transport	BP	1	12	67	483	0.014925	0.024845	0.837059	0.083333
GO:0051223	regulation of protein transport	BP	1	12	67	483	0.014925	0.024845	0.837059	0.083333
GO:0098797	plasma membrane protein complex	CC	1	12	67	483	0.014925	0.024845	0.837059	0.083333

GO:0071495	cellular response to endogenous stimulus	BP	1	12	67	483	0.014925	0.024845	0.837059	0.083333
GO:0051604	protein maturation	BP	1	12	67	483	0.014925	0.024845	0.837059	0.083333
GO:0016485	protein processing	BP	1	12	67	483	0.014925	0.024845	0.837059	0.083333
GO:0005215	transporter activity	MF	1	12	67	483	0.014925	0.024845	0.837059	0.083333
GO:0019725	cellular homeostasis	BP	1	12	67	483	0.014925	0.024845	0.837059	0.083333
GO:0070201	regulation of establishment of protein localization	BP	1	12	67	483	0.014925	0.024845	0.837059	0.083333
GO:0070011	peptidase activity, acting on L-amino acid peptides	MF	5	49	67	483	0.074627	0.101449	0.842238	0.102041
GO:0009611	response to wounding	BP	3	32	67	483	0.044776	0.066253	0.84926	0.09375
GO:0051704	multi-organism process	BP	3	32	67	483	0.044776	0.066253	0.84926	0.09375
GO:0048468	cell development	BP	2	23	67	483	0.029851	0.047619	0.854912	0.086957
GO:0023052	signaling	BP	5	50	67	483	0.074627	0.10352	0.85544	0.1
GO:0007167	enzyme linked receptor protein signaling pathway	BP	1	13	67	483	0.014925	0.026915	0.860238	0.076923
GO:0061041	regulation of wound healing	BP	1	13	67	483	0.014925	0.026915	0.860238	0.076923
GO:1901700	response to oxygen-containing compound	BP	1	13	67	483	0.014925	0.026915	0.860238	0.076923
GO:0019752	carboxylic acid metabolic process	BP	1	13	67	483	0.014925	0.026915	0.860238	0.076923
GO:1903034	regulation of response to wounding	BP	1	13	67	483	0.014925	0.026915	0.860238	0.076923
GO:0006511	ubiquitin-dependent protein catabolic process	BP	1	13	67	483	0.014925	0.026915	0.860238	0.076923
GO:0090087	regulation of peptide transport	BP	1	13	67	483	0.014925	0.026915	0.860238	0.076923
GO:0032989	cellular component morphogenesis	BP	1	13	67	483	0.014925	0.026915	0.860238	0.076923
GO:0050793	regulation of developmental process	BP	3	33	67	483	0.044776	0.068323	0.864294	0.090909
GO:0030154	cell differentiation	BP	4	42	67	483	0.059701	0.086957	0.864319	0.095238
GO:0010876	lipid localization	BP	2	24	67	483	0.029851	0.049689	0.87149	0.083333
GO:0051049	regulation of transport	BP	2	24	67	483	0.029851	0.049689	0.87149	0.083333
GO:0009892	negative regulation of metabolic process	BP	2	24	67	483	0.029851	0.049689	0.87149	0.083333
GO:1901575	organic substance catabolic process	BP	4	43	67	483	0.059701	0.089027	0.876845	0.093023
GO:0042157	lipoprotein metabolic process	BP	1	14	67	483	0.014925	0.028986	0.880161	0.071429
GO:0030155	regulation of cell adhesion	BP	1	14	67	483	0.014925	0.028986	0.880161	0.071429
GO:0051093	negative regulation of developmental process	BP	1	14	67	483	0.014925	0.028986	0.880161	0.071429
GO:0019941	modification-dependent protein catabolic process	BP	1	14	67	483	0.014925	0.028986	0.880161	0.071429
GO:0043632	modification-dependent macromolecule catabolic process	BP	1	14	67	483	0.014925	0.028986	0.880161	0.071429
GO:0006082	organic acid metabolic process	BP	1	14	67	483	0.014925	0.028986	0.880161	0.071429
GO:0097435	supramolecular fiber organization	BP	1	14	67	483	0.014925	0.028986	0.880161	0.071429

GO:0043436	oxoacid metabolic process	BP	1	14	67	483	0.014925	0.028986	0.880161	0.071429
GO:0035556	intracellular signal transduction	BP	2	25	67	483	0.029851	0.05176	0.88634	0.08
GO:0005102	signaling receptor binding	MF	4	44	67	483	0.059701	0.091097	0.888382	0.090909
GO:0009987	cellular process	BP	21	180	67	483	0.313433	0.372671	0.889052	0.116667
GO:0008233	peptidase activity	MF	5	53	67	483	0.074627	0.109731	0.88976	0.09434
GO:0009986	cell surface	CC	1	15	67	483	0.014925	0.031056	0.897281	0.066667
GO:0032269	negative regulation of cellular protein metabolic process	BP	1	15	67	483	0.014925	0.031056	0.897281	0.066667
GO:0001817	regulation of cytokine production	BP	1	15	67	483	0.014925	0.031056	0.897281	0.066667
GO:0051336	regulation of hydrolase activity	BP	1	15	67	483	0.014925	0.031056	0.897281	0.066667
GO:0048699	generation of neurons	BP	1	15	67	483	0.014925	0.031056	0.897281	0.066667
GO:0048869	cellular developmental process	BP	4	45	67	483	0.059701	0.093168	0.898987	0.088889
GO:0009056	catabolic process	BP	4	45	67	483	0.059701	0.093168	0.898987	0.088889
GO:0072376	protein activation cascade	BP	2	26	67	483	0.029851	0.05383	0.899613	0.076923
GO:0003824	catalytic activity	MF	13	121	67	483	0.19403	0.250518	0.906082	0.107438
GO:0048583	regulation of response to stimulus	BP	6	64	67	483	0.089552	0.132505	0.910546	0.09375
GO:0006959	humoral immune response	BP	2	27	67	483	0.029851	0.055901	0.911453	0.074074
GO:0022603	regulation of anatomical structure morphogenesis	BP	1	16	67	483	0.014925	0.033126	0.911987	0.0625
GO:0034364	high-density lipoprotein particle	CC	1	16	67	483	0.014925	0.033126	0.911987	0.0625
GO:0048878	chemical homeostasis	BP	1	16	67	483	0.014925	0.033126	0.911987	0.0625
GO:0001816	cytokine production	BP	1	16	67	483	0.014925	0.033126	0.911987	0.0625
GO:0007165	signal transduction	BP	4	47	67	483	0.059701	0.097308	0.917621	0.085106
GO:0032991	protein-containing complex	CC	12	115	67	483	0.179104	0.238095	0.918846	0.104348
GO:0005839	proteasome core complex	CC	1	17	67	483	0.014925	0.035197	0.924614	0.058824
GO:0004298	threonine-type endopeptidase activity	MF	1	17	67	483	0.014925	0.035197	0.924614	0.058824
GO:0070003	threonine-type peptidase activity	MF	1	17	67	483	0.014925	0.035197	0.924614	0.058824
GO:0022008	neurogenesis	BP	1	17	67	483	0.014925	0.035197	0.924614	0.058824
GO:0001525	angiogenesis	BP	1	17	67	483	0.014925	0.035197	0.924614	0.058824
GO:0016740	transferase activity	MF	1	17	67	483	0.014925	0.035197	0.924614	0.058824
GO:0022607	cellular component assembly	BP	3	39	67	483	0.044776	0.080745	0.929993	0.076923
GO:0044085	cellular component biogenesis	BP	3	39	67	483	0.044776	0.080745	0.929993	0.076923
GO:0007166	cell surface receptor signaling pathway	BP	2	29	67	483	0.029851	0.060041	0.931368	0.068966
GO:0050896	response to stimulus	BP	16	149	67	483	0.238806	0.308489	0.932125	0.107383

GO:0023057	negative regulation of signaling	BP	1	18	67	483	0.014925	0.037267	0.935453	0.055556
GO:0045595	regulation of cell differentiation	BP	1	18	67	483	0.014925	0.037267	0.935453	0.055556
GO:0007399	nervous system development	BP	1	18	67	483	0.014925	0.037267	0.935453	0.055556
GO:0009968	negative regulation of signal transduction	BP	1	18	67	483	0.014925	0.037267	0.935453	0.055556
GO:0051094	positive regulation of developmental process	BP	1	18	67	483	0.014925	0.037267	0.935453	0.055556
GO:0010648	negative regulation of cell communication	BP	1	18	67	483	0.014925	0.037267	0.935453	0.055556
GO:0051248	negative regulation of protein metabolic process	BP	1	18	67	483	0.014925	0.037267	0.935453	0.055556
GO:0002682	regulation of immune system process	BP	3	40	67	483	0.044776	0.082816	0.937608	0.075
GO:0032879	regulation of localization	BP	3	40	67	483	0.044776	0.082816	0.937608	0.075
GO:0009653	anatomical structure morphogenesis	BP	3	40	67	483	0.044776	0.082816	0.937608	0.075
GO:0051179	localization	BP	7	77	67	483	0.104478	0.15942	0.939645	0.090909
GO:0048514	blood vessel morphogenesis	BP	1	19	67	483	0.014925	0.039337	0.944753	0.052632
GO:0042060	wound healing	BP	2	31	67	483	0.029851	0.064182	0.947053	0.064516
GO:0048731	system development	BP	4	52	67	483	0.059701	0.10766	0.951667	0.076923
GO:0001944	vasculature development	BP	1	20	67	483	0.014925	0.041408	0.95273	0.05
GO:0072358	cardiovascular system development	BP	1	20	67	483	0.014925	0.041408	0.95273	0.05
GO:0009617	response to bacterium	BP	1	20	67	483	0.014925	0.041408	0.95273	0.05
GO:0035239	tube morphogenesis	BP	1	20	67	483	0.014925	0.041408	0.95273	0.05
GO:0001568	blood vessel development	BP	1	20	67	483	0.014925	0.041408	0.95273	0.05
GO:0032502	developmental process	BP	5	62	67	483	0.074627	0.128364	0.954581	0.080645
GO:0140096	catalytic activity, acting on a protein	MF	5	63	67	483	0.074627	0.130435	0.959102	0.079365
GO:0072359	circulatory system development	BP	1	21	67	483	0.014925	0.043478	0.959571	0.047619
GO:0009888	tissue development	BP	1	21	67	483	0.014925	0.043478	0.959571	0.047619
GO:0044421	extracellular region part	CC	12	124	67	483	0.179104	0.256729	0.96089	0.096774
GO:0007275	multicellular organism development	BP	4	54	67	483	0.059701	0.111801	0.961293	0.074074
GO:0035295	tube development	BP	1	22	67	483	0.014925	0.045549	0.965434	0.045455
GO:0006629	lipid metabolic process	BP	1	22	67	483	0.014925	0.045549	0.965434	0.045455
GO:0006869	lipid transport	BP	1	22	67	483	0.014925	0.045549	0.965434	0.045455
GO:0034358	plasma lipoprotein particle	CC	1	23	67	483	0.014925	0.047619	0.970458	0.043478
GO:0048646	anatomical structure formation involved in morphogenesis	BP	1	23	67	483	0.014925	0.047619	0.970458	0.043478
GO:0042592	homeostatic process	BP	1	23	67	483	0.014925	0.047619	0.970458	0.043478
GO:1990777	lipoprotein particle	CC	1	23	67	483	0.014925	0.047619	0.970458	0.043478

GO:0032994	protein-lipid complex	CC	1	23	67	483	0.014925	0.047619	0.970458	0.043478
GO:0005615	extracellular space	CC	11	120	67	483	0.164179	0.248447	0.973289	0.091667
GO:0043207	response to external biotic stimulus	BP	1	24	67	483	0.014925	0.049689	0.97476	0.041667
GO:0009607	response to biotic stimulus	BP	1	24	67	483	0.014925	0.049689	0.97476	0.041667
GO:0051707	response to other organism	BP	1	24	67	483	0.014925	0.049689	0.97476	0.041667
GO:0080134	regulation of response to stress	BP	1	25	67	483	0.014925	0.05176	0.978445	0.04
GO:0048856	anatomical structure development	BP	4	60	67	483	0.059701	0.124224	0.980665	0.066667
GO:2000026	regulation of multicellular organismal development	BP	1	26	67	483	0.014925	0.05383	0.981598	0.038462
GO:0051240	positive regulation of multicellular organismal process	BP	1	26	67	483	0.014925	0.05383	0.981598	0.038462
GO:0002376	immune system process	BP	6	80	67	483	0.089552	0.165631	0.982025	0.075
GO:0007596	blood coagulation	BP	1	27	67	483	0.014925	0.055901	0.984296	0.037037
GO:0016477	cell migration	BP	1	27	67	483	0.014925	0.055901	0.984296	0.037037
GO:0050817	coagulation	BP	1	27	67	483	0.014925	0.055901	0.984296	0.037037
GO:0007599	hemostasis	BP	1	27	67	483	0.014925	0.055901	0.984296	0.037037
GO:0050878	regulation of body fluid levels	BP	1	28	67	483	0.014925	0.057971	0.986603	0.035714
GO:0051674	localization of cell	BP	1	29	67	483	0.014925	0.060041	0.988576	0.034483
GO:0061134	peptidase regulator activity	MF	1	29	67	483	0.014925	0.060041	0.988576	0.034483
GO:0048870	cell motility	BP	1	29	67	483	0.014925	0.060041	0.988576	0.034483
GO:0006928	movement of cell or subcellular component	BP	1	30	67	483	0.014925	0.062112	0.990262	0.033333
GO:0040011	locomotion	BP	1	30	67	483	0.014925	0.062112	0.990262	0.033333
GO:0006955	immune response	BP	4	66	67	483	0.059701	0.136646	0.990708	0.060606
GO:0048585	negative regulation of response to stimulus	BP	1	31	67	483	0.014925	0.064182	0.991702	0.032258
GO:0005576	extracellular region	CC	17	182	67	483	0.253731	0.376812	0.992419	0.093407
GO:0032501	multicellular organismal process	BP	7	98	67	483	0.104478	0.202899	0.993183	0.071429
GO:0098772	molecular function regulator	MF	2	47	67	483	0.029851	0.097308	0.994222	0.042553
GO:0051239	regulation of multicellular organismal process	BP	2	48	67	483	0.029851	0.099379	0.995005	0.041667
GO:0009605	response to external stimulus	BP	2	50	67	483	0.029851	0.10352	0.996275	0.04
GO:0006950	response to stress	BP	5	86	67	483	0.074627	0.178054	0.997284	0.05814
GO:0030234	enzyme regulator activity	MF	1	38	67	483	0.014925	0.078675	0.997323	0.026316

BP: biological process; MF, molecular function; CC, cellular component

Table S3. KEGG enrichment analysis

No.	Map_ID	Map_Name	Test	TestAll	Ref	RefAll	Test_per	Ref_per	P value	richFactor
1	ocu04390	Hippo signaling pathway	3	67	6	483	0.044776	0.012422	0.037497	0.5
2	ocu04110	Cell cycle	2	67	4	483	0.029851	0.008282	0.094458	0.5
3	ocu04216	Ferroptosis	2	67	4	483	0.029851	0.008282	0.094458	0.5
4	ocu05134	Legionellosis	2	67	4	483	0.029851	0.008282	0.094458	0.5
5	ocu05160	Hepatitis C	2	67	4	483	0.029851	0.008282	0.094458	0.5
6	ocu04151	PI3K-Akt signaling pathway	3	67	9	483	0.044776	0.018634	0.115756	0.3333333
7	ocu00510	N-Glycan biosynthesis	1	67	1	483	0.014925	0.00207	0.138716	1
8	ocu00514	Other types of O-glycan biosynthesis	1	67	1	483	0.014925	0.00207	0.138716	1
9	ocu00920	Sulfur metabolism	1	67	1	483	0.014925	0.00207	0.138716	1
10	ocu03010	Ribosome	1	67	1	483	0.014925	0.00207	0.138716	1
11	ocu03013	RNA transport	1	67	1	483	0.014925	0.00207	0.138716	1
12	ocu04520	Adherens junction	1	67	1	483	0.014925	0.00207	0.138716	1
13	ocu04672	Intestinal immune network for IgA production	1	67	1	483	0.014925	0.00207	0.138716	1
14	ocu04714	Thermogenesis	1	67	1	483	0.014925	0.00207	0.138716	1
15	ocu04722	Neurotrophin signaling pathway	1	67	1	483	0.014925	0.00207	0.138716	1
16	ocu04919	Thyroid hormone signaling pathway	1	67	1	483	0.014925	0.00207	0.138716	1
17	ocu05100	Bacterial invasion of epithelial cells	1	67	1	483	0.014925	0.00207	0.138716	1
18	ocu05135	Yersinia infection	1	67	1	483	0.014925	0.00207	0.138716	1
19	ocu05140	Leishmaniasis	1	67	1	483	0.014925	0.00207	0.138716	1
20	ocu05164	Influenza A	1	67	1	483	0.014925	0.00207	0.138716	1
21	ocu05225	Hepatocellular carcinoma	1	67	1	483	0.014925	0.00207	0.138716	1
22	ocu05416	Viral myocarditis	1	67	1	483	0.014925	0.00207	0.138716	1
23	ocu04114	Oocyte meiosis	2	67	5	483	0.029851	0.010352	0.143561	0.4
24	ocu04510	Focal adhesion	2	67	6	483	0.029851	0.012422	0.196593	0.3333333
25	ocu05203	Viral carcinogenesis	2	67	7	483	0.029851	0.014493	0.251559	0.2857143
26	ocu00860	Porphyrin and chlorophyll metabolism	1	67	2	483	0.014925	0.004141	0.258438	0.5
27	ocu03040	Spliceosome	1	67	2	483	0.014925	0.004141	0.258438	0.5
28	ocu04015	Rap1 signaling pathway	1	67	2	483	0.014925	0.004141	0.258438	0.5
29	ocu04066	HIF-1 signaling pathway	1	67	2	483	0.014925	0.004141	0.258438	0.5

30	ocu04670	Leukocyte transendothelial migration	1	67	2	483	0.014925	0.004141	0.258438	0.5
31	ocu04971	Gastric acid secretion	1	67	2	483	0.014925	0.004141	0.258438	0.5
32	ocu05161	Hepatitis B	1	67	2	483	0.014925	0.004141	0.258438	0.5
33	ocu05206	MicroRNAs in cancer	1	67	2	483	0.014925	0.004141	0.258438	0.5
34	ocu05412	Arrhythmogenic right ventricular cardiomyopat	1	67	2	483	0.014925	0.004141	0.258438	0.5
35	ocu04145	Phagosome	2	67	9	483	0.029851	0.018634	0.361551	0.2222222
36	ocu04144	Endocytosis	1	67	3	483	0.014925	0.006211	0.361733	0.3333333
37	ocu04210	Apoptosis	1	67	3	483	0.014925	0.006211	0.361733	0.3333333
38	ocu04512	ECM-receptor interaction	1	67	3	483	0.014925	0.006211	0.361733	0.3333333
39	ocu04530	Tight junction	1	67	3	483	0.014925	0.006211	0.361733	0.3333333
40	ocu04810	Regulation of actin cytoskeleton	1	67	3	483	0.014925	0.006211	0.361733	0.3333333
41	ocu04921	Oxytocin signaling pathway	1	67	3	483	0.014925	0.006211	0.361733	0.3333333
42	ocu04977	Vitamin digestion and absorption	1	67	3	483	0.014925	0.006211	0.361733	0.3333333
43	ocu05143	African trypanosomiasis	1	67	3	483	0.014925	0.006211	0.361733	0.3333333
44	ocu05410	Hypertrophic cardiomyopathy	1	67	3	483	0.014925	0.006211	0.361733	0.3333333
45	ocu05414	Dilated cardiomyopathy	1	67	3	483	0.014925	0.006211	0.361733	0.3333333
46	ocu05418	Fluid shear stress and atherosclerosis	1	67	3	483	0.014925	0.006211	0.361733	0.3333333
47	ocu04640	Hematopoietic cell lineage	1	67	4	483	0.014925	0.008282	0.450824	0.25
48	ocu05165	Human papillomavirus infection	1	67	4	483	0.014925	0.008282	0.450824	0.25
49	ocu03320	PPAR signaling pathway	1	67	5	483	0.014925	0.010352	0.52764	0.2
50	ocu04975	Fat digestion and absorption	1	67	5	483	0.014925	0.010352	0.52764	0.2
51	ocu05205	Proteoglycans in cancer	1	67	5	483	0.014925	0.010352	0.52764	0.2
52	ocu05014	Amyotrophic lateral sclerosis	2	67	13	483	0.029851	0.026915	0.558919	0.1538462
53	ocu04141	Protein processing in endoplasmic reticulum	1	67	6	483	0.014925	0.012422	0.59385	0.1666667
54	ocu05132	Salmonella infection	1	67	6	483	0.014925	0.012422	0.59385	0.1666667
55	ocu04611	Platelet activation	1	67	7	483	0.014925	0.014493	0.650898	0.1428571
56	ocu04621	NOD-like receptor signaling pathway	1	67	7	483	0.014925	0.014493	0.650898	0.1428571
57	ocu04979	Cholesterol metabolism	1	67	10	483	0.014925	0.020704	0.778766	0.1
58	ocu05150	Staphylococcus aureus infection	1	67	11	483	0.014925	0.022774	0.810104	0.0909091
59	ocu04610	Complement and coagulation cascades	1	67	28	483	0.014925	0.057971	0.986603	0.0357143

Table S4. The basic information of two single nucleotide polymorphisms (SNPs) of *SELENBP1* and *VCL* identified via genome-wide association study (GWAS).

SNP	Gene	Position (GRCh38)	Reference	Alternate	Minor allele frequency*	Functional consequence
rs10788804	SELENBP1	chr1:151365312	A	G	0.4691	Intron variant
rs3812625	VCL	chr10:73997944	A	G	0.3397	Upstream variant

* The data of minor allele frequency are acquired from gnomAD V3.1.1.
